# Generative Artificial Intelligence in Medical Imaging: Foundations, Progress, and Clinical Translation

**DOI:** 10.34133/research.1029

**Published:** 2025-12-15

**Authors:** Shanshan Wang, Xuanru Zhou, Cheng Li, Shuqiang Wang, Ye Li, Tao Tan, Hairong Zheng

**Affiliations:** ^1^ Paul C. Lauterbur Research Center for Biomedical Imaging, Shenzhen Institutes of Advanced Technology, Chinese Academy of Sciences, Shenzhen, China.; ^2^ University of Chinese Academy of Sciences, Beijing, China.; ^3^ Research Center for Biomedical Information Technology, Shenzhen Institutes of Advanced Technology, Chinese Academy of Sciences, Shenzhen, China.; ^4^ Faculty of Applied Sciences, Macao Polytechnic University, Macao, China.

## Abstract

Generative artificial intelligence (AI) is rapidly transforming medical imaging by enabling capabilities such as data synthesis, image enhancement, modality translation, and spatiotemporal modeling. This review presents a comprehensive and forward-looking synthesis of recent advances in generative modeling—including generative adversarial networks (GANs), variational autoencoders (VAEs), diffusion models, and emerging multimodal foundation architectures—and evaluates their expanding roles across the clinical imaging continuum. We systematically examine how generative AI contributes to key stages of the imaging workflow, from acquisition and reconstruction to cross-modality synthesis, diagnostic support, treatment planning, and prognosis prediction. Emphasis is placed on both retrospective and prospective clinical scenarios, where generative models help address longstanding challenges such as data scarcity, standardization, and integration across modalities. To promote rigorous benchmarking and translational readiness, we propose a 3-tiered evaluation framework encompassing pixel-level fidelity, feature-level realism, and task-level clinical relevance. We also identify critical obstacles to real-world deployment, including limited generalization under domain shift, risks of hallucinated or unreliable features, data scarcity and privacy concerns, as well as stringent regulatory and ethical constraints. Finally, we explore the convergence of generative AI with large-scale foundation models, highlighting how this synergy may enable the next generation of scalable, reliable, and clinically integrated imaging systems. By charting technical progress and translational pathways, this review aims to guide future research and foster interdisciplinary collaboration at the intersection of AI, medicine, and biomedical engineering.

## Introduction

### Motivation and clinical drivers for generative artificial intelligence

**Fig. 1.**
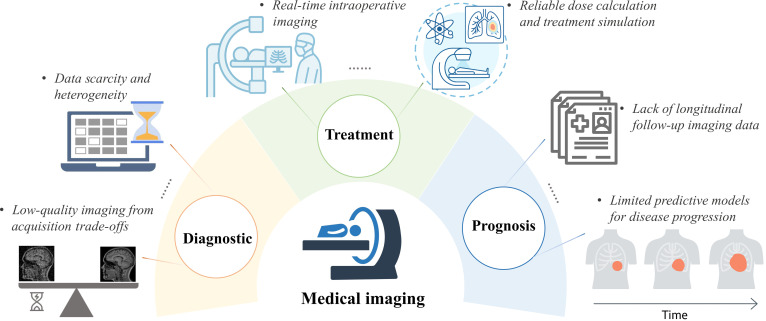
The challenges of medical imaging in clinical workflow.

Medical imaging represents a cornerstone of modern clinical medicine, substantially contributing to all stages of healthcare, encompassing diagnostic assessment, therapeutic planning, and prognostic evaluation. In diagnosis, it enables early disease detection, classification, and quantitative assessment, supporting precision medicine [1]. During treatment, imaging guides surgical procedures, radiation therapy, and minimally invasive interventions, allowing real-time decision-making and improved outcomes [2]. For prognosis, imaging supports longitudinal disease tracking, risk assessment, and treatment response evaluation [3]. Despite remarkable technological progress, several fundamental challenges continue to hinder the full potential of medical imaging in clinical practice, as illustrated in Fig. 1.

A major challenge in medical imaging is the scarcity and heterogeneity of high-quality data. Many modalities are limited by high costs, restricted access, and technical constraints such as slow acquisition, low resolution, and motion artifacts [4]. To mitigate these issues, low-dose computed tomography (CT)/positron emission tomography (PET) and undersampled strategies (e.g., compressed sensing) are used to shorten scans and reduce radiation, but they inevitably introduce noise, artifacts, and resolution loss, driving the need for advanced enhancement techniques like denoising, artifact removal, super-resolution, and reconstruction [5]. In the treatment phase, imaging underpins precision interventions and intraoperative navigation, yet challenges remain in accurate dose calculation, cross-modality synthesis, and real-time tracking. For example, magnetic resonance imaging (MRI)-to-CT translation for radiotherapy can suffer from geometric distortion and loss of detail [2], while intraoperative registration is sensitive to motion and latency, limiting guidance accuracy [6]. From a prognostic perspective, longitudinal imaging is essential for monitoring disease progression, evaluating therapeutic response, and informing risk stratification [3]. However, long-term data collection is often incomplete due to high costs, patient dropout, and inconsistent acquisition protocols across institutions [7].

These limitations underscore the need for generative models that can synthesize missing data, harmonize heterogeneous inputs, and augment incomplete datasets. The clinical demand for such capabilities constitutes a primary motivation for the integration of generative artificial intelligence (AI) into medical imaging workflows.

### Evolution of generative models in medical imaging

In recent years, generative AI has emerged as a transformative force in medical imaging, revolutionizing how imaging data are generated, processed, and analyzed. Since the introduction of GANs in 2014 [8], followed by VAEs [9], diffusion probabilistic models (DPMs) [10], and sequence modeling architectures such as Transformers [11], Mamba [12], autoregressive (AR) models [13], and foundation models (FMs) [14–16], generative AI has demonstrated an unprecedented ability to model complex data distributions and generate high-quality synthetic medical images [17].

The integration of generative AI into medical imaging drives major advances across data augmentation, image restoration, modality translation, real-time synthesis, and prognostic modeling. Generative models synthesize realistic, high-fidelity images to address data scarcity, improving model generalization in disease detection [18,19]. AI-driven restoration enables denoising, artifact removal, super-resolution, and reconstruction, enhancing low-dose and accelerated imaging. GANs and diffusion models support MRI-to-CT, PET-to-MRI, and other translations, aiding multimodal diagnosis and treatment planning [20]. Intraoperatively, real-time generative synthesis refines images for surgical navigation and radiotherapy adaptation [21,22]. For prognosis, longitudinal modeling simulates tumor growth, neurodegenerative progression, and recovery, assisting personalized treatment [23].

Overall, the integration of generative AI across the entire healthcare workflow not only revitalizes traditional medical practices but also establishes a robust foundation for the advancement of precision medicine. However, the full realization of its potential in healthcare remains constrained by several challenges. Chief among these are concerns regarding the reliability and interpretability of generative AI models. The phenomena such as hallucinations [24] can result in inaccurate outputs, while the black-box nature of many models also limits clinical trust. Generalization remains problematic, as performance often declines on unseen data or under varying imaging conditions. Furthermore, high computational demands for training and deployment further constrain scalability in clinical settings [7]. Overcoming these limitations is essential to ensure the safe, effective, and ethical implementation of generative AI in medical applications.

### Review outline and contributions

Generative AI is increasingly applied in medical imaging to address longstanding challenges such as limited data availability, suboptimal image quality, and insufficient temporal information. Overcoming current limitations in model generalizability, interpretability, and clinical validation is essential to advance its real-world deployment. This review aims to provide a comprehensive analysis of recent advancements in medical image generation, with a focus on their clinical applications, evaluation methodologies, and future research directions. The key contributions of this work are as follows:

• *Comprehensive survey of key generative AI models:* We systematically explore the theoretical foundations and practical applications of GANs, VAEs, DPMs, and sequence modeling architectures (Transformers, Mamba, AR models), as well as FMs
• *Integration with the clinical workflow:* We analyze how generative models are applied across acquisition and reconstruction, diagnostic, therapeutic, and prognostic stages, enabling static image synthesis, restoration, dynamic image generation, treatment planning, and disease progression modeling within clinical workflows.
• *Proposal of a multi-level evaluation framework:* We propose a structured evaluation framework that assesses generative models at 3 levels: pixel-level fidelity, feature- and distribution-level consistency, and clinical-level applicability. This framework aims to bridge technical performance with clinical utility and supports standardized benchmarking across tasks.
• *Discussion of challenges, limitations, and future directions:* We examine prevailing challenges that hinder the clinical translation of generative AI, including limited generalizability, high computational demands, insufficient interpretability, and regulatory uncertainty, and discuss their implications for future research and model deployment.

## Key Generative AI Models in Medical Imaging

The core technologies of generative AI in medical imaging primarily include GANs [8], VAEs [9], DPMs [10], and sequence modeling architectures such as Transformers [11], Mamba [12], and AR models [13], as well as FMs [14–16] that unify and transfer knowledge across tasks and modalities. These generative techniques have demonstrated remarkable versatility across a wide range of medical imaging tasks, including image synthesis, quality enhancement, modality translation, image reconstruction, super-resolution generation, and dynamic imaging modeling [5].

**Fig. 2.**
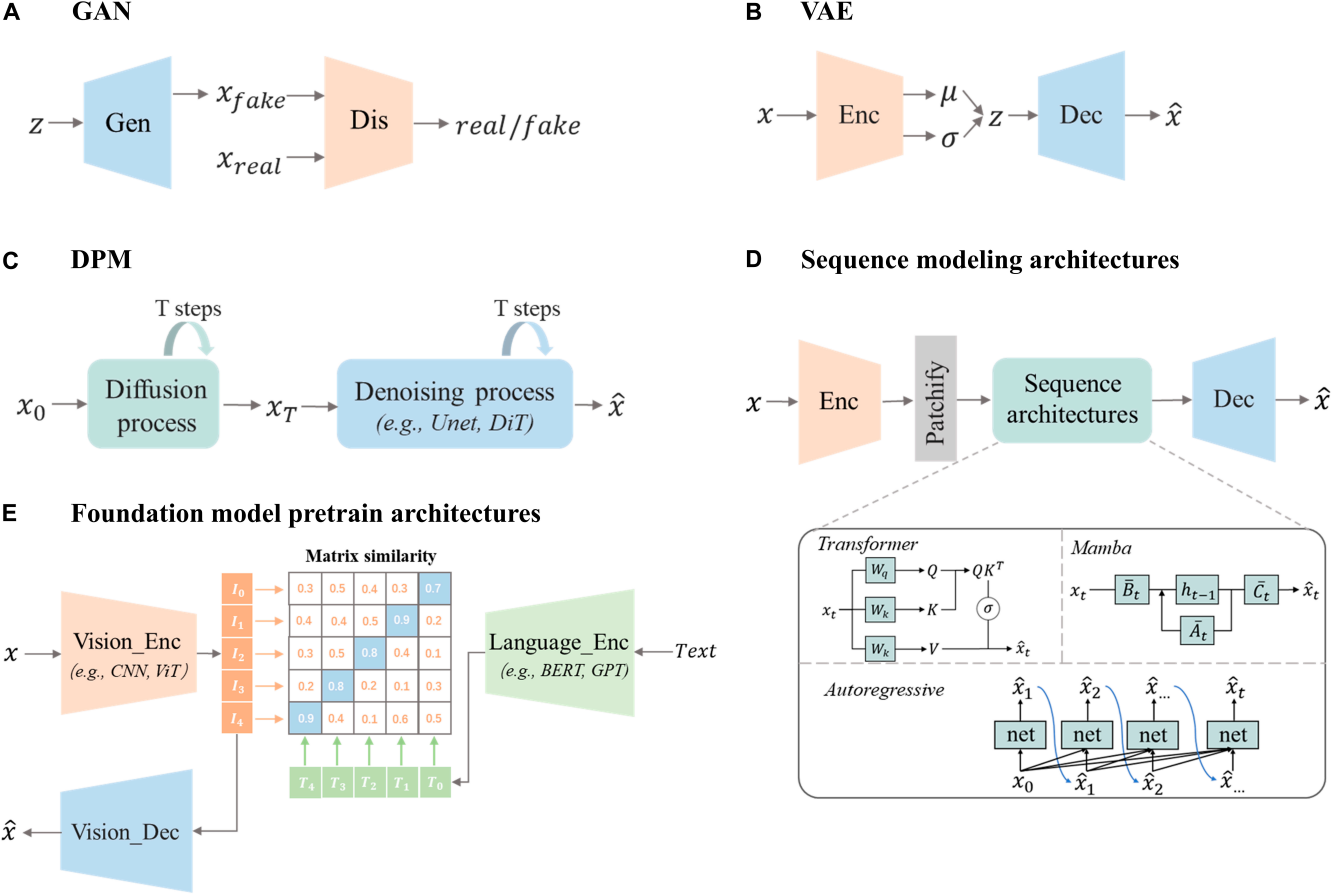
Architectures of generative AI models in medical imaging. (A) Generative adversarial network (GAN) comprising a generator and a discriminator. (B) Variational autoencoder (VAE) with an encoder–decoder structure and latent space mapping. (C) Diffusion probabilistic model (DPM) featuring forward diffusion and a denoising network (e.g., U-Net and DiT). (D) Sequence modeling architectures utilizing Transformer, Mamba, or autoregressive networks on image patches. (E) Foundation model pretraining architectures aligning vision and language encoders.

GANs, proposed by Goodfellow et al. [8], represent a major breakthrough in generative modeling by enabling the creation of realistic data distributions through an adversarial training framework. In the GAN, a generator (*G*) learns to produce synthetic data that closely mimics real samples, while a discriminator (*D*) distinguishes between real and generated data, as shown in Fig. 2A. These networks engage in a minimax game, refining their outputs iteratively to generate high-quality, realistic data. The training process follows the objective:

$${\text{min}}_{G}\;{\text{max}}_{D}\;{𝔼}_{x∼p_{\text{data}}\left(x\right)}\left[\text{log }\;D\left(x\right)\right]+{𝔼}_{z∼p_{z}\left(z\right)}\left[\text{log}\left(1-D\left(G\left(z\right)\right)\right)\right],$$ (1)

where $p_{\text{data}}\left(x\right)$ is the real data distribution, $p_{z}\left(z\right)$is the prior distribution on the latent vector $z,D\left(x\right)$ is the ${\text{discriminator}}^{’}$ sprobability that *x* is real, and $G\left(z\right)$ is the generated image from the latent vector z.

VAEs [9] revolutionized generative modeling by combining variational inference with neural networks. A VAE consists of 2 components: an encoder, which maps input data to a latent space, and a decoder, which reconstructs the data from this latent representation. Figure 2B shows how this structure allows VAEs to capture complex data distributions and generate new samples via latent space sampling. The training objective involves balancing reconstruction loss and Kullback–Leibler (KL) divergence [25], ensuring both accurate reconstruction and smoothness in the latent space. The objective function is expressed as:

$$L={𝔼}_{q_{\phi }\left(z|x\right)}\left[\text{log }\;p_{\theta }\left(x|z\right)\right]-D_{KL}\left(q_{\phi }\left(z|x\right)\|p\left(z\right)\right),$$ (2)

where $q_{\phi }\left(z|x\right)$is the encoder’s approximation of the posterior distribution, $p_{\theta }\left(x|z\right)$ is the decoder’s likelihood of the data given the latent variables, and $p\left(z\right)$ is the prior distribution over the latent space.

DPMs [10], known as denoising DPMs, are a class of generative models inspired by non-equilibrium thermodynamics. They model data generation through a Markov chain that progressively adds Gaussian noise to the data, transforming it into a simple prior distribution, such as a standard normal distribution. The model then learns to reverse this diffusion process by progressively denoising the data, reconstructing the original data from the noisy samples. Mathematically, the forward process is expressed as:

$$q\left(x_{t}|x_{t-1}\right)=N\left(x_{t};\sqrt{1-\beta_{t}}x_{t-1},\beta_{t}I\right),$$ (3)

where $x_{0}$ denotes the original data distribution, $x_{t}$ represents data with *t* step noise added, and $\beta_{t}$ denotes the variance schedule controlling the amount of noise added at each step *t*. The reverse process is defined as:

$$p_{\theta }\left(x_{t-1}|x_{t}\right)=𝒩(x_{t-1};\;\mu_{\theta }(x_{t},\;t),\sum_{\theta }(x_{t},\;t)),$$ (4)

where ​ $\mu_{\theta }$ and $\Sigma_{\theta }$ are the mean and covariance parameters predicted by the neural network with parameters θ. The model is trained to minimize the variational bound on the negative log-likelihood, which can be expressed as:

$$L=E_{q}\left[D_{KL}\left(q\left(x_{T}|x_{0}\right)\|p\left(x_{T}\right)\right)+\sum_{t=1}^{T}\;D_{KL}\left(q\left(x_{t-1}|x_{t},x_{0}\right)\|p_{\theta }\left(x_{t-1}|x_{t}\right)\right)-\text{log }p_{\theta }\left(x_{0}|x_{1}\right)\right],$$ (5)

where $D_{\mathrm{KL}}\;\text{denotes the}\;\mathrm{KL}$ divergence and $p\left(x_{T}\right)\;$is typically chosen as a standard normal distribution.

Transformers [11] have revolutionized deep learning by capturing long-range dependencies via self-attention, allowing them to model global relationships within data, unlike traditional convolutional neural networks (CNNs) that focus on localized receptive fields. The self-attention mechanism computes a sequence’s representation by relating different positions within it, using query (*Q*), key (*K*), and value (*V*) matrices. The attention scores are calculated by the dot product of *Q* and *K*, scaled by the square root of the dimension and passed through a softmax function:

$$\text{Attention(Q, K, V)}=\text{softmax}\left(\frac{QK^{T}}{\sqrt{d_{k}}}\right)V,$$ (6)

The Mamba architecture, built upon state space models (SSMs) [12], has emerged as a transformative framework for medical image synthesis, addressing critical limitations of conventional models like Transformers (quadratic complexity) and CNNs (local-receptive constraints) [26]. At its core, Mamba employs discretized state space equations to model sequential dependencies with linear computational scaling:

$$h_{t}={\bar{A}}_{t}h_{t-1}+{\bar{B}}_{t}x_{t},$$ (7)

$$y_{t}={\bar{C}}_{t}h_{t},$$ (8)

where $h_{t}$ denotes the hidden state,$x_{t}$ is the input, and ${\bar{A}}_{t}$, ${\bar{B}}_{t}$, ${\bar{C}}_{t}$ ​are discretized parameters derived via zero-order hold (ZOH). This formulation enables efficient integration of long-range features while maintaining the fidelity of local details, which is essential for medical imaging applications.

AR models [13] generate images sequentially, predicting each pixel (or voxel) based on the previously generated ones. This sequential dependency modeling has proven highly effective in medical image synthesis, particularly for tasks requiring fine-grained pixel-level detail. By factorizing the joint distribution *p*(*x*) of an image into a product of conditional probabilities, AR models ensure that each generated element maintains consistency with prior context.

$$p\left(x\right)=\prod_{t=1}^{T}\;p\left(x_{t}|x_{<t}\right),$$ (9)

where $x_{t}$ represents the *t*th element (e.g., pixel, patch, or token) in a predefined generation order, and $x_{<t}$ denotes all previously generated elements. For high-dimensional medical images, this sequential dependency is often modeled using neural networks, such as Transformers or CNNs, to parameterize $p\left(x_{t}|x_{<t}\right)$.

FMs [14–16] are typically pretrained on large-scale datasets and designed to generalize across tasks and modalities, often requiring minimal task-specific supervision. The core idea is to bring matching image–text pairs closer together while pushing nonmatching pairs apart. This framework forms the basis of many large-scale pretrained architectures as illustrated in Fig. 2E, enabling models to generalize across tasks with limited supervision and to support applications such as zero-shot classification, report retrieval, and text-guided image synthesis. These models are trained with a variant of the InfoNCE loss:

$$L_{\text{contrast}}=-\frac{1}{N}\sum_{i=1}^{N}\;\text{log}\frac{\text{exp}\left(sim\left(f\left(I_{i}\right),g\left(T_{i}\right)\right)/\tau \right)}{\sum_{j=1}^{N}\;\text{exp}\left(sim\left(f\left(I_{i}\right),g\left(T_{j}\right)\right)/\tau \right)},$$ (10)

where $f\left(I\right)$ and $g\left(T\right)$ are image and text encoders, sim (.) is the similarity $\left(e.g.,\text{cosine}\right)$, and *τ* is the temperature. This contrastive training ensures that paired images and captions have high similarity, enabling zero-shot image classification and retrieval.

After introducing the main architectures and learning principles of generative models, it is helpful to summarize their distinctive strengths and clinical relevance. Each type of generative model has particular advantages and trade-offs in medical imaging (Table 1). GANs and diffusion models both achieve high image fidelity, with GANs offering faster inference and stronger controllability, while diffusion models provide greater stability and diversity. GANs are well suited for image enhancement and cross-modality translation tasks, where fine structural control and realistic detail are essential. DPMs are advantageous for denoising, reconstruction, and super-resolution, which demand stable optimization and anatomical accuracy. VAEs provide interpretable latent spaces and efficient inference, making them valuable for representation learning, anomaly detection, and uncertainty estimation, although their visual realism is relatively limited. Transformers and Mamba architectures capture broad contextual relationships, and Mamba further improves computational efficiency for large volumetric data. These models are particularly useful for dynamic and multi-organ imaging, where long-range spatial or temporal consistency is important. AR models generate data with precise pixel-level control but can be computationally intensive. Their strong local consistency makes them suitable for sequential image generation, such as cine MRI or ultrasound sequences. FMs, trained on large multimodal datasets, extend generalization across diverse tasks and modalities. They show strong potential for text-guided synthesis, report alignment, and multimodal reasoning, supporting integration into clinical workflows.

**Table 1.** Comparative summary of representative generative models in medical imaging, highlighting their key strengths and limitations

Model	Strengths	Limitations
GANs	High fidelity; high controllability; efficient inference	Mode collapse; limited diversity; training instability
VAEs	Latent space interpretability; efficient inference	Low fidelity
DPMs	High fidelity; high diversity; high controllability	Limited inference
Transformers	Long-range dependency; global information; multimodal adaptability	Limited local information; limited computational efficiency
Mamba	Long-range dependency; high computational efficiency; efficient inference	Memory dilution; limited pretraining
Autoregressive models	High fidelity; local information	Error accumulation; memory dilution; limited inference
Foundation models	Cross-task generalization; multimodal understanding; zero-shot task	High training cost; data dependency

Overall, these generative approaches complement one another across the imaging pipeline, from acquisition and reconstruction to diagnosis, treatment planning, and prognosis. Having established the theoretical foundations and comparative advantages of these models, the next section focuses on their practical use in clinical imaging workflows to address real-world challenges in acquisition and reconstruction, diagnosis, treatment, and prognosis.

## Key Applications of Generative AI in Medical Imaging

**Fig. 3.**
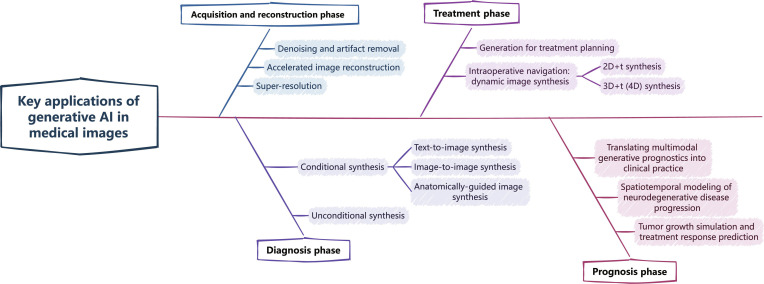
Structural taxonomy of clinical applications of generative AI in medical imaging.

Medical imaging face interconnected challenges: (a) the scarcity of expert-annotated data, (b) heterogeneity in image quality across different imaging devices and institutions, (c) the trade-offs involved in optimizing image quality, and (d) the poor generalizability of models to rare or atypical cases. Building on the methodological insights discussed in Key Generative AI Models in Medical Imaging, generative AI provides practical solutions to these challenges by synthesizing high-quality, realistic medical images and augmenting existing datasets with anatomically consistent variations. These capabilities have led to the rapid adoption of generative AI across multiple stages of the clinical workflow, including acquisition and reconstruction, diagnosis, treatment, and prognosis. As illustrated in Fig. 3, this section provides an overview of current clinical applications of generative AI in medical imaging, aiming to help researchers understand the distribution of these applications and identify potential future research directions.

### Acquisition and reconstruction phase: Enhancing data quality and availability

High-quality medical images are essential for accurate diagnosis, treatment planning, and disease monitoring. However, acquisition constraints, low-dose protocols, patient motion, and hardware limitations have often introduced noise, artifacts, low resolution, or incomplete data, thereby compromising clinical interpretation. To address these challenges, generative AI models have become increasingly pivotal in restoring and enhancing image quality across modalities like CT, MRI, and PET. By leveraging adversarial learning, diffusion-based modeling, and transformer architectures, these models support a wide range of restoration tasks, including denoising, artifact removal, super-resolution, and image reconstruction.

#### Denoising and artifact removal

In low-dose CT, quantum noise and metal artifacts have obscured fine details, limiting lesion detection. Traditional filters reduced noise but blur structures. Recent generative methods performed better: The Poisson flow model [27] suppressed stochastic noise in photon-counting CT, and Wasserstein GANs removed metal-induced artifacts in dental CT, improving implant planning [28]. In ultra-low-dose protocols, CoreDiff [29] has been employed to reconstruct lung images, directly supporting early nodule screening. In PET, the parameter-transferred GAN [30] and a diffusion model [31] reduced noise while preserving standardized uptake values, essential for therapy monitoring. For MRI, which is susceptible to Rician noise and motion artifacts, residual GAN has been shown to improve interslice consistency [32], while a reverse diffusion model [33] enhanced both resolution and noise suppression for clearer anatomical detail. Table S2 presents an overview of recent publications focused on denoising and artifact removal.

#### Accelerated image reconstruction

Reducing acquisition time or dose often leads to sparse or incomplete data, risking loss of critical diagnostic features [34]. A detailed overview of relevant studies can be found in Section S4.1.2 and Table S3. In CT, GAN-based sinogram inpainting restored missing projections, enabling accurate lung screening under limited angles [35], while diffusion priors outperformed iterative methods in detecting subtle hemorrhages [36]. In PET, a CycleGAN [37] improved metabolic feature alignment across modalities, and a VAE-based method [38] reduced PET–MRI registration errors, supporting precise multimodal assessment. Dynamic PET reconstruction with deep generative models restored temporal fidelity essential for therapy monitoring [39]. For MRI, transformer-based and diffusion-informed architectures accelerated cine MRI acquisition while preserving lesion visibility [40,41], and the state-space framework such as Mamba integrated uncertainty quantification for safer clinical decision-making [42].

#### Super-resolution

Limited resolution constrains lesion detection and functional assessment, especially in dynamic organs. Temporal super-resolution has been vital for cardiac or respiratory imaging, where diffusion-based deformation models captured complex motion and suppress irregular artifacts [43]. Spatial super-resolution (SR) addressed structural clarity: GAN-CIRCLE improved CT texture fidelity [44], and diffusion-based dual-stream models enhanced MRI resolution while preserving anatomical consistency [45]. Besides, a diffusion-driven framework further enhanced temporal super-resolution and spatial consistency in 4-dimensional (4D) MRI imaging [46]. These advances (see Table S4) provide higher diagnostic confidence in early disease detection and treatment planning.

Generative models have mitigated noise, artifacts, sparsity, and resolution limits while preserving diagnostic integrity. CT, PET, and MRI all benefit through more reliable reconstructions, faster acquisition, and improved lesion visibility. Super-resolution further enhances anatomical detail and temporal dynamics, reducing the need for higher dose or longer scans. These advances secure image fidelity at the acquisition stage and set the stage for the next focus: “Diagnosis phase: Enriching diagnostic imaging”, where generative AI shifts from restoration to synthesis to address data scarcity and enhance diagnostic utility.

### Diagnosis phase: Enriching diagnostic imaging

Static image synthesis techniques are instrumental in addressing the challenges of data scarcity and domain adaptation. These techniques generate medical images either unconditionally (without explicit constraints) or conditionally (guided by clinical parameters, textual descriptions, etc.), providing solutions to enhance training datasets and improve model generalizability. Below, we categorize these methods based on their underlying approach: unconditional synthesis and conditional synthesis. A detailed overview of relevant studies can be found in Section S4.2, Tables S5 and S6.

Unconditional synthesis generates medical images directly from noise distributions, enabling the creation of diverse datasets without requiring annotations. Early GAN-based approaches demonstrated the feasibility of medical image synthesis but suffered from mode collapse and low resolution, while later improvement introduced structured latent spaces that enhanced fidelity and controllability [47]. More recently, DPMs have become the leading approach, offering stable training and higher diversity. For example, medical diffusion models [48,49] generated high-resolution ultrasound, CT, and MRI data with improved anatomical detail, supporting tumor detection and segmentation. These advances demonstrate unconditional synthesis as a critical tool for addressing data scarcity, although lack of explicit control limits its direct clinical use.

Conditional synthesis: In contrast to unconditional synthesis, which learns image distributions independently of external inputs, conditional synthesis incorporates domain-specific priors such as clinical text, imaging data, anatomical structures, or physiological parameters into the generative process. This improves the relevance, controllability, and diagnostic value of the synthesized outputs.

• *Text-to-image synthesis.* Radiology reports and clinical metadata often contain valuable diagnostic cues but lack paired imaging for direct use. To bridge this gap, latent diffusion models have enabled text-to-image generation, aligning textual findings with synthetic images. For example, Chest-diffusion [50] generated chest x-rays from reports, enriching datasets for rare pathologies and improving interpretability. Extending this idea, MediSyn [51] generalized across modalities, creating diverse synthetic scans guided by textual or clinical prompts. These approaches improve the alignment between clinical documentation and imaging, expanding data availability for diagnostic model training.
• *Image-to-image synthesis.* In clinical workflows, missing or degraded modalities (e.g., unavailable CT in PET/MRI workflows) compromise diagnosis and treatment planning. Image-to-image synthesis has addressed this by translating between modalities while preserving structural fidelity [52]. CycleGAN [53,54] demonstrated the feasibility of bidirectional mappings between CT, PET, and MRI without paired data, while transformer-based models such as ResViT [55] further improved spatial consistency and cross-modality alignment. More recently, diffusion-based methods [29,56] further enhanced anatomical preservation, enabling robust modality completion and zero-shot translation. These methods have directly reduced the impact of incomplete or inconsistent imaging in clinical pipelines.
• *Anatomically guided synthesis.* A persistent limitation of generative synthesis is the risk of anatomically implausible outputs. To overcome this, anatomical priors such as segmentation masks or vascular maps have been embedded into the generation process. For instance, the vascular-guided GAN [57] preserved fine vessel structures in retinal fundus images, while the segmentation-guided diffusion model [58] allowed controllable synthesis across multiple organs and modalities. By integrating structural constraints, these methods enhanced both interpretability and clinical reliability, making synthetic data more suitable for lesion augmentation and rare disease modeling.

Unconditional synthesis has expanded datasets without annotations, using GANs and diffusion models to generate diverse, anatomy-preserving images that strengthen model robustness under data scarcity. Conditional synthesis adds clinical control: Text-driven methods align reports and demographics with synthetic images; image-to-image translation and completion recover missing or degraded modalities; anatomically guided generation enforces structural plausibility for lesion-level augmentation and rare disease scenarios. Together, these approaches move beyond restoration to enrich training distributions, improve domain generalization, and tighten the link between clinical context and image content.

### Treatment phase: Enabling precision interventions

In the treatment phase of clinical care, the integration of generative AI into radiotherapy and intraoperative navigation offers transformative potential for precision medicine. By modeling complex anatomical variations, capturing physiological motion, and supporting real-time clinical decision-making, generative models are increasingly bridging the gap between static preoperative imaging and dynamic, adaptive interventions. This section explores 2 key areas: dose prediction and planning in radiotherapy, and dynamic image synthesis for intraoperative navigation, as illustrated in Table S7.

#### Generation for treatment planning

In radiotherapy, interpatient anatomical variability and tumor motion have complicated precise dose delivery, often risking damage to adjacent organs. Generative models have emerged as powerful tools for predicting individualized dose maps and simulating treatment anatomy. Early frameworks such as DoseNet [59] applied fully convolutional networks to rapidly generate 3D dose distributions, while TransDose [22] introduced transformers to capture long-range spatial dependencies and improve conformity around critical organs. More recently, diffusion-based approaches such as DiffDP [60] have enabled the generation of multiple plausible dose distributions from CT and segmentation inputs, supporting flexible planning in anatomically complex cases. Similarly, MD-dose [61] enhanced both sampling speed and accuracy through its Mamba-based architecture, supporting real-time adaptive planning. Foundation and generative models have extended beyond dose prediction, contributing to imaging tasks like synthetic image generation via a self-improving model [18] and cone-beam computed tomography (CBCT)-based tumor tracking [21], which collectively enhanced adaptive radiotherapy workflows. Collectively, these approaches reduce trial-and-error costs, enhanced personalization, and lay the foundation for real-time adaptive radiotherapy.

#### Intraoperative navigation: Dynamic image synthesis

Real-time intraoperative imaging must capture both anatomy and motion, but conventional acquisitions are constrained by slow speed, radiation dose, and motion artifacts. Generative models have been explored to synthesize dynamic sequences from limited inputs. In cardiac MRI, the GAN-based framework [62] accelerated cine reconstruction while preserving morphology. DragNet [6], a registration-driven method, recovered full cardiac cycles from static frames, reducing motion blur. A cascaded video diffusion model [63] refined motion and texture using semantic cues, producing smoother and more realistic echocardiograms. Multimodal conditioning, for example combining electrocardiogram (ECG) with imaging, has enabled personalized cardiac motion synthesis in the HeartBeat [64]. At the volumetric level, a temporally aware GAN [65] integrated respiratory compensation into dynamic 3D cardiac MRI, effectively reducing motion-induced artifacts. Cross-modal strategies further advanced adaptability in radiotherapy: One study synthesized 4D CT from sparse CBCT [66], while another translated CBCT into 4D MRI [67]. Despite these advances, current approaches still struggle with nonlinear motion and real-time deployment. A recent text-driven method [68] that incorporated disease descriptions into cardiac cine MRI illustrates a promising path toward controllable, pathology-specific motion generation, bridging dynamic imaging with intelligent intervention.

### Prognosis phase: Longitudinal and personalized medicine

Generative medical imaging techniques have demonstrated substantial clinical potential in longitudinal prognostic analysis and personalized medicine, as summarized in Table S8. By leveraging deep modeling of patients’ multi-temporal imaging data, these approaches can simulate dynamic disease progression, predict tissue degenerative changes, and quantify prognostic risk, thereby providing data-driven support for clinical decision-making.

#### Tumor growth simulation and treatment response prediction

Precise modeling of tumor evolution is vital for planning adaptive therapies, yet variability in growth patterns and treatment response limits conventional approaches. A treatment-aware DPM [23] simulated glioma growth from longitudinal MRI and molecular data, improving future tumor prediction accuracy by over 16%. To address incomplete follow-up scans, SADM [69] introduced AR sequence generation, enabling robust modeling despite missing data. Synthetic tumor framework further enhanced radiomics-based survival prediction in glioblastoma, supporting patient-specific radiotherapy [70]. More recently, a CT foundation model (CT-FM) [71], which was trained across multiple malignancies, has achieved state-of-the-art performance on tumor staging and survival prediction, providing a unified prognostic platform that can aid therapy selection and risk stratification.

#### Spatiotemporal modeling of neurodegenerative disease progression

For disorders such as Alzheimer’s disease, monitoring structural brain changes over time is essential for staging and therapy. Generative synthesis of longitudinal MRI has enabled visualization of subtle degenerative trajectories [72]. A hybrid DCGAN–SRGAN framework [73] generated synthetic MRI sequences across disease stages, achieving high classification accuracy and supporting progression modeling. The temporal-aware diffusion model (TADM) [74] further reduced brain volume prediction error by 24% compared with conventional baselines, improving anatomical fidelity in longitudinal imaging. These methods have offered quantitative and visual tools to track disease progression and guide optimal intervention timing.

#### Translating multimodal generative prognostics into clinical practice

Integrating imaging with clinical variables remains a challenge for prognosis. A conditional GAN [75] has been used to synthesize cardiac aging images, improving early detection of diastolic dysfunction, while a diffusion model [76] improved brain volume prediction in Alzheimer’s by 22%. In oncology, FM [18] enhanced breast cancer stratification by increasing human epidermal growth factor receptor 2 (HER2) and epidermal growth factor receptor (EGFR) sensitivity. For cerebrovascular disease, a synthetic CT-based deep model [77] predicted hematoma expansion with an accuracy of 0.84 and specificity of 0.91, supporting early clinical decision-making. Radiomics features extracted from synthetic MRI also improved glioblastoma survival prediction across centers [70]. These advances highlight the clinical value of generative prognostics, although large-scale translation will depend on improving domain adaptation, interpretability, and workflow integration.

By capturing dynamic, multimodal disease trajectories, generative imaging models offer powerful tools for prognosis across tumor, neurological, and cardiovascular domains. Nonetheless, clinical translation at scale requires further work in domain adaptation, temporal modeling, and model interpretability. Future progress in these areas is expected to enhance robustness and generalizability across diverse clinical environments, reinforcing the role of generative models in precision medicine and personalized care.

## Overview of Public Datasets

The rapid development of generative AI in medical imaging has been largely driven by large-scale, high-quality, multi-modal public datasets, which provide both essential training resources and standardized benchmarks for generalization and clinical applicability.

Representative repositories such as UK Biobank [78] and TCIA [79] encompass diverse modalities (MRI, CT, ultrasound, PET, fundus) and tumor types, enabling image synthesis, modality translation, and anomaly simulation. Grand Challenge and Kaggle platforms further facilitate reproducible benchmarking across a wide range of imaging tasks.

For specific anatomical regions, landmark datasets like DeepLesion [80], PreCT-160K [81], TotalSegmentator [82], and BraTS21 [83] offer unprecedented scale or fine-grained annotations, supporting lesion synthesis, longitudinal prediction, and anatomically guided generation. In cardiovascular imaging, EchoNet-Dynamic [63] and ACDC [84] enable dynamic 2D+t/3D+t modeling, while datasets such as AutoPET [85] and HECKTOR [86] facilitate PET-CT fusion for tumor-focused tasks. In neuroimaging, Diff5T [87] provides high-field diffusion MRI with raw *k*-space data, advancing reconstruction and microstructural modeling. Beyond radiology, large-scale resources in pathology (e.g., PatchCamelyon [88] and Quilt-1M [89]), ophthalmology (e.g., OCT2017 [90] and ODIR-5K [90]), and multimodal image–text corpora (e.g., CheXpertPlus [91], MedICaT [92], and Medtrinity-25M [93]) have become indispensable for FMs and vision–language pretraining.

While these datasets have enabled substantial advances, challenges such as domain shift, annotation inconsistency, and limited dynamic or longitudinal data remain. Addressing these gaps through standardization, collaborative curation, and responsible synthetic data integration will be crucial for reliable deployment of generative models in clinical practice (see Section S5 and Table S9 for the full dataset catalog).

## Evaluation Methods for Generative Models in Medical Imaging

**Fig. 4.**
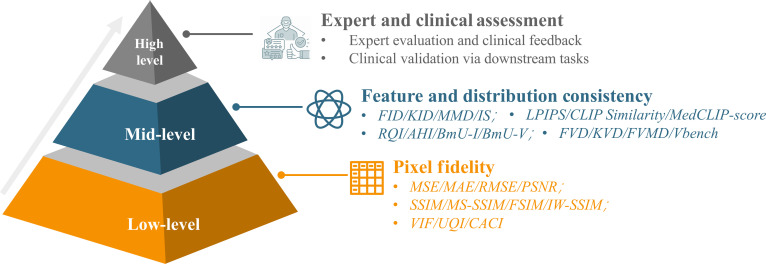
Three-level evaluation pyramid for generative models in medical imaging. This figure illustrates a hierarchical evaluation framework comprising low-level, mid-level, and high-level metrics. The structure emphasizes a progression from basic image quality toward clinical applicability.

Evaluation remains a key challenge for generative AI in medical imaging. Conventional pixel-level metrics often fail to capture anatomical plausibility or clinical utility, while inconsistent standards hinder fair comparison across tasks and modalities. Reliable evaluation is therefore critical for both methodological benchmarking and clinical translation, as highlighted by recent efforts such as the STAGER checklist [94] for standardized reliability assessment and explainable pathology-oriented evaluation frameworks [95]. To address this, we adopt a 3-level hierarchical evaluation framework (Fig. 4) that integrates complementary strategies at different abstraction levels: pixel fidelity, feature and distribution consistency, and clinical relevance. This structure provides a more systematic way to assess image quality, semantic realism, and diagnostic utility.

Low-level evaluation focuses on pixel-wise similarity between generated and reference images. Metrics such as mean square error (MSE), mean absolute error (MAE), peak signal-to-noise ratio (PSNR), and root mean square error (RMSE) are widely used in reconstruction and denoising tasks but correlate poorly with human perception. Structural metrics like SSIM [96], MS-SSIM [97], and FSIM [98] incorporate luminance, contrast, and texture, offering improved alignment with visual perception. Advanced variants such as IW-SSIM [99] and CACI [100] further emphasize diagnostically relevant regions. These metrics effectively assess structural integrity and visual fidelity in tasks like denoising, reconstruction, and compression. However, their focus on low-level features limits detection of semantic inconsistencies, anatomical errors, and clinically irrelevant content critical to evaluating diagnostic utility.

Mid-level evaluation assesses feature-level similarity and distribution alignment using pretrained models. Metrics such as FID [101], KID [102], MMD [103], and Inception Score evaluate global structure and diversity but depend on the domain of the feature extractor. Perceptual similarity measures like LPIPS [104], and multimodal embedding scores such as CLIP Similarity [105] and MedCLIP-score [106], help detect hallucinations by assessing image–text coherence. Other metrics like RQI [100], AHI [100], and BmU [107] evaluate restoration quality and semantic alignment, while FVD [108] and FVMD [109] extend assessment to temporal coherence in dynamic imaging. Mid-level evaluations bridge pixel fidelity and clinical relevance, offering insights into perceptual and statistical realism. However, their effectiveness depends on pretrained model alignment and task complexity, making them more useful when combined with low- and high-level assessments for comprehensive validation.

High-level evaluation represents clinically critical stage in assessing generative models for medical imaging. Unlike lower-level metrics that assess pixel accuracy or feature similarity, this stage focuses on clinical applicability in tasks such as diagnosis, treatment planning, and disease monitoring. It includes 2 main forms: expert assessment, where radiologists evaluate realism and anatomical plausibility, and downstream task evaluation, which measures the impact of synthetic data on segmentation, classification, or regression performance. In expert evaluations, interactive feedback from clinicians has been shown to substantially enhance diagnostic realism. For instance, in MINIM [18], iterative refinement incorporating radiologist scoring increased the proportion of clinically acceptable images from 70.75% to 89.25%. The second form of high-level evaluation involves downstream task assessment, where the quality of synthetic images is indirectly validated through performance gains in specific clinical applications. Synthetic images have demonstrated strong potential to preserve clinically relevant features and enhance model robustness. For example, incorporating synthetic MRIs improved tumor segmentation dice scores by about 2.2% [110], while synthetic breast cancer images increased HER2-positive tumor classification accuracy from 79.2% to 94.0% in data-limited scenarios [18].

Evaluating generative models in medical imaging requires balancing visual quality with clinical relevance. Pixel-level metrics are easy to compute but miss perceptual and diagnostic accuracy. Feature-based measures like FID and LPIPS better capture semantics but depend on pretrained model choice and dataset size. Expert reviews offer direct diagnostic insight but remain subjective. Combining complementary strategies is essential: Objective metrics, expert ratings, and task-based validation together ensure technical and clinical utility. For example, MINIM [18] integrates all 3, showing how multi-level evaluation supports models that are both statistically robust and clinically meaningful, highlighting the need for standardized, multi-faceted protocols for real-world deployment.

## Discussion and Future Directions

Generative models in medical imaging face considerable hurdles from both technical and clinical perspectives. Technically, these models grapple with challenges such as limited generalization, high computational demands, opaque decision-making processes, dependence on high-quality data, and the risk of generating misleading “hallucinations”. Clinically, concerns revolve around ensuring model reliability and trustworthiness, enhancing interpretability for informed decision-making, seamlessly integrating AI into existing workflows, and addressing regulatory and ethical constraints. These challenges highlight the intricate balance between advancing AI-driven imaging technologies and meeting the stringent requirements of clinical practice.

### Technical challenges and limitations

#### Limited generalization and bias

Generative models often perform well on benchmark datasets but struggle when applied to different institutions, modalities, or demographics due to training data bias. For example, models trained mainly on adult CT scans may generalize poorly to pediatric or low-resource settings. Addressing this requires more diverse and representative data, including rare diseases and multi-center cohorts. FMs have shown potential by synthesizing multi-organ or cross-modality images from text prompts and generalizing to unseen domains. However, eliminating bias remains difficult, and careful dataset curation is necessary to prevent reinforcing healthcare disparities [111].

#### High computational demands

Modern generative models like GANs and diffusion models are resource-intensive, especially for high-resolution or 3D images. This limits their use in time-sensitive clinical settings such as emergency or intraoperative care. Optimizing efficiency is critical—recent works [112] on model compression, architectural improvements, and knowledge distillation aim to reduce inference time without compromising quality. At the same time, real-time performance is especially critical for clinical tasks such as intraoperative guidance and bedside diagnostics, where delays of even a few seconds can affect decision-making. So, improving computational efficiency will be essential for enabling the widespread adoption of generative models in routine clinical workflows.

#### Lack of interpretability

Many generative models operate as black boxes, offering little transparency into how specific outputs are produced. For instance, a model might generate a nonexistent tumor with no explanation, raising concerns in fields like radiology where trust and accuracy are vital. Scientifically, the internal logic of these models remains opaque; clinically, their lack of transparency hinders adoption. To address this, researches are exploring the use of attention maps [113], saliency visualization [114], and causal inference [115] that combine deep learning with more interpretable components. As a result, there is increasing demand for explainable AI methods that clarify which features the model has relied on and how they influenced the output.

#### Data scarcity and privacy

High-quality annotated medical images are essential for training robust models, yet access is often restricted by privacy laws and institutional policies. Datasets covering rare or underrepresented conditions are especially limited. Although synthetic data may offer partial relief, initial training still requires real-world clinical input [17]. Federated learning [116] has emerged as a privacy-preserving approach, allowing models to learn from distributed data sources without sharing sensitive information. Nevertheless, federated learning presents its own technical challenges, such as communication overhead, inconsistency in data quality, and difficulties in synchronizing model updates across sites.

#### Hallucinations and uncertainty

One major risk of generative models in medical imaging is the creation of hallucinated features—structures that appear realistic but are not present in the original image. These hallucinations can be subtle and may not be detected by standard evaluation metrics, yet they carry notable clinical risk. To manage this, researchers [117,118] are developing uncertainty estimation methods such as confidence maps, Bayesian modeling, and ensemble predictions to highlight unreliable regions. Besides, some researchers also explore statistical indicators like a hallucination index [24] to quantify the likelihood of fabricated content. Reducing these risks requires improved training strategies, including the use of diverse datasets and regularization techniques that promote anatomical fidelity. While early results are promising, the reliable detection and prevention of hallucinations in complex, real-world settings remain an open challenge.

### Clinical challenges and limitations

#### Reliability and trustworthiness

Clinicians’ primary concern is whether AI-generated images and results can be trusted for diagnosis and treatment planning. Medical decisions often rely on subtle findings, and errors such as missing a tumor or adding a false lesion can have serious consequences. Even infrequent mistakes may undermine confidence in the system. Thus, generative models must ensure not only accuracy but also consistent performance in rare or high-risk cases. Studies conducted in recent years highlight clinicians’ openness to AI while also emphasizing the importance of understanding its failure modes [119]. Maintaining a human-in-the-loop approach, where AI augments rather than replaces expert judgment, remains essential until reliability is firmly established.

#### Explainability for decision-making

Clinicians and regulatory bodies increasingly demand that AI decisions be explainable. For generative models, this means clarifying how outputs are produced—such as why a lesion is synthesized or how an MRI is converted to a CT. Explainability is tied closely to the interpretability issues discussed above, but here the emphasis is on the end-user perspective. For instance, if a generative model highlights an area on a PET scan as malignant (by enhancing it or annotating it), the oncologist will need to understand the basis for that suggestion—was it a particular texture, intensity pattern, or a correlation with other data? Without such context, the physician cannot confidently incorporate the AI’s output into their decision.

#### Integration into clinical workflow

Even highly capable generative models may have limited clinical value if they cannot be integrated seamlessly into existing workflows. Hospitals and imaging centers depend on established systems like radiology information platforms and standardized diagnostic protocols. Introducing such tools raises practical concerns: Can the model deliver real-time analysis during image acquisition? Is it compatible with hospital information technology (IT) infrastructure for secure access and storage? Does it create delays or add steps for clinicians? Interoperability and intuitive design are therefore essential in clinical workflows.

#### Regulatory and ethical constraints

Generative AI in medicine must adhere to strict regulatory and ethical standards. Unlike fixed-function devices, they can evolve or behave unpredictably, complicating approval. Recent regulations, such as the EU AI Act [120], treat diagnostic AI as high risk and require transparency, human oversight, and risk controls. Nevertheless, several practical concerns remain unresolved. One key issue is liability—when a model produces an erroneous output that leads to clinical misjudgment, the allocation of responsibility among developers, institutions, and clinicians remains unclear. In addition, the ethical use of synthetic images raises ongoing questions regarding patient consent, data provenance, and the potential misuse of generated data. To mitigate these challenges, current regulatory trends emphasize human accountability, traceability, and continuous post-deployment monitoring of high-risk AI systems. Strengthening such safeguards will be critical to ensuring that generative models are implemented responsibly and maintain clinical trust.

### Toward multimodal FMs

As regulatory frameworks and clinical governance continue to shape the responsible use of generative AI, research is simultaneously advancing toward more scalable and generalizable paradigms. FMs are poised to redefine medical image generation by offering unified and transferable solutions across the clinical continuum. Pretrained on large and diverse datasets, they exhibit remarkable generalization and zero-shot capabilities, enabling applications across multiple imaging modalities and clinical tasks. Most current medical imaging FMs remain vision-based, trained on large-scale CT, MRI, ophthalmic, and digital pathology datasets to learn generalizable anatomical representations [121]. Recent efforts have begun to integrate text-guided or multimodal objectives to enhance semantic consistency and interpretability while maintaining a vision-centered backbone. Table Table 2 lists related publications on FMs in clinical medical imaging.

**Table 2.** Summary of publications on foundation models in clinical medical imaging

Publication (year)	Model	Application	Loss function	Link
MedicalDiffusion (2023) [122]	Diffusion model	CT foundation model	Denoising diffusion loss	√
MedDiff-FM (2024) [123]	Diffusion model	CT foundation model	Denoising diffusion loss	–
RETFound-DE (2025) [125]	Diffusion model	Retinal foundation model	Denoising diffusion loss	√
RoentGen (2024) [127]	Diffusion model	Chest x-ray-text foundation model	Denoising diffusion loss	√
MINIM (2024) [18]	Diffusion model	OCT/CT/x-ray/MRI foundation model	Denoising diffusion loss	√
BME-X (2024) [5]	CNN	MRI foundation model	Cross-entropy loss, MSE loss	√
Triad (2025) [124]	Transformer, VAE	MRI foundation model	L1 loss, log-ratio loss	√
TUMSyn (2025) [128]	Transformer	MRI-text foundation model	Contrastive loss, MSE loss	√
BEPH (2025) [126]	Transformer	Pathology foundation model	MSE loss	√
Prov-GigaPath (2024) [16]	Transformer	Pathology foundation model	Contrastive loss, MSE loss	√
MONET (2024) [130]	Transformer	Image–text foundation model	Contrastive loss, cross-entropy loss	√
MaCo (2024) [14]	Transformer	Radiography–reports foundation model	InfoNCE loss, MAE loss	√

In CT, MedicalDiffusion [122] improved downstream segmentation accuracy from a dice score of 0.91 to 0.95 by generating large-scale synthetic data for self-supervised pretraining, while MedDiff-FM [123] achieved better performance across anatomical regions, reaching an overall dice of 0.84 with enhanced image fidelity and structural consistency. In MRI, BME-X [5] and Triad [124] jointly optimized segmentation, classification, and registration within a unified 3D MRI framework, with Triad improving segmentation, classification, and registration performance by 2.51%, 4.04%, and 4.00%, respectively, across 25 downstream datasets. In ophthalmology, RETFound-DE [125] showed that data-efficient pretraining on limited fundus datasets augmented with synthetic images, achieving strong cross-center generalization in diabetic retinopathy screening with an area under the receiver operating characteristic curve (AUROC) of 0.8029 versus 0.7669 for RETFound in external evaluation. In pathology, BEPH [126] trained on over 11 million whole-slide patches and showed strong label efficiency and clinical relevance, maintaining competitive performance with only 50% of the training data and improving the survival prediction C-index by 1.1 to 5.5% across 6 cancer types. Prov-GigaPath [16] scaled to 1.3 billion tiles, setting new benchmarks across 26 pathology tasks, and achieved a 3.3% improvement in AUROC and an 8.9% increase in area under the precision–recall curve (AUPRC) for pan-cancer mutation prediction across 18 biomarkers. These advances demonstrate that visual FMs can extend beyond image-level recognition to support a wide range of clinically meaningful decision-making tasks.

More recently, the integration of textual and imaging modalities has further advanced interpretability and clinical usability, particularly in cross-modality task completion. RoentGen [127] exemplifies this trend by successfully generating realistic chest x-rays directly from radiology reports. Fine-tuning improved image fidelity from a baseline FID of 19.5 to 3.6 after 60,000 training steps, and radiologist evaluation confirmed the model’s ability to produce clinically consistent projections. MaCo [14] further strengthened image–text alignment through masked contrastive learning on a Vision Transformer backbone. On the Radiological Society of North America (RSNA) dataset detection benchmark, it outperformed the ViT-CLIP baseline under both 10% and 100% annotation settings. In neuroimaging, TUMSyn [128] demonstrated accurate synthesis of T1-weighted brain images for Alzheimer’s disease (AD) analysis, maintaining 86% of the hippocampal-volume difference between AD and control subjects, thereby supporting volumetric assessment in zero-shot settings. Extending to multi-organ and multi-modal tasks, MINIM [18] integrated multimodal pretraining to synthesize high-fidelity CT, MRI, and optical coherence tomography (OCT) from partial inputs or clinical prompts, advancing diagnosis, report generation, and cross-modality synthesis. Collectively, these studies demonstrate how vision–language FMs can perform clinically meaningful cross-modality generation and integration, highlighting their scalability and potential applicability in real-world healthcare workflows.

Overall, the development of multimodal FMs marks a key step toward scalable and interpretable medical AI. By combining imaging, textual, and clinical information within a shared pretraining structure, these models can provide generalized feature representations that support diverse clinical applications—from diagnosis and treatment planning to prognostic modeling. However, remaining challenges include limited availability of well-annotated multimodal datasets, the computational burden of large-scale training, and the need for transparent and regulation-compliant deployment. Future research is expected to explore federated, privacy-preserving pretraining, efficient domain adaptation, and standardized evaluation frameworks to ensure safety, reliability, and equity in real-world healthcare environments.

## Outlook

Generative models offer great promise in medical imaging, enabling data augmentation, modality translation, and disease progression simulation. But their deployment in real-world clinical environments remains limited. This limitation arises from a combination of unresolved technical and clinical challenges. On the technical side, generative models continue to struggle with generalization across institutions and modalities, high computational requirements, limited interpretability, reliance on sensitive annotated data, and the risk of producing hallucinated features. Clinically, concerns remain regarding the reliability of model outputs, the transparency required for informed decision-making, the seamless integration of AI tools into established workflows, and adherence to evolving regulatory and ethical standards.

In response to these challenges, future developments are expected to extend beyond current FMs toward world models and digital twins, capable of simulating physiological processes and individualized disease trajectories in a dynamic and interpretable manner. The incorporation of multimodal information, combining imaging, text, and genomics, will be central to this evolution, enabling more holistic and personalized understanding of health and disease.

To realize this vision, future research must address persistent challenges related to generalizability, computational efficiency, reliability, and interpretability. Moving from research prototypes to widespread clinical adoption will require models that are robust to data heterogeneity, informed by anatomical and physiological priors, and capable of providing uncertainty quantification [129]. Improving model transparency through explainable design will be essential for fostering clinical trust, while strategies such as multi-task learning and domain adaptation can enhance efficiency and robustness across diverse imaging settings. Moreover, enabling real-time inference will further support applications in image-guided interventions and emergency diagnostics. As these technologies mature, ensuring trustworthy and well-governed synthetic imaging will become increasingly important. This includes establishing robust hallucination detection mechanisms, transparent ethical oversight, and standardized evaluation frameworks to guarantee reliability and fairness. Achieving these goals will require the development of large-scale, multi-institutional FMs and their seamless integration into clinical systems. Continued collaboration among technical, clinical, and regulatory communities will be crucial to ensure that generative models meet the rigorous standards required for safe, effective, and ethical use in healthcare. We hope that this review can serve as a valuable resource for researchers and practitioners and inspire continued innovation in this rapidly advancing field.
