# Supplementary material for: Generative Artificial Intelligence in Medical Imaging: Foundations, Progress, and Clinical Translation
Source: Research (Wash D C). 2025 Dec 15;8:1029. doi: 10.34133/research.1029 (PMC12703019; doi:10.34133/research.1029)
Supplement: Supplementary 1 — Supplementary Text Figs. S1 and S2 Tables S1 to S9 [file research.1029.f1.docx]

**Supplementary**

1. **More Details on Review Outline and Contributions**

***Search Criteria.*** This review is based on a systematic survey of recent advancements in medical image generation. We conducted a systematic literature search using PubMed, Scopus, Google Scholar, and DBLP databases to identify relevant articles published from 2019 to 2025 February. The search terms included combinations such as ("medical imag*" OR "MRI" OR "PET" OR "CT" OR "US") AND ("genera*" OR "synth*" OR "pseudo*") AND ("diagnosis" OR "treatment" OR "prognosis") AND "deep learning" in the title, abstract, or keywords. This strategy aimed to capture a broad range of studies across peer-reviewed journals, conference papers, and preprints.

| 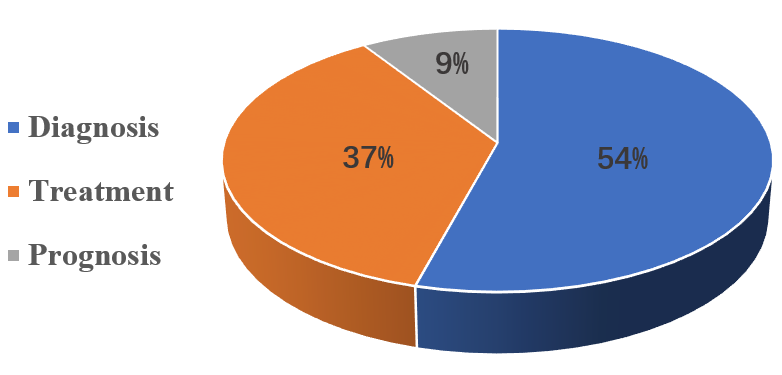 | 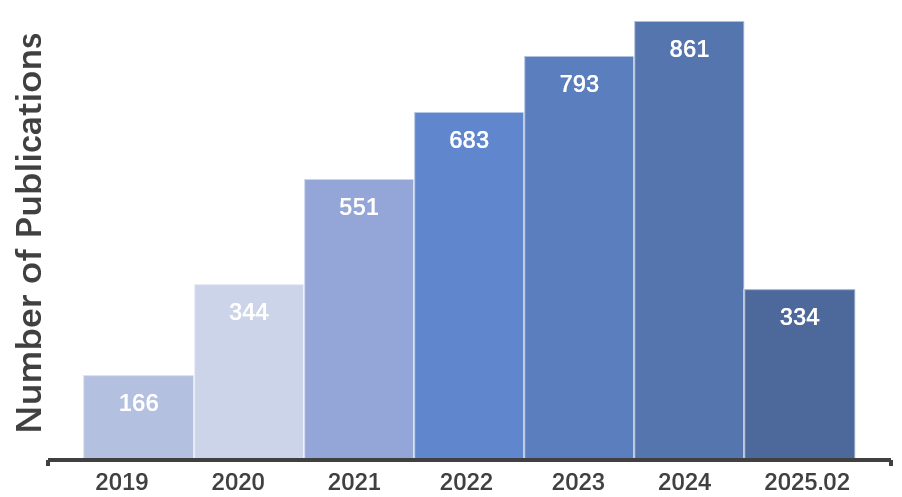 |
| --- | --- |
| (a) | (b) |

**Figure S1.** **Statistics of the generative AI models in medical imaging.** (a) Statistics of Publications in Clinical Workflow: Diagnosis, Treatment, and Prognosis. (b) Categorization by year of publication (2019*-*2025, sourced from PubMed).

1. **More Details on Overview of Related Survey**

In recent years, with the burgeoning development of generative AI in the field of medical images, a multitude of surveys have been published to provide overviews. Table S1 categorizes these surveys into technology-oriented and task-oriented perspectives. The technology-oriented surveys focus on key generative models, including GANs[131–136], VAEs[137,138], diffusion models (DPMs)[139–143], and sequence modeling architectures (e.g., Transformers, Mamba, autoregressive models)[144–147], and foundation models[14–16,127,148], summarizing their core principles and applications. And the task-oriented surveys emphasize practical implementations such as data augmentation[20,149–153], modality translation[134,154–157], image restoration[34,158–161], evaluation of generated results and so on[162–165]. While these surveys offer valuable insights, they tend to either concentrate on theoretical advancements in generative models or focus on specific application scenarios. However, a comprehensive perspective that systematically maps the applications of generative AI across the entire clinical workflow, including diagnosis, treatment, and prognosis, has not been fully explored. Moreover, existing evaluations of generative models in medical imaging are often fragmented, relying on conventional image quality metrics without adequately considering clinical interpretability or downstream utility. The absence of a standardized, multi-tiered evaluation framework further impedes the clinical adoption of generative AI, raising concerns about the reliability and trustworthiness of synthesized medical images.

To address these issues, we provide a structured and comprehensive examination of generative AI in medical imaging, emphasizing its integration across different clinical phases, including acquisition and reconstruction, diagnosis, treatment, and prognosis. We systematically explore the role of key generative models, including GANs, VAEs, diffusion models, sequence modeling architectures, and foundation models in these phases. Additionally, we propose a three-tiered evaluation framework encompassing low-level image fidelity, mid-level feature consistency, and high-level clinical relevance to ensure a more standardized assessment of generative models. Finally, we discuss the current challenges, limitations, and future directions of generative AI in medical imaging, highlighting key considerations for advancing these technologies toward real-world clinical applications.

**Table S1**. Overview of Generative AI Surveys in Medical Imaging.

| **Category** | **Subcategory** | **Publication** | **Core Content** |
| --- | --- | --- | --- |
| **Technique-Oriented Surveys** | **GANs** | [131–136] | -**Characteristics**: Adversarial training mechanism  -**Applications**: Modality synthesis, data augmentation, image denoising |
|  | **VAEs** | [137,138] | -**Characteristics**: Latent space design, probabilistic generation, and feature disentanglement  -**Applications**: Data augmentation, image generation and analysis |
|  | **DPMs** | [139–143] | -**Characteristics**: Progressive denoising process, integration of physical priors  -**Applications**: High-fidelity image synthesis, data augmentation, image denoising/artifact removal, reconstruction |
|  | **Sequence Modeling Architectures** | [144–147] | -**Characteristics**: Transformers (global context modeling), Mamba (long-sequence processing), autoregressive generation  -**Applications**: Time-series prediction, reconstruction, dynamic image generation |
| **Task-Oriented Surveys** | **Data Synthesis & Augmentation** | [20,149–153] | -Multimodal data generation  -2D/3D data synthesis  - Scarce data augmentation |
|  | **Modality Translation** | [134,154–157] | -Pseudo-CT generation  -Cross-modality translation |
|  | **Image Restoration** | [34,158–161] | -Low-dose CT/PET reconstruction  -Undersampled MRI reconstruction, fast MRI imaging  - Super-resolution generation |
|  | **Evaluation & Ethics** | [162–165] | -Generated image reliability assessment (generalizability and interpretability)  -Ethical risks of synthetic data |

1. **More Details** **on Key Generative AI Models in Medical Imaging**

Here we develop in more details on key generative AI models in medical imaging.

- 1. **Generative Adversarial Networks**

Generative Adversarial Network (GANs), proposed by Ian Goodfellow et al. in 2014[8], represent a significant breakthrough in generative modeling by enabling the creation of realistic data distributions through an adversarial training framework. In the GAN, a generator (G) learns to produce synthetic data that closely mimics real samples, while a discriminator (D) distinguishes between real and generated data, as shown in Figure 2(a). These networks engage in a minimax game, refining their outputs iteratively to generate high-quality, realistic data. The training process follows the objective:

$\min_{G} \max_{D} \mathbb{E}_{x\sim p_{\text{data }}(x)}\left[ \log D\left( x \right) \right]+\mathbb{E}_{z\sim p_{z}\left( z \right)}\left[ \log\left( 1-D\left( G\left( z \right) \right) \right) \right],$ (1)

where $p_{\text{data }}(x)$ is the real data distribution, $p_{z}\left( z \right)$ is the prior distribution on the latent vector $z$, $D\left( x \right)$ is the discriminator’s probability that $x$ is real, $G\left( z \right)$ is the generated image from the latent vector $z$.

The field of GAN-based medical image generation has experienced sustained growth, with a steady increase in published studies since the introduction of GANs. As research advances, various GAN variants have been developed, each offering distinct advantages for medical imaging. Deep Convolutional GAN (DCGAN)[166] improves spatial feature learning and stabilizes training by replacing fully connected layers with convolutional operations. CycleGAN[167] enables modality conversion without requiring paired datasets, making it useful for MRI-to-CT synthesis in radiation therapy planning[168] and improving treatment workflows. StyleGAN[169], with its style-based architecture, allows fine-grained control over image features, making it effective for generating highly realistic and diverse medical images**.** Researchers frequently use it in cross-modality analysis, such as generating CT images with MRI-like textures[170], and for creating synthetic datasets that improve model generalization across different imaging systems and clinical environments[171].

Despite these advancements, GANs still face several challenges that hinder their clinical adoption. Training instability and mode collapse can result in inconsistent outputs with limited diversity, while the high computational demands of adversarial training add to the complexity of deployment. Furthermore, the lack of a clearly structured latent space constrains the ability to perform controlled image modifications, making it challenging to maintain anatomical accuracy and pathological consistency, both of which are critical for clinical applicability. Furthermore, concerns regarding image diversity and reproducibility highlight the need for further refinements to enhance the robustness, interpretability, and real-world applicability of GAN-generated medical images.

- 1. **Variational Autoencoders**

Variational Autoencoders (VAEs)[9], revolutionized generative modeling by combining variational inference with neural networks. A VAE consists of two components: an encoder, which maps input data to a latent space, and a decoder, which reconstructs the data from this latent representation. Figure 2(b) shows how this structure allows VAEs to capture complex data distributions and generate new samples via latent space sampling. The training objective involves balancing reconstruction loss and Kullback-Leibler (KL) divergence[25], ensuring both accurate reconstruction and smoothness in the latent space. The objective function is expressed as:

$\mathcal{L=}\mathbb{E}_{q_{\phi}(z\mid x)}\left[ \log p_{\theta}(x\mid z) \right]-D_{KL}\left( q_{\phi}(z\mid x)\|p(z) \right),$ (2)

where $q_{\phi}(z\mid x)$ is the encoder's approximation of the posterior distribution, $p_{\theta}(x\mid z)$ is the decoder's likelihood of the data given the latent variables, and $p(z)$ is the prior distribution over the latent space.

In medical imaging, Variational Autoencoders (VAEs) have been widely used for tasks such as image synthesis and cross-modality translation. Over time, various VAE variants have been developed to address specific challenges and improve performance. β-VAE[172] , for instance, introduces a hyperparameter β to enhance latent space disentanglement, allowing each dimension to represent distinct semantic features. This has proven effective in brain MRI analysis, where it helps distinguish Alzheimer’s-related atrophy from age-related changes[173]. Conditional VAE (CVAE) [174] incorporates conditional variables, such as disease labels or anatomical landmarks, enabling controlled generation of medical images. This capability is particularly valuable in cross-modality synthesis and rare disease research. Vector Quantized VAE (VQ-VAE)[175] discretizes the latent space into a finite codebook, improving image quality and facilitating applications such as low-dose CT reconstruction[176] and 4D heart MRI sequences generation[177]. Additionally, the Hybrid VAE-GAN[178] combines VAE's structured latent space modeling with the adversarial training of GANs to enhance image clarity, with notable applications in glioma MRI synthesis.

For VAEs, the KL divergence constraint can lead to over-smoothing in the latent space, often resulting in blurry image generation. VQ-VAE alleviates this by introducing a discrete codebook, while Hybrid VAE-GAN enhances texture details. However, maintaining anatomical alignment in generated images remains difficult, often necessitating anatomical prior losses or segmentation masks to preserve structural consistency. Additionally, training 3D VAEs on large volumetric datasets requires substantial computational resources. A common[179] strategy to improve efficiency is hierarchical training, where models are initially trained in 2D before being progressively fine-tuned for 3D applications. However, this approach may lead to incomplete spatial representation learning and suboptimal adaptation when transferring from 2D to 3D domains.

- 1. **Diffusion** **Probabilistic Models**

Diffusion Probabilistic Models (DPMs)[10], known as denoising diffusion probabilistic models, are a class of generative models inspired by non-equilibrium thermodynamics. They model data generation through a Markov chain that progressively adds Gaussian noise to the data, transforming it into a simple prior distribution, such as a standard normal distribution. The model then learns to reverse this diffusion process by progressively denoising the data, reconstructing the original data from the noisy samples. Mathematically, the forward process is expressed as:

$q\left( x_{t}\mid x_{t-1} \right)\mathcal{=N}\left( x_{t};\sqrt{1-\beta_{t}}x_{t-1},\beta_{t}I \right),$ (3)

where $x_{0}$​ denotes the original data distribution, $x_{t}$ represents data with t step noise added, $\beta_{t}$ denotes the variance schedule controlling the amount of noise added at each step $t$. And the reverse process is defined as:

$p_{\theta}\left( x_{t-1}\mid x_{t} \right)\mathcal{=N}\left( x_{t-1};\mu_{\theta}\left( x_{t},t \right),\Sigma_{\theta}\left( x_{t},t \right) \right),$ (4)

where ​ $\mu_{\theta}$and $\Sigma_{\theta}$​ are the mean and covariance parameters predicted by the neural network with parameters $\theta$. The model is trained to minimize the variational bound on the negative log-likelihood, which can be expressed as:

${\mathcal{L}\mathbb{= E}}_{q}\left[ D_{KL}\left( q\left( x_{T}\mid x_{0} \right)\|p\left( x_{T} \right) \right)+\sum_{t=1}^{T} D_{KL}\left( q\left( x_{t-1}\mid x_{t},x_{0} \right)\|p_{\theta}\left( x_{t-1}\mid x_{t} \right) \right)-\log p_{\theta}\left( x_{0}\mid x_{1} \right) \right],$ (5)

Here, $D_{KL}$​ denotes the Kullback-Leibler divergence, and $p\left( x_{T} \right)$is typically chosen as a standard normal distribution. DPMs often integrate with models like VAE[9] and VQ-VAE[175] for latent space compression, enhancing their capacity to model complex data distributions. The reverse diffusion process is then guided by various conditions such as text prompts[51,180,181], images[56,182], or other modalities[183], making DPMs adaptable for diverse tasks. Additionally, diffusion probabilistic models can decouple image components by separating spatial features, improving their ability to learn detailed image characteristics and enhancing their effectiveness in medical image generation

In the context of medical imaging, DPMs have demonstrated exceptional performance across a variety of tasks, including denoising[184,185], super-resolution[186,187], image synthesis[49,188],reconstruction[189–191], and so on. For instance, the Denoising Diffusion Probabilistic Model (DDPM)[184] has been widely utilized for noise reduction in X-ray imaging, improving image clarity and aiding in the detection of small fractures or nodules. The Denoising Diffusion MRI (DDM2)[185] further advances MRI denoising, effectively addressing complex and spatially varying noise patterns. For super-resolution tasks, SR3[186] has been successfully applied to optical coherence tomography, enhancing low-resolution retinal images into high-resolution ones, which is crucial for early disease detection in ophthalmology. Similarly, DiffIR[187] employs diffusion techniques to enhance ultrasound images, providing high-resolution scans that reveal subtle pathological changes, such as small tumors. In 3D imaging, 3D-DDPM[188] has been used to generate detailed brain models from limited data, facilitating neurosurgical planning and decision-making. Additionally, DPMs, through their physical heuristic modeling of diffusion processes, have proven valuable in medical image reconstruction from a physics-based perspective. They are applied in low-dose CT/PET imaging[189,190] and undersampled MRI reconstruction[191], where they help to recover high-fidelity images from incomplete data.

Collectively, these advancements underscore the growing impact of DPMs in medical imaging[192], offering state-of-the-art solutions for denoising, resolution enhancement, and high-fidelity image synthesis. However, DPMs' multi-step inference process results in slower generation speeds and higher computational costs. Moreover, the latent noise inherent in the diffusion process complicates model interpretability compared to the more structured latent features in GANs. Nonetheless, DPMs remain a powerful tool in medical imaging, producing high-quality outputs with rich detail that hold immense potential for clinical applications. As a result, DPMs have become one of the most popular and cutting-edge generative techniques in image generation today.

- 1. **Sequence modeling architectures**

In medical image generative models, sequence modeling architectures are crucial for tasks involving temporal or sequential data, such as dynamic imaging, or longitudinal studies. Key models include Transformers[11], Mamba[12], and Autoregressive (AR) models[13,193]. Figure 2(d) presents their core structures, which will be discussed in the following sections.

- - 1. ***Transformer***

Transformers have revolutionized deep learning by capturing long-range dependencies via self-attention, allowing them to model global relationships within data, unlike traditional CNNs that focus on localized receptive fields. The self-attention mechanism computes a sequence's representation by relating different positions within it, using query (Q), key (K), and value (V) matrices. The attention scores are calculated by the dot product of Q and K, scaled by the square root of the dimension and passed through a softmax function:

$\mathrm{Attention}\left( Q,K,V \right)=\mathrm{softmax}\left( \frac{QK^{T}}{\sqrt{d_{k}}} \right)V,$ (6)

The attention mechanism in Transformers allows tokens to dynamically model relationships across entire sequences, making them particularly effective in medical imaging, where convolutional neural networks (CNNs) struggle with limited receptive fields. For instance, 3D MedDiffusion[49] integrates self-attention within diffusion steps to enhance chest CT synthesis, ensuring structural consistency across slices. However, the quadratic complexity O(L²) of standard Transformers poses scalability challenges for high-resolution 3D volumes. To overcome this, MedFormer[194] employs axial attention, significantly reducing computational costs while maintaining global inter-slice correlations. Similarly, hybrid architectures like SwinGAN[195] combine convolutional layers for local texture refinement with Swin Transformer blocks to capture global contextual information, improving MRI reconstruction performance. Beyond image synthesis, Transformers have become a cornerstone of generative AI in medical imaging due to their scalability and multi-modal processing capabilities. For example, ViT-GPT2[196] integrates a vision Transformer with a GPT-2 decoder to generate radiology reports from X-rays, while DALL-E in Medicine[197] adapts Transformer models to synthesize anatomically consistent X-rays from text descriptions, achieving high clinical relevance scores.

The success of Transformers is largely attributed to their architectural flexibility, where self-attention layers enable deep feature extraction and positional embeddings retain spatial and temporal relationships. However, their high computational demands remain a key limitation, particularly for real-time medical applications. To address this, more efficient alternatives like Mamba have been introduced, aiming to reduce computational complexity while retaining the core benefits of Transformer-based models in medical imaging.

- - 1. ***Mamba***

The Mamba architecture, built upon state space models (SSMs)[12], has emerged as a transformative framework for medical image synthesis, addressing critical limitations of conventional models like Transformers (quadratic complexity) and CNNs (local-receptive constraints)[26]. At its core, Mamba employs discretized state space equations to model sequential dependencies with linear computational scaling:

$h_{t}=\bar{A}_{t}h_{t-1}+\bar{B}_{t}x_{t},$ (7)

$y_{t}=\bar{C}_{t}h_{t},$ (8)

where $h_{t}$ denotes the hidden state, $x_{t}$is the input, and $\bar{A}_{t},\bar{B}_{t},\bar{C}_{t}$ ​are discretized parameters derived via zero-order hold (ZOH). This formulation enables efficient integration of long-range features while maintaining the fidelity of local details, which is essential for medical imaging applications.

To improve Mamba’s application in medical image classification, researchers introduced MedMamba[198], the first Vision Mamba model tailored for this task. Leveraging the efficiency of state-space models (SSMs), MedMamba aims to set a new benchmark in medical image classification. Meanwhile, the Vision Mamba Denoising Diffusion Probabilistic Model (VM-DDPM)[199] has shown exceptional performance in medical image synthesis, integrating CNN-based local feature extraction with SSM-driven global modeling, while maintaining linear computational complexity, making it well-suited for high-resolution imaging. Beyond classification and synthesis, Mamba-based models have proven valuable in radiotherapy planning. The MD-Dose model[61] employs Mamba encoders to efficiently propagate tumor-bed contextual information, significantly reducing errors in 3D dose maps. In video generation, VideoMamba[200] is optimized for long-video modeling, operating six times faster than TimeSformer[201] while efficiently adapting to multi-modal generative tasks. Additionally, MambaMixer[202] refines multi-dimensional data modeling, making it highly effective for multi-modal video generation.

These developments underscore Mamba’s capability to serve as an alternative to Transformers or complement them in resource-constrained environments. Its selective state transitions filter out irrelevant information while preserving critical anatomical dependencies. While Mamba offers efficient long-range sequence modeling, its limited pre-training ecosystem compared to Transformers constrains its application in multi-modal medical imaging. Additionally, the state compression mechanism, although computationally advantageous, may hinder the retention of fine-grained local information, potentially leading to memory dilution or information forgetting in complex clinical tasks.

- - 1. ***Autoregressive Models***

Autoregressive (AR) models[13,193] generate images sequentially, predicting each pixel (or voxel) based on the previously generated ones. This sequential dependency modeling has proven highly effective in medical image synthesis, particularly for tasks requiring fine-grained pixel-level detail. By factorizing the joint distribution p(x) of an image into a product of conditional probabilities, AR models ensure that each generated element maintains consistency with prior context.

$p\left( \mathbf{x} \right)=\prod_{t=1}^{T} p\left( x_{t}\mid x_{<t} \right),$ (9)

where $x_{t}$ represents the $t$-th element (e.g., pixel, patch, or token) in a predefined generation order, and $x_{<t}$ denotes all previously generated elements. For high-dimensional medical images, this sequential dependency is often modeled using neural networks, such as Transformers or CNNs, to parameterize $p\left( x_{t}\mid x_{<t} \right)$.

In longitudinal medical imaging, particularly for studying aging processes and disease progression, the Sequence-Aware Diffusion Model (SADM)[69] combines autoregressive (AR) models with diffusion processes to synthesize aging-aligned brain MRI sequences. For accelerated MRI reconstruction, the Autoregressive Image Diffusion (AID) model[203] enforces k-space consistency through retrospective sampling, suppressing aliasing artifacts compared to standard diffusion models. Additionally, recent advancements, such as MambaRoll[204] integrate AR with state-space model (SSM) latent states at the patch level, improving performance in medical image reconstruction while maintaining computational efficiency.

The "causal constraint" inherent in AR models makes them naturally suited for medical image synthesis, particularly when preserving anatomical continuity. However, these models often rely on external frameworks, such as Mamba or Transformer-based models[204], [69], to provide global priors in order to prevent error accumulation and ensure the accuracy of the synthesized data. This dependency highlights the importance of integrating both local and global contextual information for improving the performance and robustness of AR-based medical imaging applications.

Overall, Transformers effectively capture global dependencies through self-attention mechanisms but are limited by quadratic complexity, which hinders processing long sequences. Mamba addresses this limitation by utilizing a linear state-space model, trading some global awareness for computational efficiency. Autoregressive models excel at handling local dependencies but lack bidirectional context. The relationships among these approaches, as illustrated in Figure S2, highlight their complementary nature and suggest that integration can yield significant benefits. For instance, combining Transformers with state-space models[205] can reduce training time while preserving global context. Platforms like the MONAI Model Zoo[206] facilitate such integrations by providing pre-trained models, enabling rapid fine-tuning and advancing medical imaging towards more generalizable paradigms.


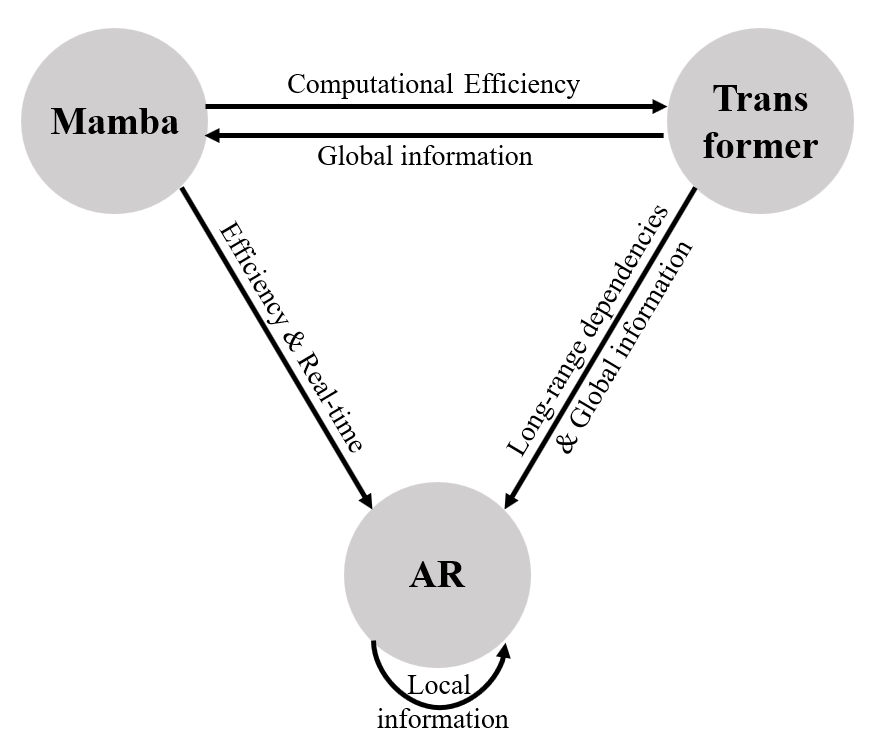


**Figure S2.** A relationship diagram illustrating the integration of Transformer, Mamba, and AR models

- 1. **Emerging trends of Multimodal** **Foundation Models**

With the growing demand for unified, scalable, and data-efficient solutions in medical imaging, foundation models have emerged as a promising paradigm. These models are typically pretrained on large-scale datasets and designed to generalize across tasks and modalities, often requiring minimal task-specific supervision. Building on the progress of earlier image-only generative models such as GANs and diffusion models, the field has increasingly shifted toward foundation models that integrate both visual and textual information[14–16,127,148]. Foundation models in medical imaging have evolved along several complementary directions, including vision-only models trained via self-supervised learning (e.g., masked autoencoder[207]); language-only models designed for medical report understanding or generation[208–210]; and vision-language models that learn jointly from paired image-text data, such as radiology images and associated reports[14,50,181].

In medical imaging, vision-language foundation models typically adopt a dual-encoder architecture that includes a visual encoder (e.g., convolutional neural networks or Vision Transformers) and a text encoder (e.g., transformer-based language models)[106,211,212]. These two modalities are jointly trained using contrastive learning to align their representations in a shared embedding space. The core idea is to bring matching image–text pairs closer together while pushing non-matching pairs apart. This framework forms the basis of many large-scale pretrained architectures as illustrated in Figure 2 (e), enabling models to generalize across tasks with limited supervision and to support applications such as zero-shot classification, report retrieval, and text-guided image synthesis. These models are trained with a variant of the InfoNCE loss:

$L_{\text{contrast }}=-\frac{1}{N}\sum_{i=1}^{N} \log\frac{\exp\left( \mathrm{sim}\left( f\left( I_{i} \right),g\left( T_{i} \right) \right)/\tau\right)}{\sum_{j=1}^{N} \exp\left( \mathrm{sim}\left( f\left( I_{i} \right),g\left( T_{j} \right) \right)/\tau\right)},$ (10)

where $f\left( I \right)$ and $g\left( T \right)$ are image and text encoders, sim(.) is a similarity (e.g., cosine), and $\tau$ is a temperature. This contrastive training ensures paired images and captions have high similarity, enabling zero-shot image classification and retrieval.

In the field of medical AI, foundation models are emerging as a transformative paradigm for building more generalizable and versatile systems. A representative example is CheXzero, which leverages contrastive learning on paired chest X-rays and free-text radiology reports to enable zero-shot multi-label classification of thoracic diseases. By aligning visual and textual modalities in a shared latent space, CheXzero[213] bypasses the need for explicit disease annotation and instead learns directly from natural language supervision. This approach highlights a key advantage of foundation models: the ability to scale learning through self-supervision on large, unstructured datasets. More broadly, such models pave the way toward Generalist Medical AI (GMAI)—systems trained across diverse modalities (imaging, text, clinical data)[214] to perform a wide range of tasks with minimal task-specific supervision. As a result, the development of large-scale, self-supervised, multimodal foundation models has become a central trend in medical AI research.

1. **More Details on Key Applications of Generative AI in Medical Imaging**
   1. **Acquisition and Reconstruction Phase: Enhancing Data Quality and Availability**
      1. ***Denoising and artifact removal***

In medical imaging, noise and artifacts are major obstacles in medical imaging, often obscuring critical anatomical details and reducing diagnostic reliability. As different modalities exhibit unique noise characteristics, generative AI models are increasingly tailored to address modality-specific degradation while aligning with clinical demands. Details are summarized in Table S2 on denoising and artifact removal.

In low-dose CT (LDCT), quantum noise and metal-induced streak artifacts remain major barriers to visualizing fine anatomical structures. Traditional denoising filters often failed to preserve structural details. To overcome this, unsupervised Poisson flow models have been introduced for photon-counting CT, effectively suppressing stochastic noise while preserving tissue contrast[27]. In dental imaging, cycle-free invertible architectures have shown superior performance in removing metal-induced beam-hardening artifacts, enhancing implant planning accuracy[28]. More recent frameworks, such as progressive Wasserstein GANs combined with residual encoder-decoder architectures, have demonstrated improved delineation of bronchial walls and subtle lesions[215]. For cardiac CT, TT U-Net[216] leveraged temporal transformers to reduce motion artifacts and enhance phase consistency by learning spatiotemporal features from pseudo all-phase dataset. Meanwhile, diffusion-based models like CoreDiff[29] simulated noise decay dynamics to reconstruct high-fidelity images under ultra-low-dose conditions, notably aiding in lung nodule detection. Further advancements such as DenoMamba[217] employed state-space modeling to capture short- and long-range dependencies for effective denoising while preserving diagnostic quality.

In PET imaging, statistical noise has interfered with the accurate quantification of radiotracer uptake, which is essential for tumor detection and assessment of therapeutic response. Earlier attempts to transfer CT-based denoising models to PET[218,219] suffered from poor adaptability to tracer dynamics. To address this limitation, parameter-transferred Wasserstein GANs have been developed to improve noise suppression while preserving quantitative radiotracer uptake[30]. More recently, diffusion-based models have been applied to PET denoising by treating noise as a stochastic process. For instance, denoising diffusion probabilistic models enhanced image quality and anatomical fidelity without compromising uptake accuracy[31] while the ControlNet-guided 3D diffusion model[220] enabled adaptive denoising of whole-body PET scans using low-dose inputs as conditional priors. These approaches improve the reconstruction of biologically realistic signal distributions, thereby reducing noise-related bias in quantitative analysis and enhancing the reliability of PET imaging for tumor evaluation.

In MRI, images are prone to Rician noise and motion artifacts, particularly in dynamic acquisitions. Conventional denoising often blurred structural boundaries. To combat this, a residual Wasserstein GAN has been employed to model inter-slice anatomical consistency, improving both detail preservation and contrast[32]. Complementarily, a content–noise complementary learning strategy enabled joint modeling of clean and noisy signal components, offering a more nuanced restoration[221]. In fetal MRI, a GAN-based motion correction method effectively reconstructed consistent anatomical structures from misaligned slices[222]. Recent developments have also explored diffusion-based models: regularized reverse diffusion improves denoising and super-resolution jointly[33], while alternate mask-guided diffusion in the pixel-frequency domain provided robust motion artifact removal without compromising structural fidelity[223]. Across imaging modalities, these models reflect a shift toward clinically grounded, structure-aware restoration that not only reduces noise but also preserves anatomical plausibility, which is essential for tasks such as lesion detection, segmentation, and treatment planning.

**Table S2**. Summary of publications on denoising and artifact removal.

| **Publication (Year)** | **Model** | **Application** | **Loss Function** | **Link** |
| --- | --- | --- | --- | --- |
| PWGAN-WSHL (2021) [215] | GAN | Low-dose CT denoising | WGAN loss, L1 loss, MSE loss, structural loss | – |
| DenoMamba (2024) [217] | Mamba | Low-dose CT denoising | L1 loss | [√](https://github.com/icon-lab/DenoMamba) |
| m-WGAN (2019) [28] | GAN | CT image artifact removal | WGAN loss, MSE loss | – |
| TT U-Net (2023) [216] | Transformer | CT image artifact removal | L1 loss, adversarial loss | [√](https://github.com/ivy9092111111/TT-U-Net) |
| CoreDiff (2024) [29] | Diffusion model | Low-dose CT denoising | Diffusion denoising loss | [√](https://github.com/qgao21/CoreDiff) |
| PFGM++ (2024) [27] | Diffusion model | Photon-counting CT denoising | Diffusion denoising loss | [√](https://github.com/Newbeeer/pfgmpp) |
| Yang et al. (2021) [219] | CNN | PET image denoising and artifact Removal | MSE loss | – |
| Hu et al. (2020) [218] | GAN | PET image denoising and artifact Removal | WGAN loss, MSE loss, gradient difference loss, content loss, ssim loss | – |
| PT-WGAN (2020) [30] | GAN | PET image denoising | Adversarial loss, MSE loss, ssim loss, perceptual loss | [√](https://github.com/90n9-yu/PT-WGAN) |
| Gong et al. (2024) [31] | Diffusion model | PET image denoising | Diffusion denoising loss | – |
| Yu et al. (2024) [220] | Diffusion model | PET image denoising | Diffusion denoising loss | – |
| RED-WGAN (2019) [32] | GAN | MRI image denoising | WGAN loss, MSE loss, perceptual loss, VGG loss | [√](https://github.com/Deep-Imaging-Group/RED-WGAN) |
| Chung et al. (2022)[29] | Diffusion model | MRI image denoising | Diffusion denoising loss | – |
| CNCL (2022) [221] | GAN | MR, CT and PET image denoising | Content loss, noise loss, GAN loss | [√](https://github.com/gengmufeng/CNCL-denoising) |
| Lim et al. (2023) [224] | GAN | MRI image artifact removal | WGAN loss, L1 loss, VGG loss | – |
| PFAD (2024) [225] | Diffusion model | MRI image artifact removal | Diffusion denoising loss | [√](https://github.com/medcx/PFAD) |

- - 1. ***Accelerated image reconstruction***

Generative models have significantly advanced medical image reconstruction, addressing limitations in image quality, sampling sparsity, and modality constraints. Across CT, PET, MRI, and emerging modalities, as summarized in Table S3**,** they enable high-fidelity recovery from low-dose or incomplete data, improving diagnostic accuracy and facilitating real-time clinical applications.

***CT Reconstruction****:* High-quality CT reconstruction is critical for accurate lesion detection and surgical planning, but dose reduction has introduced challenges such as projection sparsity and artifact amplification. Traditional algorithms often failed to meet clinical demands for detail preservation under low-dose protocols*.* To address these limitations, GAN-based sinogram restoration frameworks have been proposed, generating missing projection data with up to 95.88% SSIM under 60° scans for lung nodule screening[35]. Pix2pix GAN enabled cross-scanner kernel transfer, reducing quantification bias in emphysema studies and enhancing multicenter consistency[226]. In acute cases like intracranial hemorrhage DOLCE, a diffusion-based model, reconstructed high-fidelity images from limited-angle inputs while reducing metal artifacts[36]. For orthopedic applications, the conditional GAN synthesized 3D CT from single-view X-rays, aiding acetabular cup planning in THA[227], while neural radiance fields enabled detailed 3D knee modeling from 2D data[228] . Material decomposition in dual-energy CT has also benefited from 3D generative networks for accurate calcification assessment[229]. In trauma imaging, Mamba-based Monte Carlo frameworks drastically reduced reconstruction time while maintaining quality[230]. Recent diffusion models have improved sparse-view consistency and accelerated convergence[231,232] , and SWORD enhanced textural details[189]. Despite these advances, challenges remain in balancing artifact suppression, low-dose sensitivity, device generalizability, and real-time feasibility for clinical deployment.

***PET Reconstruction****:* Positron emission tomography (PET) plays a unique role in assessing tumor metabolism, diagnosing neurodegenerative diseases, and monitoring treatment. However, its clinical application has been constrained by issues such as low signal-to-noise ratio, multimodal registration errors, and the complexity of dynamic imaging. Generative approaches helped address these barriers across reconstruction, attenuation correction, and personalization. For low-count PET, CycleGAN-based model maintained tumor metabolic volume consistency in pediatric oncology[37] with the normalized correlation coefficient improving from 0.970 to 0.996. Similarly, the method[233] developed a noise-aware adaptive loss function to balance noise distribution and anatomical fidelity in low-count data, enhancing the stability of the striatal dopamine transporter binding ratio in Parkinson’s disease diagnosis. To reduce attenuation correction bias in PET/MRI, a joint reconstruction framework[234] simultaneously estimated activity and attenuation maps with <1% SUV error. Bimodal VAEs further reduced PET–MRI registration error by decoupling modality-specific features[38]. Dynamic PET benefited from deep generalized learning that restores fine details from sparse temporal frames[39]. In personalized therapy, a projection-domain CNN estimated individualized dose distributions for ^177^Lu treatment[235]. Lightweight architectures like Cycle-PET achieved sub-second reconstruction on embedded GPUs for emergency use[236]. And a projection generative network trained on Monte Carlo simulated data to reduce acquisition time without sacrificing tumor-to-background contrast[237]. Addition, studies[238,239] and more recent works[190,230,240,241] have further extended PET reconstruction techniques through task optimization, image synthesis, and diffusion modeling, providing stronger technical support for precision clinical diagnosis and treatment.

***MRI Reconstruction****:* The quality of MRI reconstruction critically affects early disease detection, diagnostic precision, and treatment planning. Yet, conventional methods remained limited by noise, motion artifacts, and data inconsistency, which hindered accurate visualization of small lesions and subtle anatomical details. In neuroimaging, where structural clarity is essential, recent generative approaches have markedly improved reconstruction quality[242,243]. In pediatric imaging, where reducing scan time is essential, GAN-based approaches integrated with compressed sensing have shown promise in generating high-resolution images with superior lesion contrast and improved edge definition[238,244,245]. Building on this, a transformer-based model using global self-attention has better captured multi-scale features, strengthening both qualitative and quantitative brain assessments[40]. For cardiac MRI, the dynamic nature of the heart demands real-time reconstruction and artifact suppression. Diffusion and score-based generative models have shown strong performance in restoring temporal details and reducing motion-induced distortions, even under sparse sampling[41,191,246,247]. Additionally, the Mamba framework, which incorporates uncertainty quantification, provided valuable insights into reconstruction reliability and thereby supported risk-aware clinical decision-making[42,248]. To improve consistency in low-field MRI and multicenter studies, where acquisition parameters and device variability are common, researchers have explored federated learning, regularization, and meta-learning hypernetworks[249–252]. These approaches not only ensured the recovery of image details under low-field conditions but also facilitated the integration of multicenter data, thus contributing to more consistent diagnostic outcomes in large-scale clinical studies[253]. Notably, the generative autoregressive transformer proposed in the work[254] enabled model-agnostic, privacy-preserving MRI reconstruction by capturing complex spatial-temporal patterns in distributed datasets. MambaRoll[204] combined autoregressive mechanisms with state space representations at the patch level, enabling efficient and high-quality reconstruction. AID[203] further explored sequential image generation for reconstructing MRI scans, offering improved spatial coherence and progressive refinement. These approaches have demonstrated the effectiveness of autoregressive models in capturing structured dependencies for medical image reconstruction. In summary, advanced MRI reconstruction techniques have demonstrated distinct advantages across neuroimaging, cardiac imaging, and low-field/multicenter applications. The future challenge lies in seamlessly integrating these advanced methods into clinical workflows to achieve real-time, efficient reconstruction and to validate their performance on large-scale, multicenter datasets.

***Others:*** Beyond conventional CT, PET, and MRI, generative models are increasingly been applied in ultrasound, photoacoustic, and EEG-based imaging, expanding the landscape of image reconstruction. In ultrasound, fast-sampling generative models enabled high-quality reconstruction with reduced data requirements, supporting real-time clinical applications[255] . Diffusion-based methods incorporated uncertainty quantification and variance modeling, improving structural fidelity and diagnostic confidence[256,257]. For single plane-wave data, generative models enhanced signal-to-noise ratio and contrast, boosting sensitivity in early lesion detection[258]. In photoacoustic imaging, reconstruction has been particularly challenging due to data sparsity and limited sampling angles. By combining diffusion models with iterative optimization[259], it has mitigated artifacts and loss of detail, while score-based generative models with rotational consistency constraints[260] ensured image consistency across different angles, providing reliable support for tumor and tissue function assessment. Moreover, the DM-RE2I framework[261] leveraged diffusion models to map EEG signals into image space, exploring the conversion from neural electrical activity to structural images and opening new avenues for early diagnosis and functional localization of neurological disorders. Taken together, generative models are reshaping image reconstruction across both established and emerging modalities. By enhancing image detail, reducing artifacts, and incorporating uncertainty modeling, these methods significantly improve diagnostic reliability. As diffusion and hybrid frameworks continue to evolve, generative reconstruction is poised to play an increasingly central role in precision diagnostics and personalized care.

**Table S3.** Summary of publications on medical image reconstruction.

| **Publication (Year)** | **Model** | **Application** | **Loss Function** | **Link** |
| --- | --- | --- | --- | --- |
| **CT Reconstruction** | |  |  |  |
| DL-recon (2022) [229] | GAN | CBCT-to-CT reconstruction | Adversarial loss, L1 loss | – |
| Pradhan et al. (2023) [227] | GAN | 2D-to-3D CT reconstruction | L1 loss, BCE loss, adversarial loss | – |
| HyperNeRFGAN (2024) [228] | GAN | X-ray-to-CT reconstruction | StyleGAN2Loss | [√](https://github.com/gmum/HyperNeRFGAN) |
| Krishnan et al. (2024) [226] | GAN | Low-dose CT reconstruction | L1 loss, adversarial loss, reconstruction loss | [√](https://github.com/MASILab/KernelConversionIntraVender) |
| MambaMIR (2025) [230] | GAN, Mamba | Low-dose CT/PET reconstruction | Adversarial loss, Charbonnier loss，image loss, frequency loss | – |
| SI-GAN (2019) [35] | GAN | Limited-angle CT reconstruction | Adversarialloss, sinogram loss, reconstruction loss | – |
| DOLCE (2023) [36] | Diffusion model | Limited-angle CT reconstruction | Diffusion denoising loss | [√](https://github.com/wustl-cig/DOLCE) |
| TIFA (2024) [232] | Diffusion model | Limited-angle CT reconstruction | Diffusion denoising loss | [√](https://github.com/tianzhijiaoziA/TIFADiffusion) |
| CDDM (2024) [231] | Diffusion model | Sparse-view CT reconstruction | Diffusion denoising loss | – |
| SWORD (2024) [189] | Diffusion model | Sparse-view CT reconstruction | Diffusion denoising loss | [√](https://github.com/yqx7150/SWORD) |
| **PET Reconstruction** | |  |  |  |
| NADRU (2020) [233] | CNN | Low dose PET reconstruction | Dice loss, BCE loss, general and adaptive robust loss, ssim loss | – |
| Shi et al. (2023) [234] | CNN | Low dose PET reconstruction | L1 loss, image domain loss, gradient difference loss, LIP loss | [√](https://github.com/j-onofrey/deep-image-pet) |
| CPR-CNN (2024) [236] | CNN | Low dose PET reconstruction | Reconstruction loss, cycle consistency loss | – |
| DGLM (2024) [39] | CNN | Low count PET reconstruction | MSE loss, ssim loss | – |
| Lei et al. (2019) [37] | GAN | Low count PET reconstruction | Cycle-consistent adversarial loss, gradient descent loss, mean p-norm distance loss | – |
| Task-GAN (2019)[238] | GAN | Ultra-low dose PET reconstruction | L1 loss, adversarial loss, regression loss | – |
| AR-GAN (2022) [239] | GAN | Low dose PET reconstruction | L1 loss, adversarial loss, cross-entropy loss | – |
| DDPET-3D (2024) [241] | Diffusion model | Low dose PET reconstruction | Diffusion denoising loss | – |
| Wikberg et al. (2024) [235] | CNN | Sparsely acquired projections PET reconstruction | L1 loss, MSE loss | – |
| MMJSD (2024) [38] | VAE | Bimodal PET/MRI reconstruction | Negative log-likelihood loss, KL loss | – |
| Singh et al. (2024) [240] | Diffusion model | 2D/3D PET reconstruction | Poisson Log-Likelihood loss, Diffusion denoising loss | [√](https://github.com/Imraj-Singh/Score-Based-Generative-Models-for-PET-Image-Reconstruction) |
| MC-Diffusion (2024) [190] | Diffusion model | PET-MRI reconstruction | Diffusion denoising loss | [√](https://github.com/taofengxie/PET-MRI-reconstruction) |
| **MRI reconstruction** | |  |  |  |
| Wang et al. (2019) [244] | GAN | MRI reconstruction | Content loss, perceptual loss, adversarial loss, dc loss | – |
| rsGAN (2020) [245] | GAN | Multi-contrast MRI reconstruction | L1 loss, perceptual loss, adversarial loss, dc loss | – |
| Kelkar et al. (2021) [250] | GAN | MRI reconstruction | MSE loss, log-likelihood loss, TV loss, dc loss | – |
| SwinMR (2022) [40] | Transformer | MRI reconstruction | Pixel-wise Charbonnier loss, frequency Charbonnier loss | [√](https://github.com/ayanglab/SwinMR) |
| KM-MAML (2023) [252] | CNN | MRI reconstruction | L1 reconstruction loss, dc loss | [√](https://github.com/sriprabhar/KM-MAML/) |
| MambaMIR (2024) [42] | Mamba | MRI reconstruction | Adversarial loss, image loss, kspace loss, perceptual loss, dc loss | [√](https://github.com/ayanglab/MambaMIR) |
| DM-Mamba (2025)[248] | Mamba | MRI reconstruction | L1 loss, dc loss | [√](https://github.com/XiaoMengLiLiLi/DM-Mamba) |
| MambaRoll (2024) [204] | Mamba, AR | MRI reconstruction | Kspace loss, cascade loss, dc loss | [√](https://github.com/icon-lab/MambaRoll) |
| HFS-SDE (2024) [247] | Diffusion model | MRI reconstruction | Diffusion denoising loss, dc loss | [√](https://github.com/Aboriginer/HFS-SDE) |
| JSMoCo (2025) [41] | Diffusion model | MRI reconstruction | Diffusion denoising loss, dc loss | [√](https://github.com/MeijiTian/JSMoCo) |
| AID (2025) [203] | Diffusion model, AR | MRI reconstruction | Diffusion denoising loss, dc loss | [√](https://github.com/mrirecon/aid) |
| Kofler et al. (2020) [249] | CNN | Cardiac cine MRI reconstruction | L2 loss, dc loss | – |
| Qiu et al. (2024) [191] | Diffusion model | Cardiac cine MRI reconstruction | Diffusion denoising loss, dc loss | – |
| DiffCMR (2024) [262] | Diffusion model | Cardiac cine MRI reconstruction | Diffusion denoising loss, dc loss | [√](https://github.com/xmed-lab/DiffCMR) |
| FedGIMP (2023) [251] | GAN | Federated MRI reconstruction | Logistic adversarial loss, local reconstruction loss, dc loss | [√](https://github.com/icon-lab/FedGIMP) |
| FedGAT (2025) [254] | VAE, Transformer, AR | Federated MRI reconstruction | Perceptual loss, adversarial loss, cross-entropy loss, MSE loss, dc loss | [√](https://github.com/icon-lab/FedGAT) |
| **Otherrs** |  |  |  |  |
| DDRM (2023) [256] | Diffusion model | US image reconstruction | Diffusion denoising loss | [√](https://github.com/openai/guided-diffusion) |
| DRUSvar (2024) [257] | Diffusion model | US image reconstruction | Diffusion denoising loss | [√](https://github.com/Yuxin-Zhang-Jasmine/DRUSvar) |
| Lan et al. (2023) [255] | Diffusion model, GAN | US image reconstruction | Diffusion denoising loss | – |
| Merino et al. (2024) [258] | Diffusion model, GAN | US image reconstruction | Adversarial loss, Diffusion denoising loss | – |
| DM-RE2I (2023) [261] | Diffusion model | EEG to image reconstruction | Diffusion denoising loss | – |
| PAT-Diffusion (2023) [259] | Diffusion model | Photoacoustic tomography reconstruction | Diffusion denoising loss | [√](https://github.com/yqx7150/PAT-Diffusion) |
| Tong et al. (2023) [260] | Diffusion model | Photoacoustic tomography reconstruction | Diffusion denoising loss | – |

- - 1. ***Super-resolution***

Medical image resolution is often constrained by acquisition time, radiation dose, and physical limitations of imaging systems. Super-resolution (SR) methods (see Table S4) seek to reconstruct high-resolution images from low-quality inputs, and are commonly divided into temporal SR (for dynamic imaging) and spatial SR (for static structural enhancement) [263,264].

***Temporal super‐resolution****:* In dynamic imaging, temporal super‐resolution increases frame rates and enhances temporal consistency to capture continuous organ motion and suppress motion artifacts. High frame rate imaging enables more accurate functional assessments for rapidly moving organs such as the heart and lungs, aiding early disease diagnosis and treatment planning. Early efforts relied heavily on GAN-based frameworks with perceptual loss functions [265], which aimed to reconstruct intermediate frames by enhancing temporal fidelity and visual realism. These approaches were followed by optical flow‐guided models[266,267], that introduced motion-aware learning mechanisms to better capture video inter-frame dependencies. Building upon these foundations, more advanced architectures have explored spatial‐temporal interpolation. A deformation-based method enabled smooth motion transitions in 4D cardiac MRI by modeling local anatomical deformation[268], while the multi-pyramid voxel flow[269] improved interpolation performance under sparse temporal sampling. Subsequently, diffusion-based deformation models[43] offered more stable and noise-resilient frame generation, particularly useful in scenarios with irregular breathing or arrhythmias. Recent approaches such as the data-efficient interpolation network[270] and the dynamic dual-channel architecture[271] have further advanced temporal super-resolution by enhancing frame detail while minimizing artifacts. Besides, a diffusion-driven framework further enhanced temporal super-resolution and spatial consistency in 4D MRI imaging[46]. Meanwhile, hybrid models incorporating multi-level feedback loops and task-specific motion correction[266,272] have demonstrated strong generalization in clinical tasks such as myocardial ischemia evaluation, arrhythmia monitoring, and tumor motion tracking.

***Spatial super‐resolution****:* Spatial super-resolution (SR) techniques aim to reconstruct high-resolution medical images from low-resolution inputs, improving the visibility of fine anatomical details crucial for lesion detection, tissue boundary delineation, and vascular assessment. Traditional interpolation methods are limited in restoring texture and structural accuracy. Recent deep learning approaches have addressed these limitations through multimodal fusion, frequency-domain modeling, and generative modeling. Among early methods, GAN-CIRCLE[44] integrated identical, residual, and cycle consistency constraints within a GAN framework to improve structural fidelity in CT images. In MRI, SOUP-GAN[273] enhanced perceptual quality and reduces aliasing by learning texture-aware representations. Combining GANs with discrete wavelet transforms further improved texture realism and suppressed noise, enabling efficient SR in portable applications[274]. In X-ray imaging, frequency domain constraints have been shown to improve edge detail and suppress artifacts[275]. More recent methods leveraged hierarchical and diffusion-based models. Hierarchical amortized GAN allowed memory-efficient synthesis of high-resolution 3D medical images by capturing both global and local features[276], while local-to-global feature learning frameworks further enhanced anatomical consistency across scales[277]. Diffusion models have also been adopted: UHRCT_SR[45] employed a dual-stream structure-preserving network and an imaging enhancement operator for CT super-resolution, and partial diffusion model[278] accelerated the process by focusing on relevant components in brain MR images. In addition, the Deform-Mamba network [279] integrated deformable convolutions with state-space modeling to reconstruct high-quality MR images under limited resolution conditions. These methods reflect the ongoing shift from basic interpolation toward structurally informed reconstruction techniques. While notable improvements in image quality have been achieved, challenges such as modality variability, computational efficiency, and clinical integration remain areas of active research.

**Table S4.** Summary of publications on super-resolution.

| **Publication (Year)** | **Model** | **Application** | **Loss Function** | **Link** |
| --- | --- | --- | --- | --- |
| **Temporal super‐resolution** | |  |  |  |
| Ren et al. (2021) [266] | CNN | Video super‐resolution | L1 loss | – |
| Song (2022) [267] | CNN, Transformer | Video super‐resolution | MSE loss | – |
| VSRResFeatGAN (2019) [265] | GAN | Video super‐resolution | Adversarial loss, perceptual loss, charbonnier loss, | – |
| MFIN (2019)[272] | CNN | 4D MRI Temporal super‐resolution | Cycle consistency loss, recon loss, ssim loss, | – |
| SVIN (2020) [268] | CNN | 4D MRI Temporal super‐resolution | Similarity loss, smoothness regularization loss, regression loss | [√](https://github.com/guoyu-niubility/SVIN) |
| DDoS-Unet (2024) [271] | CNN | 4D MRI Temporal super‐resolution | Perceptual loss, L1 loss | [√](https://github.com/soumickmj/DDoS) |
| MPVF (2023) [269] | CNN, Transformer | 4D MRI Temporal super‐resolution | Charbonnier loss | [√](https://github.com/Tzu-Ti/MPVF) |
| UVI-Net (2024) [270] | CNN, Transformer | 4D MRI Temporal super‐resolution | NCC loss, gradient loss | [√](https://github.com/jungeun122333/UVI-Net) |
| DDM (2022) [43] | Diffusion model | 4D MRI Temporal super‐resolution | Diffusion denoising loss, NCC loss, KL loss | [√](https://github.com/torchDDM/DDM) |
| TSSC-Net | Diffusion model | 4D MRI Temporal super‐resolution | Diffusion denoising loss, NCC loss, KL loss | [√](https://github.com/Joker-ZXR/TSSC-Net) |
| **Spatial super‐resolution** | |  |  |  |
| GAN-CIRCLE (2019) [44] | GAN | CT super‐resolution | Adversarial loss, cycle-consistency loss, identity loss, joint sparsifying transform loss | [√](https://github.com/charlesyou999648/GAN-CIRCLE) |
| TTSR-FD (2021) [275] | GAN | X-ray super‐resolution | Frequency domain loss, perpetual loss, adversarial loss, | – |
| SOUP-GAN (2022) [273] | GAN | MRI super‐resolution | Adversarial loss, perceptual loss | [√](https://github.com/Mayo-Radiology-Informatics-Lab/SOUP-GAN) |
| DWT-SRGAN (2022) [274] | GAN | MRI super‐resolution | Perpetual loss, adversarial loss, wavelet loss | – |
| HA-GAN (2022) [276] | GAN | CT/MRI super‐resolution | GAN loss, reconstruction loss | – |
| Huang et al. (2024) [277] | Transformer, CNN, | US/OCT/Endoscope/CT/MRI super‐resolution | Charbonnier loss, L1 loss | – |
| UHRCT_SR (2023) [45] | Diffusion model | CT super‐resolution | Diffusion denoising loss | [√](https://github.com/Arturia-Pendragon-Iris/UHRCT_SR) |
| PartDiff (2023) [278] | Diffusion model | MRI super‐resolution | Diffusion denoising loss | – |
| Deform-Mamba (2024) [279] | Mamba | MRI super‐resolution | L1 loss, CE loss | – |

- 1. **Diagnosis Phase: Enriching Diagnostic Imaging**
     1. ***Unconditional synthesis***

Unconditional synthesis generates medical images from random noise or data distributions without specific constraints, enabling the creation of diverse and realistic samples without relying on annotated data. Early work in this area focused on generative adversarial networks (GANs), which produced synthetic images that resembled clinical data. Classical applications included the synthesis of blood vessel surfaces [197] and 3D brain MRI volumes[280], demonstrating GANs’ capacity to model complex anatomical structures without supervision. However, classical GANs struggled with limited controllability, mode collapse, and low spatial resolution, which limited their use in anatomically precise tasks. To overcome these limitations, more advanced architectures such as StyleGAN[47,281] introduced structured latent spaces and style-based modulation, enabling fine-grained control over image attributes and significantly improving visual realism and resolution.

Driven by the need for higher fidelity and diversity, diffusion models have emerged as a robust alternative to GANs for unconditional medical image generation. Unlike GANs' single-shot generation, diffusion models employ a multi-step denoising process to iteratively transform random noise into structured images, offering greater training stability and output diversity. A denoising diffusion probabilistic model has shown strong performance in 3D medical imaging, particularly in enhancing clarity and resolution in volumetric CT and MRI data[122]. It improved downstream segmentation dice from 0.91 to 0.95 by generating large-scale synthetic data for self-supervised pretraining. Model likes 3D MedDiffusion[49] further supported high-resolution, anatomically specific image synthesis for segmentation enhancement, achieving a 6.14% dice improvement and 20.66 mm reduction in 95HD for tumor segmentation when training data were limited. Moreover, diffusion models are well-suited for handling multimodal variability, improving robustness under challenging imaging conditions like noise and contrast shifts[282,283]. To further enhance visual fidelity and structural consistency, specialized variants have been developed. Some focused on CT-specific improvements[284], while others integrated frequency or attention-based priors to improve anatomical realism[199]. And the Deformation-Recovery Diffusion Model[285] introduced spatial controllability via anatomically plausible deformation fields, improving segmentation and registration tasks. In cardiac MRI, DRDM achieved an average dice of 88.6% and sensitivity of 94.9%, outperforming conventional augmentation methods. These developments have highlighted the multifunctionality of diffusion models in generating data for a wide range of clinical applications[283].

In summary, unconditional image synthesis, especially using GANs and diffusion models, has shown considerable potential in producing diverse and high-quality medical images without the need for annotated datasets, as summarized in Table S5. These methods have proven instrumental in addressing data scarcity, improving the generalizability of diagnostic algorithms, and supporting downstream tasks such as early disease detection and treatment planning. However, their lack of explicit control over generated content can limit clinical utility in scenarios requiring precise anatomical, pathological, or modality-specific constraints. This has led to increasing interest in conditional synthesis approaches, which incorporate prior knowledge such as textual descriptions, anatomical maps, or clinical parameters to guide image generation toward specific diagnostic or therapeutic objectives.

**Table S5.** Summary of publications on medical image unconditional synthesis.

| **Publication (Year)** | **Model** | **Application** | **Loss Function** | **Link** |
| --- | --- | --- | --- | --- |
| Danu et al. (2019) [286] | VAE，GAN | Blood vessel surfaces synthesis | MSE loss，adversarial loss | – |
| Syn-Net (2020) [47] | GAN | 2D brain MRI synthesis | L1 loss, perceptual loss, adversarial loss | – |
| Chong and Ho (2021) [280] | GAN | 3D brain MRI synthesis | Adversarial loss，GAN loss | – |
| 3D-StyleGAN (2021) [281] | GAN | 3D MRI synthesis | MSE loss, Logistic loss | [√](https://github.com/sh4174/3DStyleGAN) |
| Txurio et al. (2023) [282] | Diffusion model | 2D CT synthesis | Diffusion denoising loss | – |
| MRGen (2024) [283] | Diffusion model， VAE | 2D MRI synthesis | Diffusion denoising loss | [√](https://github.com/haoningwu3639/MRGen) |
| VM-DDPM (2024) [199] | Diffusion model, Mamba | 2D X-ray/MRI synthesis | Diffusion denoising loss, GAN loss, BCE Loss | – |
| GH-DDM (2023) [284] | Diffusion model | 2D X-ray/CT/MRI/OCT synthesis | Diffusion denoising loss | – |
| Medicaldiffusion (2023) [122] | Diffusion model | 3D CT/MRI synthesis | Diffusion denoising loss | [√](https://github.com/FirasGit/medicaldiffusion) |
| DRDM (2024) [285] | Diffusion model | 3D CT/MRI synthesis | Distance error loss, angle error loss, regularization loss | [√](https://jianqingzheng.github.io/def_diff_rec/) |
| 3D MedDiffusion (2024) [49] | Diffusion model | 3D CT/MRI synthesis | Vector quantization loss, adversarial loss, tri-plane loss, Diffusion denoising loss | [√](https://github.com/ShanghaiTech-IMPACT/3D-MedDiffusion) |

- - 1. ***Conditional synthesis***

In contrast to unconditional synthesis, which learns image distributions independently of external inputs, conditional synthesis incorporates domain-specific priors such as clinical text, imaging data, anatomical structures, or physiological parameters into the generative process. This improves the relevance, controllability, and diagnostic value of the synthesized outputs. Conditional methods (see Table S6) can be broadly categorized into three types: text-to-image synthesis, image-to-image translation and completion, anatomically guided synthesis. Each reflects an evolving effort to bridge data-driven generation with clinical context, enhancing both interpretability and utility.

***Text-to-Image Synthesis****：* In recent years, text-to-image synthesis has become an important direction in image generation, supported by the rapid development of latent diffusion models and their ability to integrate context. Initial efforts, such as AttnGAN[287], Mirrorgan[288], StackGAN[289], and Cogview[290], laid the groundwork by mapping simple textual inputs to image content. More recent models like DALL-E[291], Imagen[292], and Stable Diffusion[293] have significantly improved semantic consistency and resolution. In the medical domain, this modality facilitates the transformation of both free-text radiology reports and structured clinical variables such as age, sex, smoking history, blood pressure, and imaging modality into more diverse and representative medical images. Early efforts primarily focused on report-to-image synthesis, where structured or semi-structured radiology reports were used to condition chest x-ray generation. Models like Chest-diffusion[50] and Diff-CXR[181] translated radiology reports into synthetic chest X-rays while embedding disease priors, improving data diversity and interpretability. Chest-Diffusion reached an FID of 24.456 at 118.918 GFLOPs, and Diff-CXR improved FID/mAUC by 33.4%/8.0% (MIMIC-CXR) and 23.8%/56.4% (IU-Xray), achieving near full-data mAUC with only 1% real data augmented by synthetic samples. Building on this foundation, recent methods have expanded to include text-driven generation across multiple imaging modalities. For example, MediSyn[51] proposed a generalist framework for synthesizing a wide range of medical images, while TextoMorph[294] targeted tumor synthesis conditioned on lesion type and location. In the domain of CT generation, models such as GenerateCT[180], MAISI[295], MedSyn[296] enabled the generation of 3D chest CT volumes guided by textual descriptions, capturing both anatomical fidelity and modality-specific characteristics. Beyond free-text reports, some models adopted parametric conditioning using explicit clinical attributes. Cheart[297] generated cardiac anatomy (mean dice = 0.713, HD = 10.940 mm) from age, gender, body weight, and blood pressure, while EchoDiffusion[63] raised LVEF regression from R² 56% to 59% by rebalancing training data with 50% synthetic samples. Similarly, the HeartBeat model[64] produced echocardiography videos from multimodal physiological signals, aiding cardiac function assessment, with FID/FVD scores of 25.23/6.08 (A2C) and 31.99/9.96 (A4C). In neuroimaging, TaDiff[23] generates personalized longitudinal brain MRIs conditioned on treatment, with average SSIM 0.919 and downstream DSCs of 0.719 (future tumor prediction) and 0.849 (source tumor segmentation), showing treatment-dependent variations. Subsequent work[128] synthesized multimodal MRIs using acquisition parameters (TR, TE, TI) together with demographic factors including age, sex, and clinical condition. These developments reflect a shift from descriptive to clinically informed synthesis. Embedding structured clinical information into textual prompts enhances the control and realism of generated images while improving their alignment with diagnostic and prognostic workflows.

***Image-to-Image Synthesis****:* Image-to-image synthesis encompasses both modality translation and modality completion, offering practical solutions to missing or degraded imaging data. Unlike text-based methods, these approaches preserve spatial information and structural alignment, making them highly suitable for multimodal fusion. Modality translation transforms one imaging modality into another (e.g., MRI to CT), compensating for unavailable or low-quality scans[52]. Early models employed conditional GANs (cGANs)[298–300], achieving reasonable pixel-wise translations between CT, PET, and MRI. Later, unsupervised frameworks such as CycleGAN[53,54] addressed the lack of paired data by learning bidirectional mappings. Recent advances have incorporated sequential modeling (e.g., Mamba-enhanced transformers[301–304]) and diffusion probabilistic models (DPMs) [29,56,224] which offer improved structural preservation and generative flexibility. Conversely, modality completion seeks to recover missing image regions or entire modalities. AutoSyncoder[225] introduced an efficient multiresolution encoder–decoder for cross-modal MRI inference, achieving PSNR 29 to 30.5 dB and SSIM > 0.88 on BraTS-15. ResViT[55] further improved multi-contrast synthesis, outperforming convolutional, attention, and transformer baselines by up to 1.89 dB PSNR and 3.20% SSIM, producing fewer artifacts and clearer structures. Subsequent work advanced multimodal fusion, with transformer-based design [305] exploiting multi-scale integration and hybrid methods[306,307] combined pseudo-modalities (e.g., synthetic T2-weighted MRI) with coarse-to-fine refinement, enhancing robustness. Building on prior multimodal advances, frequency-guided diffusion models[308] and unified multimodal synthesis frameworks[309] achieved higher generation fidelity and anatomical coherence. FgC2F-UDiff achieved seven SOTA methods on BraTS and IXI with gains up to 4.23 dB PSNR and 3.59 dB over pix2pix, and the unified model produced high-fidelity multi-sequence MRIs (e.g., T1+T2→PD) with PSNR 31.86 dB and SSIM 0.980. Additional studies[303,310] integrated conditional generation and cross-dimensional knowledge guidance, advancing the field from single-modality synthesis to multimodal unified modeling. Overall, the evolution from GAN-based methods to transformer-diffusion hybrids illustrates a growing emphasis on cross-modal alignment, data efficiency, and spatial consistency in clinical image synthesis.

***Anatomically-Guided*** ***Image Synthesis****:* Anatomically guided image synthesis integrates structural priors—such as segmentation masks, tissue boundaries, or lesion annotations—into generative models to improve anatomical consistency and clinical reliability in synthetic images. By embedding explicit spatial information, these methods aim to generate outputs that closely reflect real anatomical structures and pathological variations. Early work such as the CG-SAMR network [311] demonstrated the effectiveness of incorporating lesion and tissue confidence into the synthesis of multi-contrast MR images, producing anatomically faithful outputs across different imaging modalities and achieving a 17.7% improvement in pixel accuracy over CycleGAN. Building on this, a work[312] introduced attribute disentanglement to control nodule shape, size, and texture in chest X-rays, and its HEM-based augmentation increased Faster R-CNN AUC from 0.9090 to 0.9385, confirming effective lesion-level data expansion. In the ophthalmic domain, synthesis methods have further expanded structural guidance. A two-stage model combining StyleGAN and GauGAN[313] generated diabetic fundus images from semantic lesion maps, while the vascular-guided GAN[57] leveraged vessel structure to preserve fine anatomical details in super-resolved retinal images. Beyond major organs, anatomical priors have also enabled synthesis in low-data or small-structure scenarios. LN-Gen[314] generated rectal lymph nodes by learning from anatomical features and shape representations, thereby improving U-Net segmentation performance (dice: 50.49% → 56.14%; sensitivity: 48.07% → 53.01%) and supporting data expansion in pelvic imaging. Similarly, SegGuidedDiff[58] conditioned the generation process on multi-class segmentation masks, achieving anatomically controllable synthesis on Breast MRI (dice = 0.9027) and demonstrating superior mask fidelity and downstream segmentation consistency over ControlNet and SPADE. Together, these methods highlight the growing role of anatomical priors in enhancing structural accuracy, interpretability, and the practical value of synthetic data in medical imaging.

**Table S6.** Summary of publications on medical image conditional synthesis.

| **Publication (Year)** | **Model** | **Application** | **Loss Function** | **Link** |
| --- | --- | --- | --- | --- |
| **Text-to-Image Synthesis** | |  |  |  |
| Campello et al. (2022) [75] | GAN | Clinical information-to-MRI | Adversarial loss, cycle-consistency loss | [√](https://github.com/vicmancr/CardiacAging) |
| CHeart (2023) [297] | VAE | Clinical information-to-MRI | KL loss, log-likelihood loss | [√](https://github.com/MengyunQ/CHeart) |
| TUMSyn (2025) [128] | Transformer, CNN | Clinical information-to-MRI | Contrastive loss, similarity loss | [√](https://github.com/Wangyulin-user/TUMSyn) |
| Del Castillo et al. (2025) [315] | Diffusion model, VAE | Clinical information-to-MRI | Diffusion denoising loss | – |
| TaDiff (2025) [23] | Diffusion model | Clinical information-to-MRI | Diffusion denoising loss，dice loss | – |
| MAISI (2024) [295] | Diffusion model | Clinical information-to-CT | Diffusion denoising loss | [√](https://github.com/Project-MONAI/tutorials/tree/main/generation/maisi) |
| EchoDiffusion (2023) [63] | Diffusion model | Clinical information-to-video | Diffusion denoising loss | [√](https://github.com/HReynaud/EchoDiffusion) |
| GenerateCT (2024) [180] | Diffusion model，Transformer | Report-to-chest CT synthesis | Diffusion denoising loss, perceptual loss, adversarial loss | [√](https://github.com/ibrahimethemhamamci/GenerateCT) |
| MedSyn (2024) [296] | Diffusion model, VAE | Report-to-chest CT synthesis | Diffusion denoising loss, KL loss | [√](https://github.com/batmanlab/MedSyn) |
| MediSyn (2025) [51] | Diffusion model, VAE | Text-guided diverse synthesis | Diffusion denoising loss | – |
| TextoMorph (2024) [294] | Diffusion model | Text-guided tumor synthesis | Diffusion denoising loss, contrastive loss | [√](https://github.com/MrGiovanni/TextoMorph) |
| Diff-CXR (2024) [181] | Diffusion model，Transformer | Report-to-CXR synthesis | Diffusion denoising loss，InfoNCE loss, BCE loss | [√](https://github.com/cstreiffer/cxr_diffusion) |
| Chest-diffusion (2024) [50] | Diffusion model, VAE | Report-to-CXR synthesis | Diffusion denoising loss， contrast loss | – |
| **Image-to-Image Synthesis** | |  |  |  |
| Ben-Cohen (2019) [298] | GAN | CT-to-PET translation | Adversarial loss, MSE loss, L1 loss | – |
| Jiao et al. (2020) [300] | GAN | US-to-MRI translation | Latent space loss, appearance loss, structural consistency loss, adversarial loss | – |
| sc-cycleGAN (2020) [53] | GAN | MR-to-CT translation | Adversarial loss, cycle-consistency loss, structure-consistency loss | – |
| Gong et al. (2020) [54] | GAN | MRI-to-PET translation | Adversarial loss, cycle-consistency loss | – |
| GLFC (2025) [304] | Mamba | CBCT-to-CT translation | Multiple contrast Loss | [√](https://github.com/HiLab-git/GLFC) |
| EGDiff (2024) [224] | Diffusion model | CBCT-to-CT translation | Diffusion denoising loss, MSE loss | – |
| DiffMa (2024) [301] | Diffusion model, Mamba | CT-to-MRI translation | Diffusion denoising loss, infoNCE loss | [√](https://github.com/wongzbb/DiffMaDiffusion-Mamba) |
| MIDiffusion (2024) [56] | Diffusion model | MRI cross-modality translation | Mutual information diffusion denoising loss | [√](https://github.com/mgh-ccni/midiffusion) |
| Yan et al. (2022) [307] | GAN | Multimodal MRI completion | Adversarial loss, cycle-consistency loss | – |
| Raad et al. (2024) [316] | GAN | Multimodal MRI completion | Adversarial loss, MAE loss | – |
| CKG–GAN (2024) [310] | GAN | Multimodal MRI completion | Cross-dimensional knowledge loss + adversarial | [√](https://github.com/QianWeiZhou/CKG-GAN) |
| Zhang et al. (2024) [309] | GAN | Multimodal MRI completion | Synthesis loss, reconstruction loss, adversarial loss, | – |
| AutoSyncoder (2020) [225] | GAN，VAE | Multimodal MRI completion | Adversarial loss, negative log-likelihood loss | – |
| I2I-Mamba (2024) [303] | Mamba | Multimodal MRI completion | Adversarial loss, pixel-wise loss | [√](https://github.com/icon-lab/I2I-Mamba) |
| ResViT (2022) [55] | Transformer | Multimodal MRI completion | L1 loss, adversarial loss | [√](https://github.com/icon-lab/ResViT) |
| MMT (2023) [305] | Transformer | Multimodal MRI completion | Synthesis loss, reconstruction loss, adversarial loss, | – |
| FgC2F-UDiff (2024) [308] | Diffusion model | Multimodal MRI completion | Diffusion denoising loss | [√](https://github.com/xiaojiao929/FgC2F-UDiff) |
| **Anatomically-Guided Image Synthesis** | | |  |  |
| CG-SAMR (2021) [311] | GAN | Anatomy-guided CT synthesis | Adversarial loss, confidence map loss,feature matching loss, shape consistency loss | [√](https://github.com/guopengf/CG-SAMR) |
| Shen et al. (2023) [312] | GAN | Anatomy-guided CXR synthesis | Reconstruction loss, Perceptual loss, Adversarial loss | – |
| Hou et al. (2023) [313] | GAN | Anatomy-guided fundus image synthesis | Wasserstein GAN loss, feature matching loss, KL-loss | – |
| Real-ESRGAN (2024) [57] | GAN | Anatomy-guided fundus image synthesis | Adversarial loss, perceptual loss, L1 loss, L1_seg loss | – |
| LN-Gen (2024) [314] | Diffusion model | Anatomy-guided rectal lymph nodes synthesis | Diffusion denoising loss, adapter loss | [√](https://github.com/schmidtkk/LN-Gen) |
| SegGuidedDiff (2024) [58] | Diffusion model | Anatomy-guided MRI synthesis | Diffusion denoising loss | [√](https://github.com/surjo0/SegGuidedDiff) |

- 1. **Treatment Phase: Enabling Precision Interventions**

In the treatment phase of clinical care, the integration of generative AI into radiotherapy and intraoperative navigation offers transformative potential for precision medicine. By modeling complex anatomical variations, capturing physiological motion, and supporting real-time clinical decision-making, generative models are increasingly bridging the gap between static preoperative imaging and dynamic, adaptive interventions. This section explores two key areas: dose prediction and planning in radiotherapy, and dynamic image synthesis for intraoperative navigation, as illustrated in Table S7.

- - 1. ***Generation for treatment planning***

In the clinical phase of radiotherapy, achieving precise intervention not only enhances treatment efficacy but also minimizes damage to normal tissues. However, this process is challenged by several factors. Significant anatomical variations among patients, along with the motion of tumors and adjacent organs, introduce uncertainty in localization. Moreover, limitations in image quality and acquisition speed have hindered the real‐time updating and optimization of treatment plans[317,318]. Generative AI models are increasingly employed to synthesize individualized dose maps and simulate anatomy in support of adaptive and personalized treatment.

Early models such as Cascade 3D U-Net introduced multi-scale CT feature fusion to model dose gradients in complex regions like the head and neck[319], while DoseNet applied a fully convolutional network to generate 3D dose distributions with high computational efficiency[59]. However, these models remained limited in their ability to capture long-range anatomical dependencies, such as the spatial relationships between pelvic tumors and neighboring organs. To address this, LSTM-based architecture[320] introduced sequence modeling for spatial continuity. and TransDose[22] enhanced rectal cancer dose prediction by combining transformer-based global extraction with superpixel GCN, reducing the HI (homogeneity index) by 0.216 and halving the errors in D* (dose at reference point) and V40 (volume receiving 40 Gy) compared with U-Net. Further refinement came with SP-DiffDose[321], which fused Swin Transformer features with a projection network to improve local dose gradients in anatomically ambiguous regions such as pancreatic cancer, achieving a dose score of 1.901 Gy, DVH Score of 1.533 Gy, and HI of 0.278. DiffDP[60] used conditional diffusion on CT and segmentation inputs to generate multiple plausible dose distributions, achieving lower dose errors (ΔHI = 0.0413, ΔD98 = 0.0402) than DeepLabV3+, supporting flexible planning in high-risk cases (e.g., lung tumors near the heart). Leveraging the Mamba architecture, MD-dose[61] achieved faster sampling and more accurate predictions, reducing the dose score by 2.650 Gy, the DVH score by 2.046 Gy, and the HI by 0.309 compared with C3D, thereby supporting real-time adaptive radiotherapy. Concurrently, generative models have been extended to imaging tasks: self-improving foundation models[18], volumetric image generation[322], and CBCT synthesis for real-time tracking[21] have been used to augment input data and support decision-making. Applications including patient-specific simulation[317], heterogeneous tumor modeling[318], and conditional brain tumor generation[323] further highlighted the promise of multimodal fusion and deep generative learning in clinical radiotherapy workflows.

In summary, these dose prediction techniques offer significant advantages in terms of improving predictive accuracy, reducing trial-and-error costs, and optimizing treatment plans, thereby providing robust data support for personalized radiotherapy. Moreover, this body of research lays the foundation for subsequent advancements in real-time dynamic image synthesis and intraoperative navigation, which will be addressed in the following section.

- - 1. ***Intraoperative navigation: dynamic image synthesis***

Intraoperative navigation benefits from dynamic image synthesis, which aims to generate real-time, patient-specific visualizations that reflect both anatomical structure and physiological motion. These techniques support tasks such as cardiovascular function monitoring and radiotherapy adaptation by modeling temporal dynamics and suppressing motion artifacts.

In 2D+t synthesis, early research focused on generating dynamic sequences from sparse or single-frame data. For instance, a super-resolution GAN[62] accelerated cardiac MRI generation while preserving phase-specific morphology. DragNet[6] used deformable registration to reconstruct full cardiac cycles from static input, mitigating motion blur from sparse sampling and achieving a Dice score of 82.4%, RMSE of 0.044, and the lowest temporal gradient (0.21 ± 0.04). Video diffusion models[324] improved structural consistency in dynamic echocardiography, achieving an FID of 17.65 for frames, compared to 75.36 from Echo from Noise. HeartBeat[64] integrated ECG signals with hemodynamic features to produce personalized cardiac motion, achieving an FID of 25.23, FVD of 6.08, and SSIM of 0.66 on the A2C view, demonstrating the value of multimodal conditioning. Building on these efforts, a cascaded video diffusion model[63] introduced hierarchical refinement of motion and texture using semantic and anatomical cues, showing high fidelity (SSIM = 0.919, PSNR = 27.9) and strong performance on future tumor prediction (DSC = 0.719) and source segmentation (DSC = 0.849). Endora[325], a diffusion-based framework designed for dynamic medical procedures like endoscopy, demonstrated strong video generation performance (FID = 13.41, FVD = 460.7 on Colonoscopic data) and improved diagnostic classification (F1 = 87.0 on PolyDiag). By leveraging procedural priors and domain-specific context, it offered insights that may be applicable to advancing 2D+t generation techniques. Collectively, these models illustrate a shift toward semantically guided, feature-aware generation, enhancing the realism and controllability of dynamic synthesis. However, current 2D+t methods still face notable limitations in capturing complex out-of-plane motion, maintaining long-range temporal coherence, and modeling full volumetric dynamics from limited spatial views.

In 3D+t (4D) synthesis, methods focus on constructing evolving volumetric sequences from incomplete or low-resolution inputs. The temporally aware 3D GAN framework proposed in[65] combined respiratory motion compensation with dynamic cardiac MRI reconstruction, effectively capturing continuous cardiac motion and substantially reducing respiratory-induced artifacts. Regarding 4D cardiac MRI generation, most existing approaches[43,268–270] predicted deformation fields for the initial and final frames and interpolated these fields to generate intermediate frames; however, such methods were limited in handling large-scale motion. Newer approaches, such as 4D CT synthesis from sparse CBCT[66] and cross-modal CBCT-to-MRI translation[67] have respectively generated 4D synthetic CT from sparse-view CBCT and achieved cross-modal dynamic synthesis from 4D CBCT to 4D MRI, effectively addressing challenges in complex nonlinear motion mapping. Furthermore, the deep prior image-constrained motion compensation framework (DPI-MoCo)[326] incorporated a motion trajectory prediction module into 4D CBCT reconstruction, which reduced motion distortions and yielded quantitative improvements (RMSE = 0.76 × 10⁻³, PSNR = 28.45, SSIM = 0.9054). Similarly, another work[327] employed a feature disentanglement mechanism to extract differential features across multiple 3D/4D MRI sequences, enhancing quantitative analysis such as myocardial perfusion and tumor segmentation, with whole-tumor results reaching a Dice score of 0.923 and ASSD of 1.242, and no statistically significant difference observed when using only T1Gd and FLAIR sequences (p > 0.05).

Although these various strategies—including registration, GANs, and diffusion models have shown promising initial results in modeling spatiotemporal continuity and suppressing motion artifacts, they still face significant challenges. In particular, limitations in real-time performance, generalization to complex motion patterns, and cross-modal data consistency continue to restrict their clinical applicability and robustness. Notably, the text-driven 4D cardiac cine MRI synthesis method[68] introduces disease description texts as prior information to enable controlled synthesis of pathology-specific motion patterns. This innovative approach not only enhances the semantic accuracy and pathological specificity of the synthesized images, but also offers a promising pathway to overcome current limitations, potentially advancing intelligent diagnostics and precision therapy.

**Table S7**. Summary of publications on treatment phase: enabling precision interventions.

| **Publication (Year)** | **Model** | **Application** | **Loss Function** | **Link** |
| --- | --- | --- | --- | --- |
| **Generation for Treatment Planning** | | | | |
| DoseNet (2018) [59] | CNN | Radiation dose prediction | L2 loss | [√](https://github.com/mkdermo/DoseNet) |
| C3D (2021) [319] | CNN | Radiation dose prediction | L1 loss | [√](https://github.com/LSL000UD/RTDosePrediction) |
| Radonic et al. (2024) [320] | CNN | Radiation dose prediction | MSE loss | – |
| TransDose (2023) [22] | Transformer | Radiation dose prediction | Cross entropy loss，Charbonnier Loss | – |
| VQGAN_TATrans (2024) [323] | GAN，VAE，Transformer | Brain tumor prediction | Pixel differences loss，perceptual loss，feature matching loss，gradient loss，codebook loss | [√](https://github.com/IMICSLab/Brain_VQGAN_TATrans) |
| PC-DDPM (2024) [21] | Diffusion model | Real-time tumor tracking | Diffusion denoising loss，cycle-consistency loss | – |
| DiffDP (2023) [60] | Diffusion model | Radiation dose prediction | Diffusion denoising loss | [√](https://github.com/scufzh/DiffDP) |
| SP-DiffDose (2023) [321] | Diffusion model，Transformer | Radiation dose prediction | Diffusion denoising loss | – |
| MD-Dose (2024) [61] | Diffusion model，Mamba | Radiation dose prediction | Diffusion denoising loss | [√](https://github.com/LinjieFu-U/mamba_dose) |
| **Intraoperative navigation: Dynamic image synthesis** | | | | |
| SVIN (2020) [268] | CNN | 4D dynamic MRI synthesis | Similarity loss, smoothness regularization loss, regression loss | [√](https://github.com/guoyu-niubility/SVIN) |
| DragNet (2023) [6] | CNN | 2Dt cardiac MR synthesis | ELBO loss, KL loss, similarity loss | – |
| Quintero et al. (2024) [67] | CNN | 4D dynamic MRI synthesis | RMSE loss | – |
| MPVF (2023) [269] | CNN, Transformer | 4D dynamic MRI synthesis | Charbonnier loss | [√](https://github.com/Tzu-Ti/MPVF) |
| UVI-Net (2024) [270] | CNN, Transformer | 4D dynamic MRI synthesis | NCC loss, gradient loss | [√](https://github.com/jungeun122333/UVI-Net) |
| TAV-GAN (2021) [65] | GAN | 4D dynamic MRI synthesis | Temporally aware loss, SSIM loss, L1 loss | – |
| Thummerer et al. (2022) [66] | GAN | 4D CT synthesis | MSE loss | – |
| REGAIN (2023) [62] | GAN | 2Dt cardiac MRI enhancement | L1 fast-Fourier transform loss | – |
| Seq2Seq (2024) [327] | GAN | 3D/4D MRI synthesis | L1 loss, perceptual loss, adversarial loss, cycle-consistent loss | [√](https://github.com/fiy2W/mri_seq2seq) |
| DPI-MoCo (2024) [326] | GAN | 4D CBCT reconstruction | MSE loss, GAN loss, NCC loss, smooth loss | – |
| DDM (2022) [43] | Diffusion model | 4D dynamic MRI synthesis | Diffusion denoising loss, NCC loss, KL loss | [√](https://github.com/torchDDM/DDM) |
| Reynaud et al. (2023) [63] | Diffusion model, Transformer | Echocardiography video synthesis | Diffusion denoising loss | [√](https://github.com/HReynaud/EchoDiffusion) |
| HeartBeat (2024) [64] | Diffusion model, VAE | Echocardiography video synthesis | Diffusion denoising loss | – |
| Endora (2024) [325] | Diffusion model, Transformer | Endoscopy video synthesis | Diffusion denoising loss | [√](https://endora-medvidgen.github.io/) |

- 1. **Prognosis Phase: Longitudinal & Personalized Medicine**

Generative medical imaging techniques have demonstrated significant clinical potential in longitudinal prognostic analysis and personalized medicine, as summarized in Table S8. By leveraging deep modeling of patients’ multi-temporal imaging data, these approaches can simulate dynamic disease progression, predict tissue degenerative changes, and quantify prognostic risk, thereby providing data-driven support for clinical decision-making.

- - 1. ***Tumor growth simulation and treatment response prediction***

In recent years, the integration of multimodal imaging data (e.g., MRI, CT) with patient-specific biomarkers (such as EGFR mutation status) has established a novel data foundation for predicting tumor growth and treatment response. For instance, a study[23] introduced a treatment-aware diffusion probabilistic model that simulated the 3D growth patterns and invasive behavior of gliomas using longitudinal MRI and molecular pathology data, boosting future tumor prediction accuracy (DSC from 0.556 to 0.719; +16.3%). Similarly, SADM[69] adopted a novel design that enabled learning of longitudinal dependencies even in the presence of missing data during training, and supported autoregressive generation of image sequences during inference. Another model[328] presented a universal tumor synthesis framework that fused cross-modal data to generate high-quality synthetic tumors, improving sensitivity to texture and morphological variations. In related work[70], radiomics features extracted from synthetic MRI enhanced glioblastoma survival prediction across multi-center datasets, supporting personalized radiotherapy planning. A patient-specific deep learning framework[329] presented a patient-specific deep learning framework for real-time, label-free tumor tracking, enabling non-invasive monitoring of treatment response. Complementing this, the cross-tumor CT foundation model[71] established a unified interpretive platform across cancer types, achieving state-of-the-art performance on 45 of 46 oncology tasks and improving radiologists’ sensitivity by up to 31.4%, precision by 24.9%, and segmentation efficiency by 78.2%. Together, these approaches demonstrate how generative models can effectively simulate tumor dynamics and predict individualized treatment outcomes, advancing the development of scalable, multimodal tools for personalized oncology.

- - 1. ***Spatiotemporal modeling of neurodegenerative disease progression***

For neurodegenerative diseases such as Alzheimer’s disease (AD), tracking structural brain changes over time is critical for early diagnosis, disease staging, and therapeutic planning. Longitudinal MRI synthesis techniques have emerged as valuable tools for capturing subtle, progressive degeneration across brain regions, enabling precise spatiotemporal modeling of disease trajectories[72,330]. To address limitations in data availability and temporal resolution, a work[73] proposed a hybrid deep learning framework that integrated DCGAN and SRGAN to generate synthetic MRI sequences corresponding to different AD stages. This method achieved classification and prediction accuracies as high as 99.7%, demonstrating its potential in compensating for real-world data scarcity and improving progression staging. Building on this, TADM[74] incorporated a pre-trained Brain Age Estimator (BAE) to guide learning of intensity-based structural changes over time. By learning the distribution of inter-scan variations, TADM predicted future MRI volumes based on baseline scans. Compared to conventional approaches, it reduced mean brain volume error by 24% and improved similarity metrics by 4%, offering both anatomical accuracy and clinical reliability. These approaches not only provided a visual and quantitative representation of neurodegenerative progression, but also supported prognostic modeling for treatment response and optimal drug timing. As such, spatiotemporal generative modeling holds significant promise for advancing personalized, stage-aware intervention strategies in the management of AD and other neurodegenerative disorders.

- - 1. ***Translating multimodal generative prognostics into clinical practice***

A key challenge in prognostic modeling lies in the integration of diverse imaging modalities and clinical variables for robust risk stratification and treatment guidance. Early efforts leveraged generative models such as GANs to explore synthetic data-driven prognostic modeling. For example, a work[75] used a conditional GAN to generate synthetic cardiac aging images, aiding early diagnosis of diastolic dysfunction. Likewise, another work[331] employed a GAN-convolutional framework to predict long-term MRI changes, offering insights for aging-related prognostic assessment. In neurodegeneration, a new work[76] introduced a latent diffusion model that improved brain volume prediction in Alzheimer’s patients by 22% and enhanced image similarity by 43%. In oncology, a self-evolving foundation model[18] demonstrated that augmenting training with synthetic data improved HER2 mutation detection accuracy from 79.2% to 94.0% and EGFR classification accuracy from 81.5% to 95.4%, thereby supporting more precise patient stratification and treatment planning. For cerebrovascular prognosis, an end-to-end deep model[77] used synthetic CT to predict hematoma expansion, achieving specificity of 0.91. In parallel, radiomics features derived from synthetic MRI[70] enhanced glioblastoma survival prediction across multi-center datasets, reinforcing the value of high-fidelity synthetic data in clinical radiotherapy planning. Collectively, these advances signal a paradigm shift toward generative, multimodal prognostics that move beyond static risk scores to dynamic, individualized disease forecasting. Bridging the gap to clinical practice will require not only technical improvements, such as domain adaptation and model interpretability, but also careful alignment with clinical workflows and decision-making requirements.

By capturing dynamic, multimodal disease trajectories, generative imaging models offer powerful tools for prognosis across tumor, neurological, and cardiovascular domains. Nonetheless, clinical translation at scale requires further work in domain adaptation, temporal modeling, and model interpretability. Future progress in these areas is expected to enhance robustness and generalizability across diverse clinical environments, reinforcing the role of generative models in precision medicine and personalized care.

**Table S8.** Summary of publications on prognosis phase: longitudinal & personalized medicine.

| **Publication (Year)** | **Model** | **Application** | **Loss Function** | **Link** | |
| --- | --- | --- | --- | --- | --- |
| Moya-Sáez et al. (2022) [70] | CNN | Glioblastoma survival prediction | L1 loss | | – |
| EfficientNet B0 (2024) [77] | CNN | Hematoma expansion prediction | Focal loss | | [√](https://github.com/NIC-VICOROB/HE-prediction-SynthCT) |
| DaniNet (2019) [330] | GAN | Mimic disease progression | Biological constraints loss, Deformation loss | | – |
| GP-GAN (2020) [72] | GAN | Brain tumor growth prediction | Adversarial loss, L1 loss, Dice loss | | – |
| Song et al. (2023) [331] | GAN | Longitudinal MRI prediction | Adversarial loss, Binary cross-entropy loss, Gradient difference loss | | – |
| DCGAN and SRGAN (2024) [73] | GAN | Alzheimer's disease progression | Adversarial loss, MSE loss, VGG Loss | | – |
| TADM (2024) [74] | Diffusion model | Brain neurodegenerative prediction | Diffusion denoising loss | | [√](https://github.com/MattiaLitrico/TADM-Temporally-Aware-Diffusion-Model-for-Neurodegenerative-Progression-on-Brain-MRI) |
| BrLP (2024) [76] | Diffusion model | Disease progression prediction | Diffusion denoising loss | | [√](https://github.com/LemuelPuglisi/BrLP) |
| DiffTumor (2024) [328] | Diffusion model，VAE | Generalizable tumor synthesis | Diffusion denoising loss | | [√](https://github.com/MrGiovanni/DiffTumor) |
| PASTA (2025) [71] | Diffusion model，VAE | Tumor synthesis Foundation model | Diffusion denoising loss | | [√](https://github.com/LWHYC/PASTA) |
| SADM (2023) [69] | Diffusion model, AR | Longitudinal MRI Generation | Diffusion denoising loss | | [√](https://github.com/ubc-tea/SADMLongitudinal-Medical-Image-Generation) |
| TaDiff (2025) [23] | Diffusion model | Longitudinal MRI Generation and Glioma Growth Prediction | Diffusion denoising loss | | [√](https://github.com/samleoqh/TaDiff-Net) |

1. **More Details on Overview of Public Datasets**

The advancement of generative AI in medical imaging is closely linked to the availability of large-scale, high-quality, and multi-modal public datasets. These datasets not only provide essential resources for model training but also serve as standardized benchmarks for evaluating generalization, fidelity, and clinical applicability across diagnostic, therapeutic, and prognostic tasks. Comprehensive repositories such as the UK Biobank[78] offer diverse imaging modalities, including MRI, CT, ultrasound, and fundus photography, collected from over 500,000 participants. The Cancer Imaging Archive (TCIA)[79] provides expertly annotated CT, MRI, and PET scans across a wide range of tumor types. Although originally developed for tasks like classification, segmentation, and reconstruction, these datasets now underpin a wide range of generative applications, including image synthesis, modality translation, and anomaly simulation. In addition, open platforms such as Grand Challenge and Kaggle facilitate standardized access to datasets spanning X-ray, histopathology, cardiac MRI, and endoscopy. These platforms support reproducible benchmarking for generative models across tasks such as 2D/3D image generation, report synthesis, and multi-modal alignment. Overall, public datasets constitute the foundation of generative modeling in medical imaging. This section provides an overview of widely used datasets in the field, as summarized in Table S9.

***Organ- and region-specific CT and MRI dataset Datasets.*** Multi-organ and whole-body imaging datasets form the backbone of generative modeling across diverse anatomical regions. DeepLesion[80] and ULS[332] provide extensive lesion annotations and 3D whole-body CT volumes, supporting generative tasks such as lesion synthesis, multi-organ reconstruction, and cross-modality translation. Notably, the recently released PreCT-160K[81] dataset represents the largest known CT imaging corpus to date, with 160,000 CT scans spanning a wide range of anatomical sites and clinical conditions, offering unprecedented scale for training high-capacity generative models. The TotalSegmentator series[82,333] further provides fine-grained segmentation across over 10 anatomical structures, enabling anatomically faithful synthesis and training of anatomically guided generative models. Additionally, AutoPET and AutoPETIII[85] , based on paired PET-CT imaging, further support hybrid synthesis and modality fusion for tumor representation learning.

For head and neck imaging, datasets such as INSTANCE2022[334], SegRap2023[335], and HECKTOR2022[86] offer PET-CT and CT volumes with expert tumor and organ-at-risk (OAR) annotations, making them ideal for training cross-modality synthesis and radiotherapy planning models. In neuroimaging, collections like BraTS21[83], BraTS-MEN[336], fastMRI_Brain[337], IXI[338], and AOMIC[339] provide volumetric brain MRIs for brain tumor synthesis, progression modeling, and longitudinal prediction. In neuroimaging, Diff5T[87] provides high-field diffusion MRI with raw k-space data, advancing reconstruction and microstructural modeling. Cardiac imaging is supported by datasets such as ACDC[84], M&Ms[340],[341], OCMR[342], and EchoNet-Dynamic[63], which contain both 2Dt and 3D MRI as well as ultrasound videos. These are crucial for developing dynamic generative models (e.g., 4D cine MRI synthesis, cardiac motion prediction) and dose adaptation frameworks in cardiovascular interventions. Abdominal datasets including FLARE[343,344] , AbdomenCT-1K[345], and PI-CAI[346] offer high-resolution CT/MRI volumes annotated for multi-organ segmentation, supporting synthetic image augmentation and simulation of disease-specific anatomical changes. These datasets provide foundational support for generative tasks like low-dose reconstruction, anatomical synthesis, and cross-organ correlation modeling.

***Pathology and High-resolution Microscopy.*** In the domain of computational pathology, datasets like CPIA[347], PatchCamelyon[88], BreakHis[348], NAFLD[349], and MIST-HER2[350] encompass millions of whole-slide images (WSIs), supporting self-supervised pretraining and high-resolution image generation. These datasets are crucial for exploring style-transfer synthesis (e.g., H&E to IHC), anomaly simulation, and histological progression modeling in cancer[351].

***Ultrasound, OCT, and Fundus Datasets.*** Ultrasound imaging, known for its portability and real-time capabilities yet susceptibility to noise, benefits from datasets such as TG3K[352], EchoNet-LVH[353], and EndoSLAM[354], which support generative tasks including denoising, quality enhancement, and anatomical segmentation. In ophthalmic imaging, OCT datasets like OCT2017[90] and Retinal OCT-C8[355] enable super-resolution, layer segmentation, and disease-specific synthesis. Additionally, fundus image datasets such as ODIR-5K[356], LAG[357], and AIROGS[358] provide large-scale, annotated images for generative modeling tasks like lesion synthesis, cross-disease domain translation, and image-to-report generation, further expanding the applicability of generative models in ocular disease analysis.

***Multimodal Image‐Text Datasets.*** The integration of visual and textual modalities is becoming increasingly essential in generative medical imaging, particularly for tasks such as radiology report generation, text-conditioned synthesis, and multimodal retrieval. A variety of large-scale datasets have emerged to support these applications. In chest imaging, resources like CheXpertPlus[91], Medical-CXR-VQA[359], and PadChest[360] facilitate vision-language pretraining and VQA-style tasks. In pathology, datasets such as Quilt-1M[89] and OpenPath[361] provide over one million image–text pairs derived from clinical annotations and social media sources. More general-purpose datasets like Medtrinity-25M[93], MedICaT [92] and PMC-OA[362] offer paired images and captions from biomedical literature, widely used in training foundation models for captioning and contrastive learning. Additionally, multimodal datasets such as Duke Breast Cancer MRI[363] and I-SPY2[364] enable radiogenomic modeling by linking imaging features with genomic and clinical data.

As generative models in medical imaging continue to evolve, the importance of high-quality, well-curated training data has become increasingly evident. Public datasets play a central role in this progress by enabling model development, performance evaluation, and clinical validation across diverse modalities, anatomical regions, and disease types. While these datasets have contributed to improvements in robustness and generalizability, several challenges persist, such as domain discrepancies, annotation inconsistencies, and the limited availability of dynamic or longitudinal imaging data. Addressing these issues through improved standardization, collaborative curation, and the responsible integration of synthetic data will be key to supporting the reliable and clinically meaningful deployment of generative models in real-world settings.

**Table S9**. The publicly available datasets.

| **Dataset** | | **Modalities** | | **Scale** | **Link** | | | |
| --- | --- | --- | --- | --- | --- | --- | --- | --- |
| **Whole body** |  | |  | | |  | |  |
| MedMNIST [365] | | 2D & 3D | | 708K 2D images & 10K 3D volumes | | | [√](https://doi.org/10.5281/zenodo.10519652) | |
| DeepLesion[80] | | 2D CT | | 32.7K images | | | [√](https://nihcc.app.box.com/v/DeepLesion) | |
| ULS[332] | | 3D CT | | 38.8K volumes | | | [√](https://uls23.grand-challenge.org/) | |
| PreCT-160K[81] | | 3D CT | | 160K volumes | | | [√](https://huggingface.co/datasets/Luffy503/PreCT-160K) | |
| FLARE24 Task1[366] | | 3D CT | | 10K volumes | | | [√](https://www.codabench.org/competitions/2319/) | |
| CT-ORG[367] | | 3D CT | | 140 volumes | | | [√](https://wiki.cancerimagingarchive.net/pages/viewpage.action?pageId=61080890) | |
| TotalSegmentator[82] | | 3D CT | | 1204 volumes | | | [√](https://zenodo.org/records/6802614) | |
| TotalSegmentator v2[82] | | 3D CT | | 1228 volumes | | | [√](https://zenodo.org/records/10047292) | |
| AutoPET[85] | | 3D PET-CT | | 1014 volumes | | | [√](https://autopet.grand-challenge.org/Dataset/) | |
| AutoPETIII[85] | | 3D PET-CT | | 1614 volumes | | | [√](https://autopet-iii.grand-challenge.org/autopet-iii/) | |
| TotalSegmentator MRI[333] | | 3D MRI | | 298 volumes | | | [√](https://zenodo.org/records/11367005) | |
| TotalSegmentator MRI v2[333] | | 3D MRI | | 616 volumes | | | [√](https://zenodo.org/records/14710732) | |
| CPIA[347] | | Pathology | | 21.4M WSI | | | [√](https://github.com/zhanglab2021/CPIA_Dataset) | |
| NAFLD[349] | | Pathology | | 119.8K WSI | | | [√](https://osf.io/gqutd/) | |
| **Head and Neck** | |  | |  | | |  | |
| INSTANCE2022[334] | | 3D CT | | 200 volumes | | | [√](https://instance.grand-challenge.org/) | |
| SegRap2023[335] | | 3D CT | | 200 volumes | | | [√](https://segrap2023.grand-challenge.org/dataset/) | |
| HECKTOR2022[86] | | 3D PET-CT | | 882 volumes | | | [√](https://hecktor.grand-challenge.org/Data/) | |
| fastMRI_Brain[337] | | 2D MRI | | 6970 images | | | [√](https://fastmri.med.nyu.edu/) | |
| IXI Dataset[338] | | 3D MRI | | 600 volumes | | | [√](https://brain-development.org/ixi-dataset/) | |
| AOMIC[339] | | 3D MRI | | 1370 volumes | | | [√](https://nilab-uva.github.io/AOMIC.github.io/) | |
| BraTS21[83] | | 3D MRI | | 2040 volumes | | | [√](https://www.synapse.org/Synapse:syn25829067/wiki/610863) | |
| BraTS2023-MEN[336] | | 3D MRI | | 1650 volumes | | | [√](https://www.synapse.org/Synapse:syn51156910/wiki/627000) | |
| CrossMoDA2021[368] | | 3D MRI | | 349 volumes | | | [√](https://crossmoda.grand-challenge.org/) | |
| CrossMoDA2023[368] | | 3D MRI | | 983 volumes | | | [√](https://www.synapse.org/Synapse:syn51236108/files/) | |
| Diff5T[87] | | 3D MRI | | 14.65K volumes | | | [√](https://doi.org/10.57760/sciencedb.25122) | |
| TN3K[352] | | 2D US | | 3493 images | | | [√](https://github.com/haifangong/TRFE-Net-for-thyroid-nodule-segmentation) | |
| TN-SCUI2020[369] | | 2D US | | 4554 images | | | [√](https://tn-scui2020.grand-challenge.org/Home/) | |
| Ultrasound Nerve Segmentation[370] | | 2D US | | 11.1K images | | | [√](https://www.kaggle.com/c/ultrasound-nerve-segmentation/data) | |
| OCT2017[90] | | OCT | | 35.1K images | | | [√](https://data.mendeley.com/datasets/rscbjbr9sj/2) | |
| Retinal OCT-C8[355] | | OCT | | 24K images | | | [√](https://www.kaggle.com/datasets/obulisainaren/retinal-oct-c8) | |
| ODIR-5K[356] | | Fundus | | 5000 images | | | [√](https://odir2019.grand-challenge.org/dataset/) | |
| LAG[357] | | Fundus | | 11.7K images | | | [√](https://github.com/smilell/AG-CNN?tab=readme-ov-file) | |
| Diabetic Retinopathy Arranged[371] | | Fundus | | 35.1K images | | | [√](https://tianchi.aliyun.com/dataset/93926) | |
| AIROGS[358] | | Fundus | | 101.4K images | | | [√](https://airogs.grand-challenge.org/data-and-challenge/) | |
| PatchCamelyon[88] | | Pathology | | 327.7K WSI | | | [√](https://github.com/basveeling/pcam) | |
| OSCC[372] | | Pathology | | 1224 WSI | | | [√](https://data.mendeley.com/datasets/ftmp4cvtmb/1) | |
| **Chest** | |  | |  | | |  | |
| CheXchoNet[373] | | 2D X-ray | | 71.6K images | | | [√](https://physionet.org/content/chexchonet/1.0.0/) | |
| BRAX[374] | | 2D X-ray | | 40.9K images | | | [√](https://physionet.org/content/brax/1.1.0/) | |
| SIIM-FISABIO-RSNA COVID-19[375] | | 2D X-ray | | 7597 images | | | [√](https://www.kaggle.com/competitions/siim-covid19-detection/data) | |
| LIDC-IDRI[376] | | 2D CT | | 1010 images | | | [√](https://www.cancerimagingarchive.net/collection/lidc-idri/) | |
| SARS-COV-2 Ct-Scan[377] | | 2D CT | | 2482 images | | | [√](https://www.kaggle.com/datasets/plameneduardo/sarscov2-ctscan-dataset) | |
| ATM22[378] | | 3D CT | | 500 volumes | | | [√](https://atm22.grand-challenge.org/) | |
| LUNA16[379] | | 3D CT | | 888 volumes | | | [√](https://luna16.grand-challenge.org/Home/) | |
| LNQ2023[380] | | 3D CT | | 513 volumes | | | [√](https://lnq2023.grand-challenge.org/lnq2023/) | |
| fastMRI_Breast[337] | | 2D MRI | | 300 images | | | [√](https://fastmri.med.nyu.edu/) | |
| ISPY1-Tumor-SEG-Radiomics[381] | | 3D MRI | | 483 volumes | | | [√](https://www.cancerimagingarchive.net/analysis-result/ispy1-tumor-seg-radiomics/) | |
| ACRIN-Contralateral-Breast-MR[382] | | 3D MRI | | 984 volumes | | | [√](https://www.cancerimagingarchive.net/collection/acrin-contralateral-breast-mr/) | |
| BUSI[383] | | US | | 780 images | | | [√](https://scholar.cu.edu.eg/?q=afahmy/pages/dataset) | |
| TDSC-ABUS2023[384] | | US | | 200 volumes | | | [√](https://tdsc-abus2023.grand-challenge.org/TDSC-ABUS2023/) | |
| Breakhis[348] | | Pathology | | 7909 WSI | | | [√](https://opendatalab.com/OpenDataLab/BreakHis/explore/main) | |
| WSSS4LUAD[385] | | Pathology | | 10K WSI | | | [√](https://wsss4luad.grand-challenge.org/WSSS4LUAD/) | |
| MIST-HER2[350] | | Pathology | | 22.7K WSI | | | [√](https://drive.google.com/drive/folders/146V99Zv1LzoHFYlXvSDhKmflIL-joo6p?usp=sharing) | |
| **Cardiac** | |  | |  | | |  | |
| ACDC[84] | | 3D MRI | | 150 volumes | | | [√](https://humanheart-project.creatis.insa-lyon.fr/database/#collection/637218c173e9f0047faa00fb) | |
| MICCAI 2024 CARE LAScarQS++[386] | | 3D MRI | | 194 volumes | | | [√](https://zmic.org.cn/care_2024/track2/#data) | |
| M&Ms Challenge[340] | | 3D MRI | | 375 volumes | | | [√](https://mega.nz/folder/FxAmhbRJ#Dwugf8isRSR9CCZ6Qnza4w ) | |
| M&Ms-2 Challenge[341] | | 3D MRI | | 360 volumes | | | [√](https://mega.nz/file/Y1p0nSqa#Z68ab1FAq3rL1SRR-xHJZ_sgBMQOUZJAIHV2Qbsw_Ps) | |
| OCMR[342] | | 2Dt MRI | | 165 volumes | | | [√](https://www.ocmr.info/download/) | |
| Harvard Cardiac MR Center Dataverse[387] | | 2Dt MRI | | 108 volumes | | | [√](https://dataverse.harvard.edu/dataset.xhtml?persistentId=doi:10.7910/DVN/CI3WB6) | |
| CMRxRecon[388] | | 2Dt MRI | | 300 volumes | | | [√](https://www.synapse.org/Synapse:syn51471091/datasets/) | |
| Cardiac MRI Dataset[389] | | 2Dt MRI | | 7980 volumes | | | [√](https://data.nvision.eecs.yorku.ca/datasets/MRI/mrimages.tar.gz) | |
| Cardiac super-resolution label maps[390] | | 2Dt MRI | | 1331 volumes | | | [√](https://data.mendeley.com/datasets/pw87p286yx/1) | |
| GANcMRI[391] | | US | | 45.5K videos | | | [√](https://biobank.ndph.ox.ac.uk/showcase/field.cgi?id=31085) | |
| EchoNet-LVH[353] | | US | | 12K videos | | | [√](https://echonet.github.io/lvh/) | |
| EchoNet-Dynamic[392] | | US | | 10K videos | | | [√](https://echonet.github.io/dynamic/index.html) | |
| **Abdomen** | |  | |  | | |  | |
| FLARE2022[343] | | 3D CT | | 2300 volumes | | | [√](https://flare22.grand-challenge.org/Dataset/) | |
| FLARE2023[344] | | 3D CT | | 4500 volumes | | | [√](https://codalab.lisn.upsaclay.fr/competitions/12239#learn_the_details-dataset) | |
| AbdomenCT-1K[345] | | 3D CT | | 1112 volumes | | | [√](https://github.com/JunMa11/AbdomenCT-1K) | |
| AbdomenAtlas 1.0 Mini[393] | | 3D CT | | 5195 volumes | | | [√](https://huggingface.co/datasets/AbdomenAtlas/AbdomenAtlas1.0MiniBeta) | |
| UW-Madison GI Tract Image [394] | | 2D MRI | | 38.5K images | | | [√](https://www.kaggle.com/competitions/uw-madison-gi-tract-image-segmentation/overview) | |
| PI-CAI[346] | | 3D MRI | | 1500 volumes | | | [√](https://zenodo.org/records/6624726) | |
| FLARE 2024 Task3[395] | | 3D MRI | | 4817 volumes | | | [√](https://www.codabench.org/competitions/2296/) | |
| ISBI 2025 FUGC[396] | | US | | 890 images | | | [√](https://zenodo.org/records/14305302) | |
| LIMUC[397] | | US | | 1043 videos | | | [√](https://zenodo.org/records/5827695#.Yi8GJ3pByUk) | |
| SUN[398] | | US | | 1018 videos | | | [√](http://amed8k.sundatabase.org/) | |
| EndoSLAM[354] | | US | | 1020 videos | | | [√](https://github.com/CapsuleEndoscope/EndoSLAM) | |
| RenalCell[399] | | Pathology | | 625.1K WSI | | | [√](https://zenodo.org/records/6528599) | |
| GasHisSDB[400] | | Pathology | | 245.2K images | | | [√](https://gitee.com/neuhwm/GasHisSDB) | |
| SegPANDA200[401] | | Pathology | | 100.9K images | | | [√](https://drive.google.com/drive/folders/1zg_C37B_1HR6miRFuTwPKmueaJzvO-GD) | |
| NCT-CRC-HE[402] | | Pathology | | 100K WSI | | | [√](https://zenodo.org/records/1214456) | |
| **Others** | |  | |  | | |  | |
| SPIDER (Spine)[403] | | 3D MRI | | 257 volumes | | | [√](https://zenodo.org/records/10159290) | |
| Wrist Dataset[404] | | 3Dt MRI | | 55 volumes | | | [√](https://data.mendeley.com/datasets/9kx5xp7h6d/2) | |
| fastMRI_knee[337] | | 2D MRI | | 1398 images | | | [√](https://fastmri.med.nyu.edu/) | |
| SKM-TEA (Knee)[405] | | 3D MRI | | 155 volumes | | | [√](https://stanfordaimi.azurewebsites.net/datasets/4aaeafb9-c6e6-4e3c-9188-3aaaf0e0a9e7) | |
| MRNet (Knee)[122] | | 3D MRI | | 1370 volumes | | | [√](https://stanfordmlgroup.github.io/competitions/mrnet/) | |
| **Multimodal Dataset** | |  | |  | | |  | |
| Medical-CXR-VQA[359] | | X-ray-Text | | 377K images, 780K texts | | | [√](https://github.com/Holipori/Medical-CXR-VQA) | |
| PadChest[360] | | X-ray-Text | | 160K images, 109K texts | | | [√](http://bimcv.cipf.es/bimcv-projects/padchest/) | |
| CheXpertPlus[91] | | X-ray-Text | | 223K images, 223K texts | | | [√](https://stanfordaimi.azurewebsites.net/datasets/5158c524-d3ab-4e02-96e9-6ee9efc110a1) | |
| Quilt-1M[89] | | Pathology-Text | | 1M images, 1M texts | | | [√](https://quilt1m.github.io/) | |
| OpenPath[361] | | Pathology-Text | | 208K images, 208K texts | | | [√](https://huggingface.co/spaces/vinid/webplip) | |
| Medtrinity-25M[93] | | Multimodal Images-Text | | 25M images, 25M texts | | | [√](https://github.com/UCSC-VLAA/MedTrinity-25M) | |
| PMC-OA[362] | | Multimodal Images-Text | | 1.6M images, 1.6M texts | | | [√](https://huggingface.co/datasets/axiong/pmc_oa) | |
| MedICaT[92] | | Multimodal Images-Text | | 217K images, 217K texts | | | [√](https://github.com/allenai/medicat) | |
| Duke Breast Cancer MRI[363] | | Genomic&MRI-Clinical data | | 922 cases | | | [√](https://sites.duke.edu/mazurowski/resources/breast-cancer-mri-dataset/) | |
| I-SPY2[364] | | MRI-Clinical data | | 719 cases | | | [√](https://www.cancerimagingarchive.net/collection/ispy2/) | |

1. **More Details on Evaluation Methods for Generative Models in Medical Imaging**

Here we present more details on each of the evaluation methods that we introduced in the main paper.

- 1. **Low-Level Evaluation:** **Pixel Fidelity**

Low-level evaluation metrics focus on quantifying pixel-wise similarity between the generated image and the ground truth. They are most effective for tasks involving image reconstruction, denoising, or super-resolution, where spatial accuracy is essential.

***MSE / MAE / RMSE /*** ***PSNR***[406]***:*** Mean Squared Error (MSE) and Mean Absolute Error (MAE) are classical pixel-wise metrics that quantify the average intensity difference between generated and reference images. MSE applies a quadratic penalty, making it more sensitive to outliers, whereas MAE applies a uniform linear penalty, offering more robustness to extreme errors. Root Mean Squared Error (RMSE), the square root of MSE, restores the original intensity unit, improving interpretability. Peak Signal-to-Noise Ratio (PSNR), derived from MSE, expresses the logarithmic ratio between the maximum possible signal intensity and the power of corrupting noise. While these metrics are computationally simple and widely used in image restoration tasks such as denoising or super-resolution, they correlate poorly with human visual perception and often fail to reflect structural or semantic integrity.

***SSIM / MS-SSIM / FSIM /*** ***IW-SSIM:*** The Structural Similarity Index (SSIM)[96] addresses some of the shortcomings of pixel-based metrics by incorporating human visual perception principles. It evaluates image similarity by analyzing luminance, contrast, and structural components within local regions. Multi-Scale SSIM (MS-SSIM)[97] extends this concept by integrating information across multiple scales, improving sensitivity to global structural consistency and content variations. The Feature Similarity Index (FSIM)[98] enhances structural assessment by leveraging phase congruency and gradient magnitude, which are particularly effective for detecting edges and preserving texture, both of which are critical in medical imaging. Information Content Weighted SSIM (IW-SSIM)[99] incorporates a weighting scheme based on local information content, assigning greater importance to regions with higher perceptual or diagnostic value. This enhances its ability to assess clinically relevant areas such as lesions or organ boundaries, addressing the uniform sensitivity limitation of standard ssim. These metrics better approximate human perception compared to traditional error-based metrics, but their focus on local structure makes them less effective in detecting high-level semantic inconsistencies or anatomical implausibility.

***VIF / UQI / CACI****:* Visual Information Fidelity (VIF)[407] evaluates image quality using an information-theoretic model of the human visual system, estimating how much visual information is preserved in a distorted image relative to a reference. This offers a principled assessment of signal degradation, though at higher computational cost. Universal Quality Index (UQI)[408], in contrast, is a statistically-driven metric that integrates luminance, contrast, and structural similarity into a unified score. Both metrics provide a broader perspective on image degradation than pixel-based measures but are limited in capturing high-level semantic consistency. Complementing these, the Conservation and Correction Index (CACI)[100] jointly assesses structural preservation in healthy regions and effective correction in pathological areas by combining SSIM with lesion-based segmentation masks. These metric bridges low-level fidelity and task-specific evaluation, offering greater relevance in medical imaging scenarios that require both anatomical accuracy and pathology removal.

These metrics provide a useful assessment of structural integrity and visual fidelity, and are particularly effective in tasks such as image denoising, reconstruction, and compression. However, their reliance on low-level features limits their ability to detect high-level semantic inconsistencies, anatomical implausibility, or clinical irrelevance—factors that are critical in evaluating the diagnostic utility of generative models in medical imaging.

- 1. **Mid-Level Evaluation: Feature and Distribution Consistency**

Mid-level evaluation metrics assess the similarity between real and generated samples in a high-dimensional feature space, offering a more semantically informed and perceptually grounded perspective than pixel-based metrics. These methods typically rely on pretrained deep neural networks or kernel-based statistical comparisons to capture global distribution alignment, image realism, and sample diversity. They are especially valuable in tasks such as modality translation, unpaired image synthesis, and dynamic image generation.

***FID / KID / MMD / IS:*** Fréchet Inception Distance (FID)[101] compares real and generated image distributions by extracting deep features from a pretrained Inception-V3 network and modeling them as multivariate gaussians. It computes the wasserstein-2 distance[409] between these distributions, capturing both image quality and diversity. Kernel Inception Distance (KID)[102] improves robustness by employing polynomial-kernel-based maximum mean discrepancy, which avoids Gaussian assumptions and performs effectively even with limited data. Maximum Mean Discrepancy (MMD)[103] is a mid-level statistical metric that measures the distance between the feature distributions of real and generated images. It is commonly used as an alternative to FID when Gaussian assumptions are not desired, and forms the basis of kernel-based metrics such as KID. Inception Score (IS)[410] assesses image realism based on classification confidence and output diversity, calculated via Kullback–Leibler divergence. However, IS only evaluates individual images and depends heavily on the domain of the pretrained classifier. While these metrics are widely used, their reliance on natural-image-trained backbones (e.g., Inception V3 or VGG16), limits their sensitivity to domain-specific structures and pathological variation, making them best suited for preliminary quality screening.

***LPIPS / CLIP Similarity*** ***/ MedCLIP-score:*** To assess and mitigate hallucinations in generative medical imaging, where synthesized outputs may introduce or omit clinically important features, metrics such as Learned Perceptual Image Patch Similarity (LPIPS)[104], CLIP Similarity[105], and MedCLIP-score[106] have become essential tools. LPIPS evaluates perceptual similarity by comparing deep feature activations from pretrained convolutional networks, capturing structural coherence and visual realism across tasks such as anatomical inpainting and cross-modality synthesis. In comparison, CLIP Similarity and its domain-adapted variant MedCLIP-score embed both the generated image and its associated text prompt into a shared vision-language space using transformer-based encoders. The cosine similarity between the resulting embeddings reflects semantic alignment, which is particularly useful for detecting hallucinations when the generated content deviates from expected anatomical structures, pathological patterns, or spatial context. These metrics extend evaluation beyond pixel-level fidelity by capturing both visual and conceptual consistency. However, their effectiveness may depend on the domain specificity and robustness of the underlying pretrained models.

***RQI / AHI / BmU-I / BmU-V:*** To assess hallucination and semantic consistency in medical image generation, a set of complementary metrics has been proposed. The Restoration Quality Index (RQI)[100] evaluates low-level perceptual fidelity using LPIPS, measuring how closely restored images resemble healthy references in visual appearance. The Anomaly-to-Healthy Index (AHI)[100] assesses whether pathological images, once restored, align with the distribution of healthy data. Based on FID, AHI provides a statistical view of normalization effectiveness. At the semantic level, the Biomedical Understanding (BmU)[107] framework introduces a hallucination-aware evaluation strategy tailored for medical imaging. BmU-I applies a large language model to generate textual descriptions from image sequences, which are then compared with the original prompts using BERT-based[411] embedding similarity. This captures whether the generated content aligns with intended diagnostic meaning. BmU-V extends this approach to dynamic imaging by using video-language models such as Video-LLaMA[412] to evaluate consistency between temporal visual content and associated text. It is particularly suited to applications like cine MRI and surgical video generation. Collectively, these metrics provide a multi-level evaluation framework covering perceptual quality, statistical alignment, and semantic fidelity, enabling robust assessment of generative model reliability in clinical contexts.

***FVD / KVD / FVMD / VBench:*** Fréchet Video Distance (FVD)[108] extends FID to video by using 3D convolutional networks (e.g., I3D[413]) to extract spatiotemporal features, allowing the evaluation of both visual fidelity and motion consistency. Kernel Video Distance (KVD)[414] evaluates the similarity between real and generated videos by comparing their spatiotemporal features using a kernel-based approach. Fréchet Video Motion Distance (FVMD)[109] targets dynamic realism by tracking key points across frames, analyzing velocity and acceleration histograms, and comparing them using Fréchet distance. This is especially important in cine MRI, cardiac imaging, and moving organ simulations. VBench[415] offers a modular framework for video assessment, integrating pretrained models such as RAFT (motion)[416], MUSIQ (frame quality)[417], and ViCLIP (semantics)[418] with heuristic algorithms to evaluate spatial consistency, motion smoothness, dynamic degree, and temporal stylistic consistency. These metrics are crucial for dynamic imaging tasks but computationally intensive and often trained on non-medical data, potentially limiting clinical interpretability.

Mid-level evaluations bridge the gap between low-level pixel fidelity and high-level clinical relevance. While they offer more meaningful insight into perceptual and statistical realism, their effectiveness is still influenced by the domain alignment of the pretrained models used and the complexity of the evaluation task. In practice, these metrics are best used in combination with both low- and high-level assessments to ensure comprehensive validation of generative performance.

- 1. **High-Level Evaluation:** **Expert and Clinical Assessment**

High-level evaluation constitutes the final and most clinically significant stage in the assessment hierarchy of generative models for medical imaging. Unlike low- and mid-level metrics that emphasize pixel-level fidelity or feature-space alignment, this stage focuses on practical usability in real-world clinical workflows. It seeks to determine whether synthetic images can support essential medical tasks such as diagnosis, treatment planning, or disease monitoring. To achieve this, high-level evaluation combines two complementary approaches: expert assessment rooted in subjective diagnostic judgment and clinical validation based on downstream task performance, providing a comprehensive understanding of the model’s clinical utility.

- - 1. **Expert Evaluation and Clinical Feedback**

While quantitative metrics provide a baseline assessment of image fidelity and performance in downstream tasks, expert evaluation by radiologists and clinical specialists remains essential for determining the true clinical viability of generated medical images. These assessments offer nuanced insights into aspects such as diagnostic realism, anatomical consistency, and semantic plausibility, which are often beyond the reach of purely algorithmic evaluation. In these settings, clinicians are typically asked to blindly compare synthetic and real images, identify anatomical inaccuracies, assess lesion visibility, and judge whether an image meets diagnostic standards. This human-in-the-loop[419] approach helps uncover subtle issues such as unnatural tissue textures, inconsistent lesion morphology, or implausible anatomical deformations, which may not be penalized by numerical similarity metrics. Recent real-world evaluations have shown that expert-involved assessments provide not only qualitative feedback but also quantitative evidence of clinical relevance, demonstrating that human-in-the-loop evaluation effectively bridges algorithmic performance with clinical applicability.

A representative example of such real-world evaluation is the GenerateCT study[180], a blinded evaluation was conducted in which two radiologists with 4 and 11 years of experience, respectively, independently reviewed a total of 200 chest CT volumes. The dataset included 100 real and 100 synthetic cases. They were tasked with identifying whether each image was real or synthetic, and assessing the semantic alignment between the CT volumes and their corresponding radiological prompts. The first radiologist correctly identified 74% of real cases and 59% of synthetic ones, while the second achieved 71% and 64%, respectively. Both experts correctly recognized more than half of the real and synthetic images, indicating that the generated CT volumes exhibited high visual realism and diagnostic plausibility. Furthermore, 70% of synthetic volumes were judged to accurately match the prompts. This evaluation design highlights the ability of experts to uncover diagnostic realism and semantic integrity that are not captured by automated metrics. A more comprehensive expert-involved framework was proposed in MINIM [18], a generalist medical image-text generative model​. In this study, clinicians were asked to evaluate synthetic images across four modalities (OCT, fundus, chest CT, and chest X-ray) by scoring them on a 1–3 scale: 1). to low quality, 2).to high quality but misaligned with the prompt, 3).to high quality and semantically aligned. Three rounds of evaluations were conducted. Initially, only 70.75% of images received a top score of 3. These clinician ratings were then used to train a reinforcement learning from human feedback (RLHF) reward model, which guided the model to self-improve in a closed-loop fashion. By the third round, 89.25% of synthetic images were rated 3, with significant improvements across all modalities (e.g., chest CT improved from 61% to 83%). This study exemplifies a dynamic and iterative human-in-the-loop framework where expert judgment not only evaluates but actively enhances the generative model’s performance. These studies not only validate the perceptual and semantic quality of synthetic data but also demonstrate how iterative human feedback loops can lead to measurable gains in diagnostic trustworthiness, thereby bridging the gap between algorithmic realism and clinical utility.

- - 1. **Clinical Validation via Downstream Tasks**

Clinical evaluation focuses on verifying whether synthetic images retain essential anatomical and pathological features required for medical decision-making. A common strategy is to apply the generated data to downstream tasks such as segmentation, classification, or regression, and assess how well the models perform when trained or tested using the synthetic inputs. These tasks serve as indirect clinical proxies, reflecting whether the generated content contains sufficient and relevant clinical information.

***Segmentation tasks*** are used to assess whether synthetic images preserve anatomical fidelity that is essential for clinical interpretation. By training or evaluating segmentation models on synthetic data and comparing the results to real-image baselines, researchers can determine if the generated images maintain clear structural boundaries. Metrics such as the dice coefficient and Intersection over Union (IoU) quantify spatial alignment between predictions and expert annotations. For example, in the Med-DDPM[110] study, synthetic multi-modal MRIs were used to train a tumor segmentation under varying ratios of real and generated data. When synthetic data were incorporated into training, segmentation performance improved progressively, achieving up to a 2.2% increase in dice score compared with the real-image baseline. These results indicate that incorporating synthetic MR images can enhance segmentation accuracy and support clinical interpretability, particularly in data-limited scenarios.

***Classification tasks*** evaluate whether synthetic images encode meaningful diagnostic signals that allow models to distinguish between disease subtypes or molecular phenotypes. Metrics such as accuracy, area under the curve (AUC), and sensitivity are typically used to quantify classification performance. In the MINIM framework[18], which showed that synthetic data can enhance downstream diagnostic accuracy. Incorporating synthetic breast cancer images into the training set increased HER2-positive tumor classification accuracy from 79.2% to 94.0%. For EGFR mutation prediction in lung cancer, adding synthetic images improved average top-1 accuracy from 81.5% to 95.4%. These gains were most notable in underrepresented subgroups, suggesting that the generated data not only preserved diagnostic features but also improved model generalization in clinically relevant settings.

***Regression tasks*** help assess whether synthetic images retain continuous clinical attributes relevant to physiological function or disease progression. This type of evaluation is especially important in dynamic imaging, where visual plausibility must be supported by measurable clinical signals. In a study[63], synthetic echocardiogram sequences were used to augment training data for left ventricular ejection fraction prediction, a key indicator of cardiac performance. A regression model trained on 790 real samples achieved an R² score of 56%. When the training set was rebalanced by incorporating approximately 50% synthetic data to compensate for underrepresented LVEF ranges, the model’s performance improved to an R² of 59% on a balanced validation set. These findings indicate that the generated images preserved physiologically meaningful variation and supported more accurate functional prediction, particularly in limited-data scenarios.

These task-driven evaluations demonstrate that synthetic medical images can effectively support a range of clinically relevant applications. By preserving structural, diagnostic, and physiological features, generated data enable reliable model performance across segmentation, classification, and regression tasks. Such results highlight the potential of generative models to enhance clinical workflows, particularly in scenarios with limited or imbalanced data availability.

**Supplementary References**

[131] Akpinar M H, Sengur A, Salvi M, Seoni S, Faust O, Mir H, Molinari F, and Acharya U R, “Synthetic data generation via generative adversarial networks in healthcare: a systematic review of image- and signal-based studies,” IEEE Open J. Eng. Med. Biol., 2024, doi: 10.1109/OJEMB.2024.3508472.

[132] Alamir M and Alghamdi M, “The role of generative adversarial network in medical image analysis: an In-depth survey,” *ACM Comput. Surv.*, vol. 55, no. 5, pp. 1–36, May 2023, doi: 10.1145/3527849.

[133] Pan Z, Yu W, Wang B, Xie H, Sheng V S, Lei J, and Kwong S, “Loss functions of generative adversarial networks (GANs): opportunities and challenges,” *IEE Trans. Emerg. Topics Comput. Intell.*, vol. 4, no. 4, pp. 500–522, 2020, doi: 10.1109/TETCI.2020.2991774.

[134] Shokraei Fard A, Reutens D C, and Vegh V, “From CNNs to GANs for cross-modality medical image estimation,” *Comput. Biol. Med.*, vol. 146, p. 105556, Jul. 2022, doi: 10.1016/j.compbiomed.2022.105556.

[135] Apostolopoulos I D, Papathanasiou N D, Apostolopoulos D J, and Panayiotakis G S, “Applications of generative adversarial networks (GANs) in positron emission tomography (PET) imaging: a review,” *Eur. J. Nucl. Med. Mol. Imaging*, vol. 49, no. 11, pp. 3717–3739, Sep. 2022, doi: 10.1007/s00259-022-05805-w.

[136] Singh N K and Raza K, “Medical image generation using generative adversarial networks: a review,” in *Health Informatics: A Computational Perspective in Healthcare*, vol. 932, Singapore: Springer Singapore, 2021, pp. 77–96. doi: 10.1007/978-981-15-9735-0_5.

[137] Rais K, Amroune M, Benmachiche A, and Haouam M Y, “Exploring variational autoencoders for medical image generation: A comprehensive study,” Nov. 11, 2024, *arXiv*: arXiv:2411.07348. doi: 10.48550/arXiv.2411.07348.

[138] Ehrhardt J and Wilms M, “Autoencoders and variational autoencoders in medical image analysis,” in *Biomedical Image Synthesis and Simulation*, Elsevier, 2022, pp. 129–162. Accessed: Mar. 13, 2025. [Online]. Available: https://www.sciencedirect.com/science/article/pii/B9780128243497000153

[139] Shi Y, Abulizi A, Wang H, Feng K, Abudukelimu N, Su Y, and Abudukelimu H, “Diffusion models for medical image computing: a survey,” *Tsinghua Sci. Technol.*, vol. 30, no. 1, pp. 357–383, 2024, doi: 10.26599/TST.2024.9010047.

[140] Alimisis P, Mademlis I, Radoglou-Grammatikis P, Sarigiannidis P, and Papadopoulos G Th, “Advances in diffusion models for image data augmentation: a review of methods, models, evaluation metrics and future research directions,” *Artif. Intell. Rev.*, vol. 58, no. 4, p. 112, Jan. 2025, doi: 10.1007/s10462-025-11116-x.

[141] Fan Y, Liao H, Huang S, Luo Y, Fu H, and Qi H, “A survey of emerging applications of diffusion probabilistic models in MRI,” *Meta-radiol.*, vol. 2, no. 2, p. 100082, 2024, doi: 10.1016/j.metrad.2024.100082.

[142] Hein D, Bozorgpour A, Merhof D, and Wang G, “Physics-inspired generative models in medical imaging,” *Annu. Rev. Biomed. Eng.*, vol. 27, no. 1, pp. 499–525, May 2025, doi: 10.1146/annurev-bioeng-102723-013922.

[143] Kazerouni A, Aghdam E K, Heidari M, Azad R, Fayyaz M, Hacihaliloglu I, and Merhof D, “Diffusion models in medical imaging: a comprehensive survey,” *Med. Image Anal.*, vol. 88, p. 102846, 2023, doi: 10.1016/j.media.2023.102846.

[144] He K, Gan C, Li Z, Rekik I, Yin Z, Ji W, Gao Y, Wang Q, Zhang J, and Shen D, “Transformers in medical image analysis,” *Intell. Med.*, vol. 3, no. 1, pp. 59–78, 2023, doi: 10.1016/j.imed.2022.07.002.

[145] Heidari M, Kolahi S G, Karimijafarbigloo S, Azad B, Bozorgpour A, Hatami S, Azad R, Diba A, Bagci U, Merhof D, *et al.*, “Computation-efficient era: a comprehensive survey of state space models in medical image analysis,” Jun. 05, 2024, *arXiv*: arXiv:2406.03430. doi: 10.48550/arXiv.2406.03430.

[146] Xiong J, Liu G, Huang L, Wu C, Wu T, Mu Y, Yao Y, Shen H, Wan Z, Huang J, *et al.*, “Autoregressive models in vision: a survey,” Nov. 08, 2024, *arXiv*: arXiv:2411.05902. doi: 10.48550/arXiv.2411.05902.

[147] Huang J, Fang Y, Nan Y, Wu H, Wu Y, Gao Z, Li Y, Wang Z, Lio P, Rueckert D, *et al.*, “Data and physics driven learning models for fast MRI -- fundamentals and methodologies from CNN, GAN to attention and transformers,” Apr. 01, 2022, *arXiv*: arXiv:2204.01706. doi: 10.48550/arXiv.2204.01706.

[148] Christensen M, Vukadinovic M, Yuan N, and Ouyang D, “Vision–language foundation model for echocardiogram interpretation,” *Nat. Med.*, vol. 30, no. 5, pp. 1481–1488, 2024, doi: 10.1038/s41591-024-02959-y.

[149] Kebaili A, Lapuyade-Lahorgue J, and Ruan S, “Deep learning approaches for data augmentation in medical imaging: A review,” *J. Imaging*, vol. 9, no. 4, p. 81, 2023, doi: 10.3390/jimaging9040081.

[150] Cossio M, “Augmenting medical imaging: a comprehensive catalogue of 65 techniques for enhanced data analysis,” Mar. 02, 2023, *arXiv*: arXiv:2303.01178. doi: 10.48550/arXiv.2303.01178.

[151] Pezoulas V C, Zaridis D I, Mylona E, Androutsos C, Apostolidis K, Tachos N S, and Fotiadis D I, “Synthetic data generation methods in healthcare: a review on open-source tools and methods,” *Comput. Struct. Biotechnol. J.*, 2024, doi: 10.1016/j.csbj.2024.07.005.

[152] Wang T, Lei Y, Fu Y, Curran W J, Liu T, and Yang X, “Medical imaging synthesis using deep learning and its clinical applications: a review,” Apr. 21, 2020, *arXiv*: arXiv:2004.10322. doi: 10.48550/arXiv.2004.10322.

[153] Liu Y, Dwivedi G, Boussaid F, and Bennamoun M, “3D brain and heart volume generative models: a survey,” *ACM Comput. Surv.*, vol. 56, no. 6, pp. 1–37, Jun. 2024, doi: 10.1145/3638044.

[154] Spadea M F, Maspero M, Zaffino P, and Seco J, “Deep learning based synthetic-CT generation in radiotherapy and PET: a review,” *Med. Phys.*, vol. 48, no. 11, pp. 6537–6566, 2021, doi: 10.1002/mp.15150.

[155] Lombardi A F, Ma Y-J, Jang H, Jerban S, Du J, Chang E Y, and Chung C B, “Synthetic CT in musculoskeletal disorders: a systematic review,” *Invest. Radiol.*, vol. 58, no. 1, pp. 43–59, 2023, doi: 10.1097/RLI.0000000000000916.

[156] Manjooran G P, Malakkaran A J, Joseph A, Babu H M, and Meharban M S, “A review on cross-modality synthesis from MRI to PET,” in *ICSCCC - Int. Conf. Secur. Cyber Comput. Commun.*, Institute of Electrical and Electronics Engineers Inc., 2021, pp. 126–131. doi: 10.1109/ICSCCC51823.2021.9478170.

[157] Boulanger M, Nunes J-C, Chourak H, Largent A, Tahri S, Acosta O, De Crevoisier R, Lafond C, and Barateau A, “Deep learning methods to generate synthetic CT from MRI in radiotherapy: a literature review,” *Physica Med.*, vol. 89, pp. 265–281, 2021, doi: 10.1016/j.ejmp.2021.07.027.

[158] Wang S, Xiao T, Liu Q, and Zheng H, “Deep learning for fast MR imaging: A review for learning reconstruction from incomplete k-space data,” *Biomed. Signal Process. Control*, vol. 68, p. 102579, Jul. 2021, doi: 10.1016/j.bspc.2021.102579.

[159] Zeng G, Guo Y, Zhan J, Wang Z, Lai Z, Du X, Qu X, and Guo D, “A review on deep learning MRI reconstruction without fully sampled k-space,” *BMC Med. Imaging*, vol. 21, no. 1, p. 195, Dec. 2021, doi: 10.1186/s12880-021-00727-9.

[160] Chen Y, Schönlieb C-B, Liò P, Leiner T, Dragotti P L, Wang G, Rueckert D, Firmin D, and Yang G, “AI-based reconstruction for fast MRI—a systematic review and meta-analysis,” *Proceedings of the IEEE*, vol. 110, no. 2, pp. 224–245, 2022.

[161] “Deep learning techniques in PET/CT imaging: A comprehensive review from sinogram to image space,” *Comput. Methods Programs Biomed.*, vol. 243, p. 107880, Jan. 2024, doi: 10.1016/j.cmpb.2023.107880.

[162] Samala R K, Drukker K, Shukla-Dave A, Chan H-P, Sahiner B, Petrick N, Greenspan H, Mahmood U, Summers R M, and Tourassi G, “AI and machine learning in medical imaging: key points from development to translation,” *BJR| Artif. Intell.*, vol. 1, no. 1, p. ubae006, 2024.

[163] Chaddad A, Hu Y, Wu Y, Wen B, and Kateb R, “Generalizable and explainable deep learning for medical image computing: an overview,” *Curr. Opin. Biomed. Eng.*, vol. 33, 2025, doi: 10.1016/j.cobme.2024.100567.

[164] Dimitriadis A, Trivizakis E, Papanikolaou N, Tsiknakis M, and Marias K, “Enhancing cancer differentiation with synthetic MRI examinations via generative models: a systematic review,” *Insights into Imaging*, vol. 13, no. 1, 2022, doi: 10.1186/s13244-022-01315-3.

[165] Koohi-Moghadam M and Bae K T, “Generative AI in medical imaging: Applications, challenges, and ethics,” *J. Med. Syst.*, vol. 47, no. 1, p. 94, Aug. 2023, doi: 10.1007/s10916-023-01987-4.

[166] Radford A, Metz L, and Chintala S, “Unsupervised representation learning with deep convolutional generative adversarial networks,” Jan. 07, 2016, *arXiv*: arXiv:1511.06434. doi: 10.48550/arXiv.1511.06434.

[167] Zhu J-Y, Park T, Isola P, and Efros A A, “Unpaired image-to-image translation using cycle-consistent adversarial networks,” in *Proceedings of the IEEE International Conference on Computer Vision*, 2017, pp. 2223–2232. Accessed: Mar. 16, 2025. [Online]. Available: http://openaccess.thecvf.com/content_iccv_2017/html/Zhu_Unpaired_Image-To-Image_Translation_ICCV_2017_paper.html

[168] Kang S K, An H J, Jin H, Kim J-I, Chie E K, Park J M, and Lee J S, “Synthetic CT generation from weakly paired MR images using cycle-consistent GAN for MR-guided radiotherapy,” *Biomed. Eng. Lett.*, vol. 11, no. 3, pp. 263–271, 2021, doi: 10.1007/s13534-021-00195-8.

[169] Karras T, Laine S, and Aila T, “A style-based generator architecture for generative adversarial networks,” in *Proceedings of the IEEE/CVF Conference on Computer Vision and Pattern Recognition*, 2019, pp. 4401–4410. Accessed: Sep. 09, 2025. [Online]. Available: http://openaccess.thecvf.com/content_CVPR_2019/html/Karras_A_Style-Based_Generator_Architecture_for_Generative_Adversarial_Networks_CVPR_2019_paper.html

[170] Krishna A and Mueller K, “Medical (CT) image generation with style,” in *Proc SPIE Int Soc Opt Eng*, Matej S. and Metzler S.D., Eds., SPIE, 2019. doi: 10.1117/12.2534903.

[171] Lai M, Marzi C, Mascalchi M, and Diciotti S, “Brain MRI synthesis using Stylegan2-ADA,” in *IEEE Comput. Soc. Conf. Comput. Vis. Pattern Recogn.*, IEEE Computer Society, 2024. doi: 10.1109/ISBI56570.2024.10635279.

[172] Sundar V K, Ramakrishna S, Rahiminasab Z, Easwaran A, and Dubey A, “Out-of-distribution detection in multi-label datasets using latent space of β-VAE,” in *2020 IEEE Security and Privacy Workshops (SPW)*, May 2020, pp. 250–255. doi: 10.1109/SPW50608.2020.00057.

[173] Loizillon S, Jacob Y, Maire A, Dormont D, Colliot O, and Burgos N, “Detecting brain anomalies in clinical routine with the β-VAE: feasibility study on age-related white matter hyperintensities,” in *Medical Imaging with Deep Learning - MIDL 2024*, Paris, France, Jul. 2024. Accessed: Mar. 16, 2025. [Online]. Available: https://hal.science/hal-04674025

[174] Pesteie M, Abolmaesumi P, and Rohling R N, “Adaptive augmentation of medical data using independently conditional variational auto-encoders,” *IEEE Trans. Med. Imag.*, vol. 38, no. 12, pp. 2807–2820, 2019, doi: 10.1109/TMI.2019.2914656.

[175] Van Den Oord A and Vinyals O, “Neural discrete representation learning,” *Adv. Neural Inf. Process. Syst.*, vol. 30, 2017, Accessed: Mar. 16, 2025. [Online]. Available: https://proceedings.neurips.cc/paper/2017/hash/7a98af17e63a0ac09ce2e96d03992fbc-Abstract.html

[176] Ramanathan S and Ramasundaram M, “Vector quantized convolutional autoencoder network for LDCT image reconstruction with hybrid loss,” *SN Comput. Sci.*, vol. 5, no. 1, p. 2, Nov. 2023, doi: 10.1007/s42979-023-02295-x.

[177] Zhao A, Xu M, Shahin A H, Wuyts W, Jones M G, Jacob J, and Alexander D C, “4D VQ-GAN: synthesising medical scans at any time point for personalised disease progression modelling of idiopathic pulmonary fibrosis,” Feb. 08, 2025, *arXiv*: arXiv:2502.05713. doi: 10.48550/arXiv.2502.05713.

[178] Ibrahim B I, Nicolae D C, Khan A, Ali S I, and Khattak A, “VAE-GAN based zero-shot outlier detection,” in *Proceedings of the 2020 4th International Symposium on Computer Science and Intelligent Control*, Newcastle upon Tyne United Kingdom: ACM, Nov. 2020, pp. 1–5. doi: 10.1145/3440084.3441180.

[179] Volokitin A, Erdil E, Karani N, Tezcan K C, Chen X, Van Gool L, and Konukoglu E, “Modelling the distribution of 3D brain MRI using a 2D slice VAE,” in *Medical Image Computing and Computer Assisted Intervention – MICCAI 2020*, vol. 12267, Cham: Springer International Publishing, 2020, pp. 657–666. doi: 10.1007/978-3-030-59728-3_64.

[180] Hamamci I E, Er S, Sekuboyina A, Simsar E, Tezcan A, Simsek A G, Esirgun S N, Almas F, Doğan I, Dasdelen M F, *et al.*, “GenerateCT: text-conditional generation of 3D chest CT volumes,” in *Computer Vision – ECCV 2024*, vol. 15137, Cham: Springer Nature Switzerland, 2025, pp. 126–143. doi: 10.1007/978-3-031-72986-7_8.

[181] Huang P, Guo B, Liang S, Fu J, Wang Y, and Guo Y, “Diff-CXR: Report-to-CXR generation through a disease-knowledge enhanced diffusion model,” Oct. 26, 2024, *arXiv*: arXiv:2410.20165. doi: 10.48550/arXiv.2410.20165.

[182] Arslan F, Kabas B, Dalmaz O, Ozbey M, and Çukur T, “Self-consistent recursive diffusion bridge for medical image translation,” *Med. Image Anal.*, vol. 106, p. 103747, 2025, doi: 10.1016/j.media.2025.103747.

[183] Daum D, Osuala R, Riess A, Kaissis G, Schnabel J A, and Di Folco M, “On differentially private 3D medical image synthesis with controllable latent diffusion models,” in *Deep Generative Models*, vol. 15224, Cham: Springer Nature Switzerland, 2025, pp. 139–149. doi: 10.1007/978-3-031-72744-3_14.

[184] Sanderson D, Olmos P M, Del Cerro C F, Desco M, and Abella M, “Diffusion X-ray image denoising,” in *Medical imaging with deep learning*, 2024. Accessed: Mar. 17, 2025. [Online]. Available: https://www.researchgate.net/profile/Daniel-Sanderson-2/publication/382462715_Diffusion_X-ray_image_denoising/links/669f5e5a8be3067b4b110ec4/Diffusion-X-ray-image-denoising.pdf

[185] Xiang T, Yurt M, Syed A B, Setsompop K, and Chaudhari A, “DDM$^2$: self-supervised diffusion MRI denoising with generative diffusion models,” Feb. 06, 2023, *arXiv*: arXiv:2302.03018. doi: 10.48550/arXiv.2302.03018.

[186] Saharia C, Ho J, Chan W, Salimans T, Fleet D J, and Norouzi M, “Image super-resolution via iterative refinement,” *IEEE Trans. Pattern Anal. Mach. Intell.*, vol. 45, no. 4, pp. 4713–4726, 2022.

[187] Xia B, Zhang Y, Wang S, Wang Y, Wu X, Tian Y, Yang W, and Van Gool L, “Diffir: efficient diffusion model for image restoration,” in *Proceedings of the IEEE/CVF International Conference on Computer Vision*, 2023, pp. 13095–13105. Accessed: Mar. 17, 2025. [Online]. Available: http://openaccess.thecvf.com/content/ICCV2023/html/Xia_DiffIR_Efficient_Diffusion_Model_for_Image_Restoration_ICCV_2023_paper.html

[188] Dorjsembe Z, Odonchimed S, and Xiao F, “Three-dimensional medical image synthesis with denoising diffusion probabilistic models,” in *Medical Imaging with Deep Learning*, 2022. Accessed: Mar. 17, 2025. [Online]. Available: https://openreview.net/forum?id=Oz7lKWVh45H

[189] Xu K, Lu S, Huang B, Wu W, and Liu Q, “Stage-by-stage wavelet optimization refinement diffusion model for sparse-view CT reconstruction,” *IEEE Trans. Med. Imag.*, vol. 43, no. 10, pp. 3412–3424, Oct. 2024, doi: 10.1109/TMI.2024.3355455.

[190] Xie T, Cui Z-X, Luo C, Wang H, Liu C, Zhang Y, Wang X, Zhu Y, Chen G, Liang D, *et al.*, “Joint diffusion: mutual consistency-driven diffusion model for PET-MRI co-reconstruction,” *Phys. Med. Biol.*, vol. 69, no. 15, p. 155019, Jul. 2024, doi: 10.1088/1361-6560/ad6117.

[191] Qiu S, Pan S, Liu Y, Zhao L, Xu J, Liu Q, Chen T, Chen E Z, Chen X, and Sun S, “Spatiotemporal diffusion model with paired sampling for accelerated cardiac cine MRI,” Mar. 13, 2024, *arXiv*: arXiv:2403.08758. doi: 10.48550/arXiv.2403.08758.

[192] Guo Z, Liu J, Wang Y, Chen M, Wang D, Xu D, and Cheng J, “Diffusion models in bioinformatics and computational biology,” *Nat. Rev. Bioeng.*, vol. 2, no. 2, pp. 136–154, 2024, doi: 10.1038/s44222-023-00114-9.

[193] Gregor K, Danihelka I, Mnih A, Blundell C, and Wierstra D, “Deep autoregressive networks,” in *International Conference on Machine Learning*, PMLR, 2014, pp. 1242–1250. Accessed: Mar. 16, 2025. [Online]. Available: http://proceedings.mlr.press/v32/gregor14.html

[194] Gao Y, Zhou M, Liu D, Yan Z, Zhang S, and Metaxas D N, “A data-scalable transformer for medical image segmentation: architecture, model efficiency, and benchmark,” Apr. 05, 2023, *arXiv*: arXiv:2203.00131. doi: 10.48550/arXiv.2203.00131.

[195] Zhao X, Yang T, Li B, and Zhang X, “SwinGAN: a dual-domain swin transformer-based generative adversarial network for MRI reconstruction,” *Comput. Biol. Med.*, vol. 153, 2023, doi: 10.1016/j.compbiomed.2022.106513.

[196] Kottu J, “A vision transformer-driven method for generating medical reports based on x-ray radiology,” Master thesis, California State University, Sacramento, 2024. Accessed: Mar. 17, 2025. [Online]. Available: https://search.proquest.com/openview/7dc270e1d91bc26b220c8be408b726a9/1?pq-origsite=gscholar&cbl=18750&diss=y

[197] Adams L C, Busch F, Truhn D, Makowski M R, Aerts H J W L, and Bressem K K, “What does DALL-E 2 know about radiology?,” *J. Med. Internet Res.*, vol. 25, no. 1, p. e43110, Mar. 2023, doi: 10.2196/43110.

[198] Yue Y and Li Z, “MedMamba: vision mamba for medical image classification,” Sep. 29, 2024, *arXiv*: arXiv:2403.03849. doi: 10.48550/arXiv.2403.03849.

[199] Ju Z and Zhou W, “VM-DDPM: vision mamba diffusion for medical image synthesis,” May 09, 2024, *arXiv*: arXiv:2405.05667. doi: 10.48550/arXiv.2405.05667.

[200] Li K, Li X, Wang Y, He Y, Wang Y, Wang L, and Qiao Y, “VideoMamba: state space model for efficient video understanding,” in *Computer Vision – ECCV 2024*, vol. 15084, Cham: Springer Nature Switzerland, 2025, pp. 237–255. doi: 10.1007/978-3-031-73347-5_14.

[201] Chen Z, Wang S, Yan D, and Li Y, “A spatio-temporl deepfake video detection method based on TimeSformer-CNN,” in *2024 Third International Conference on Distributed Computing and Electrical Circuits and Electronics (ICDCECE)*, IEEE, 2024, pp. 1–6. Accessed: Mar. 17, 2025. [Online]. Available: https://ieeexplore.ieee.org/abstract/document/10549278/

[202] Behrouz A, Santacatterina M, and Zabih R, “MambaMixer: efficient selective state space models with dual token and channel selection,” Jul. 23, 2024, *arXiv*: arXiv:2403.19888. doi: 10.48550/arXiv.2403.19888.

[203] Luo G, Huang S, and Uecker M, “Autoregressive image diffusion: generation of image sequence and application in mri,” *Adv. Neural Inf. Process. Syst.*, vol. 37, pp. 129094–129119, 2024.

[204] Kabas B, Arslan F, Nezhad V A, Ozturk S, Saritas E U, and Çukur T, “Physics-driven autoregressive state space models for medical image reconstruction,” Dec. 12, 2024, *arXiv*: arXiv:2412.09331. doi: 10.48550/arXiv.2412.09331.

[205] Park J, Park J, Xiong Z, Lee N, Cho J, Oymak S, Lee K, and Papailiopoulos D, “Can mamba learn how to learn? A comparative study on In-context learning tasks,” Apr. 25, 2024, *arXiv*: arXiv:2402.04248. doi: 10.48550/arXiv.2402.04248.

[206] Cardoso M J, Li W, Brown R, Ma N, Kerfoot E, Wang Y, Murrey B, Myronenko A, Zhao C, Yang D, *et al.*, “MONAI: an open-source framework for deep learning in healthcare,” Nov. 04, 2022, *arXiv*: arXiv:2211.02701. doi: 10.48550/arXiv.2211.02701.

[207] He K, Chen X, Xie S, Li Y, Dollár P, and Girshick R, “Masked autoencoders are scalable vision learners,” in *Proceedings of the IEEE/CVF Conference on Computer Vision and Pattern Recognition*, 2022, pp. 16000–16009. Accessed: Jul. 11, 2025. [Online]. Available: https://openaccess.thecvf.com/content/CVPR2022/html/He_Masked_Autoencoders_Are_Scalable_Vision_Learners_CVPR_2022_paper

[208] Reichenpfader D, Müller H, and Denecke K, “A scoping review of large language model based approaches for information extraction from radiology reports,” *npj Digital Med.*, vol. 7, no. 1, p. 222, 2024, doi: 10.1038/s41746-024-01219-0.

[209] Thirunavukarasu A J, Ting D S J, Elangovan K, Gutierrez L, Tan T F, and Ting D S W, “Large language models in medicine,” *Nat. Med.*, vol. 29, no. 8, pp. 1930–1940, 2023, doi: 10.1038/s41591-023-02448-8.

[210] Liu F, Zhu T, Wu X, Yang B, You C, Wang C, Lu L, Liu Z, Zheng Y, and Sun X, “A medical multimodal large language model for future pandemics,” *npj Digital Med.*, vol. 6, no. 1, p. 226, 2023, doi: 10.1038/s41746-023-00952-2.

[211] Khattak M U, Kunhimon S, Naseer M, Khan S, and Khan F S, “UniMed-CLIP: towards a unified image-text pretraining paradigm for diverse medical imaging modalities,” Dec. 13, 2024, *arXiv*: arXiv:2412.10372. doi: 10.48550/arXiv.2412.10372.

[212] Shiri M, Beyan C, and Murino V, “MadCLIP: few-shot medical anomaly detection with CLIP,” in *Medical Image Computing and Computer Assisted Intervention – MICCAI 2025*, vol. 15965, Cham: Springer Nature Switzerland, 2026, pp. 416–426. doi: 10.1007/978-3-032-04978-0_40.

[213] Tiu E, Talius E, Patel P, Langlotz C P, Ng A Y, and Rajpurkar P, “Expert-level detection of pathologies from unannotated chest X-ray images via self-supervised learning,” *Nat. Biomed. Eng.*, vol. 6, no. 12, pp. 1399–1406, 2022, doi: 10.1038/s41551-022-00936-9.

[214] Moor M, Banerjee O, Abad Z S H, Krumholz H M, Leskovec J, Topol E J, and Rajpurkar P, “Foundation models for generalist medical artificial intelligence,” *Nature*, vol. 616, no. 7956, pp. 259–265, 2023, doi: 10.1038/s41586-023-05881-4.

[215] Wang G and Hu X, “Low-dose CT denoising using a progressive wasserstein generative adversarial network,” *Comput. Biol. Med.*, vol. 135, 2021, doi: 10.1016/j.compbiomed.2021.104625.

[216] Deng Z, Zhang W, Chen K, Zhou Y, Tian J, Quan G, and Zhao J, “TT U-net: temporal transformer U-net for motion artifact reduction using PAD (pseudo all-phase clinical-dataset) in cardiac CT,” *IEEE Trans. Med. Imag.*, vol. 42, no. 12, pp. 3805–3816, 2023, doi: 10.1109/TMI.2023.3310933.

[217] Öztürk Ş, Duran O C, and Çukur T, “DenoMamba: a fused state-space model for low-dose CT denoising,” Dec. 15, 2024, *arXiv*: arXiv:2409.13094. doi: 10.48550/arXiv.2409.13094.

[218] Hu Z, Li Y, Zou S, Xue H, Sang Z, Liu X, Yang Y, Zhu X, Liang D, and Zheng H, “Obtaining PET/CT images from non-attenuation corrected PET images in a single PET system using wasserstein generative adversarial networks,” *Phys. Med. Biol.*, vol. 65, no. 21, p. 215010, 2020, doi: 10.1088/1361-6560/aba5e9.

[219] Yang J, Sohn J H, Behr S C, Gullberg G T, and Seo Y, “CT-less direct correction of attenuation and scatter in the image space using deep learning for whole-body FDG PET: potential benefits and pitfalls,” *Radiol.: Artif. Intell.*, vol. 3, no. 2, p. e200137, Mar. 2021, doi: 10.1148/ryai.2020200137.

[220] Yu B and Gong K, “Adaptive whole-body pet image denoising using 3D diffusion models with controlnet,” in *2025 IEEE 22nd International Symposium on Biomedical Imaging (ISBI)*, IEEE, 2025, pp. 1–5. Accessed: Sep. 09, 2025. [Online]. Available: https://ieeexplore.ieee.org/abstract/document/10980878/

[221] Geng M, Meng X, Yu J, Zhu L, Jin L, Jiang Z, Qiu B, Li H, Kong H, Yuan J, *et al.*, “Content-noise complementary learning for medical image denoising,” *IEEE Trans. Med. Imag.*, vol. 41, no. 2, pp. 407–419, Feb. 2022, doi: 10.1109/TMI.2021.3113365.

[222] Lim A, Lo J, Wagner M W, Ertl-Wagner B, and Sussman D, “Motion artifact correction in fetal MRI based on a generative adversarial network method,” *Biomed. Signal Process. Control*, vol. 81, p. 104484, 2023, doi: 10.1016/j.bspc.2022.104484.

[223] Xu J, Zhou D, Hu L, Guo J, Yang F, Liu Z, Wang N, and Gao X, “Motion artifact removal in pixel-frequency domain via alternate masks and diffusion model,” in *Proceedings of the AAAI Conference on Artificial Intelligence*, 2025, pp. 8878–8886. Accessed: Sep. 09, 2025. [Online]. Available: https://ojs.aaai.org/index.php/AAAI/article/view/32960

[224] Fu L, Li X, Cai X, Miao D, Yao Y, and Shen Y, “Energy-guided diffusion model for CBCT-to-CT synthesis,” *Comput. Med. Imaging Graphics*, vol. 113, 2024, doi: 10.1016/j.compmedimag.2024.102344.

[225] Raju J C, Murugesan B, Ram K, and Sivaprakasam M, “AutoSyncoder: an adversarial AutoEncoder framework for multimodal MRI synthesis,” in *Lect. Notes Comput. Sci.*, Deeba F., Johnson P., Würfl T., and Ye J.C., Eds., Springer Science and Business Media Deutschland GmbH, 2020, pp. 102–110. doi: 10.1007/978-3-030-61598-7_10.

[226] Krishnan A R, Xu K, Li T Z, Remedios L W, Sandler K L, Maldonado F, and Landman B A, “Lung CT harmonization of paired reconstruction kernel images using generative adversarial networks,” *Med. Phys.*, vol. 51, no. 8, pp. 5510–5523, 2024, doi: 10.1002/mp.17028.

[227] Pradhan N, Dhaka V S, Rani G, Pradhan V, Vocaturo E, and Zumpano E, “Conditional generative adversarial network model for conversion of 2 dimensional radiographs into 3 dimensional views,” *IEEE Access*, vol. 11, pp. 96283–96296, 2023, doi: 10.1109/ACCESS.2023.3307198.

[228] Kania A, Kasymov A, Kościukiewicz J, Górak A, Mazur M, Zięba M, and Spurek P, “HyperNeRFGAN: hypernetwork approach to 3D NeRF GAN,” Aug. 22, 2024, *arXiv*: arXiv:2301.11631. doi: 10.48550/arXiv.2301.11631.

[229] Zhang X, Wu P, Zbijewski W B, Sisniega A, Han R, Jones C K, Vagdargi P, Uneri A, Helm P A, Anderson W S, *et al.*, “DL-recon: combining 3D deep learning image synthesis and model uncertainty with physics-based image reconstruction,” in *Proc SPIE Int Soc Opt Eng*, Stayman J.W., Ed., SPIE, 2022. doi: 10.1117/12.2646383.

[230] Huang J, Yang L, Wang F, Wu Y, Nan Y, Wu W, Wang C, Shi K, Aviles-Rivero A I, and Schönlieb C-B, “Enhancing global sensitivity and uncertainty quantification in medical image reconstruction with Monte Carlo arbitrary-masked mamba,” *Medical Image Analysis*, vol. 99, p. 103334, 2025, doi: 10.1016/j.media.2024.103334.

[231] Chen H, Hao Z, Guo L, and Xiao L, “Mitigating data consistency induced discrepancy in cascaded diffusion models for sparse-view ct reconstruction,” *IEEE Trans. Med. Imag.*, 2025, Accessed: Sep. 09, 2025. [Online]. Available: https://ieeexplore.ieee.org/abstract/document/10947632/

[232] Wang Y, Li Z, and Wu W, “Time-reversion fast-sampling score-based model for limited-angle CT reconstruction,” *IEEE Trans. Med. Imag.*, vol. 43, no. 10, pp. 3449–3460, 2024, doi: 10.1109/TMI.2024.3418838.

[233] Xiang L, Wang L, Gong E, Zaharchuk G, and Zhang T, “Noise-aware standard-dose PET reconstruction using general and adaptive robust loss,” in *Lect. Notes Comput. Sci.*, Liu M., Lian C., Yan P., and Cao X., Eds., Springer Science and Business Media Deutschland GmbH, 2020, pp. 654–662. doi: 10.1007/978-3-030-59861-7_66.

[234] Shi L, Zhang J, Toyonaga T, Shao D, Onofrey J A, and Lu Y, “Deep learning-based attenuation map generation with simultaneously reconstructed PET activity and attenuation and low-dose application,” *Phys. Med. Biol.*, vol. 68, no. 3, p. 35014, 2023, doi: 10.1088/1361-6560/acaf49.

[235] Wikberg E, Essen M V, Rydén T, Svensson J, Gjertsson P, and Bernhardt P, “Improvements of 177Lu SPECT images from sparsely acquired projections by reconstruction with deep-learning-generated synthetic projections,” *EJNMMI Phys.*, vol. 11, no. 1, 2024, doi: 10.1186/s40658-024-00655-x.

[236] Zhai M, Wang H, Han J, Wu T, and Ye H, “Generating PET images from low-dose data using a cycle PET reconstruction convolutional neural network,” in *IEEE Int. Conf. Mechatronics Autom., ICMA*, Institute of Electrical and Electronics Engineers Inc., 2024, pp. 369–374. doi: 10.1109/ICMA61710.2024.10632916.

[237] Leube J, Gustafsson J, Lassmann M, Salas-Ramirez M, and Tran-Gia J, “Analysis of a deep learning-based method for generation of SPECT projections based on a large monte carlo simulated dataset,” *EJNMMI Phys.*, vol. 9, no. 1, 2022, doi: 10.1186/s40658-022-00476-w.

[238] Ouyang J, Wang G, Gong E, Chen K, Pauly J, and Zaharchuk G, “Task-GAN: improving generative adversarial network for image reconstruction,” in *Lect. Notes Comput. Sci.*, Knoll F., Maier A., Rueckert D., and Ye J.C., Eds., Springer, 2019, pp. 193–204. doi: 10.1007/978-3-030-33843-5_18.

[239] Luo Y, Zhou L, Zhan B, Fei Y, Zhou J, Wang Y, and Shen D, “Adaptive rectification based adversarial network with spectrum constraint for high-quality PET image synthesis,” *Med. Image Anal.*, vol. 77, 2022, doi: 10.1016/j.media.2021.102335.

[240] Singh I R, Denker A, Barbano R, Kereta Ž, Jin B, Thielemans K, Maass P, and Arridge S, “Score-based generative models for PET image reconstruction,” *Mach. Learn. Biomed. Imaging*, vol. 2, no. Generative Models, pp. 547–585, Jan. 2024, doi: 10.59275/j.melba.2024-5d51.

[241] Xie H, Gan W, Zhou B, Chen M-K, Kulon M, Boustani A, Spencer B A, Bayerlein R, Ji W, Chen X, *et al.*, “Dose-aware diffusion model for 3D low-dose PET: multi-institutional validation with reader study and real low-dose data,” Sep. 04, 2024, *arXiv*: arXiv:2405.12996. doi: 10.48550/arXiv.2405.12996.

[242] Zhao Y, Ding Y, Lau V, Man C, Su S, Xiao L, Leong A T L, and Wu E X, “Whole-body magnetic resonance imaging at 0.05 tesla,” *Science*, vol. 384, no. 6696, p. eadm7168, May 2024, doi: 10.1126/science.adm7168.

[243] Man C, Lau V, Su S, Zhao Y, Xiao L, Ding Y, Leung G K K, Leong A T L, and Wu E X, “Deep learning enabled fast 3D brain MRI at 0.055 tesla,” *Sci. Adv.*, vol. 9, no. 38, p. eadi9327, Sep. 2023, doi: 10.1126/sciadv.adi9327.

[244] Wang G, Gong E, Banerjee S, Pauly J, and Zaharchuk G, “Accelerated MRI reconstruction with dual-domain generative adversarial network,” in *Lect. Notes Comput. Sci.*, Knoll F., Maier A., Rueckert D., and Ye J.C., Eds., Cham: Springer, 2019, pp. 47–57. doi: 10.1007/978-3-030-33843-5_5.

[245] Dar S U H, Yurt M, Shahdloo M, Ildiz M E, Tinaz B, and Cukur T, “Prior-guided image reconstruction for accelerated multi-contrast mri via generative adversarial networks,” *IEEE J. Sel. Top. Sign. Proces.*, vol. 14, no. 6, pp. 1072–1087, 2020, doi: 10.1109/JSTSP.2020.3001737.

[246] Hou R, Li F, and Zeng T, “Fast and reliable score-based generative model for parallel MRI,” *IEEE Trans. Neural Netw. Learn. Syst.*, pp. 1–14, 2023, doi: 10.1109/TNNLS.2023.3333538.

[247] Cao C, Cui Z-X, Wang Y, Liu S, Chen T, Zheng H, Liang D, and Zhu Y, “High-frequency space diffusion model for accelerated MRI,” *IEEE Trans. Med. Imag.*, vol. 43, no. 5, pp. 1853–1865, 2024, doi: 10.1109/TMI.2024.3351702.

[248] Meng Y, Yang Z, Song Z, and Shi Y, “DM-mamba: dual-domain multi-scale mamba for MRI reconstruction,” Jan. 14, 2025, *arXiv*: arXiv:2501.08163. doi: 10.48550/arXiv.2501.08163.

[249] Kofler A, Haltmeier M, Schaeffter T, Kachelrieß M, Dewey M, Wald C, and Kolbitsch C, “Neural networks-based regularization for large-scale medical image reconstruction,” *Phys. Med. Biol.*, vol. 65, no. 13, p. 135003, Jul. 2020, doi: 10.1088/1361-6560/ab990e.

[250] Kelkar V A, Bhadra S, and Anastasio M A, “Compressible latent-space invertible networks for generative model-constrained image reconstruction,” *IEEE Trans. Comput. Imaging*, vol. 7, pp. 209–223, 2021, doi: 10.1109/TCI.2021.3049648.

[251] Elmas G, Dar S U H, Korkmaz Y, Ceyani E, Susam B, Ozbey M, Avestimehr S, and Cukur T, “Federated learning of generative image priors for MRI reconstruction,” *IEEE Trans. Med. Imag.*, vol. 42, no. 7, pp. 1996–2009, 2023, doi: 10.1109/TMI.2022.3220757.

[252] Ramanarayanan S, Palla A, Ram K, and Sivaprakasam M, “Generalizing supervised deep learning MRI reconstruction to multiple and unseen contrasts using meta-learning hypernetworks[formula presented],” *Appl. Soft Comput.*, vol. 146, 2023, doi: 10.1016/j.asoc.2023.110633.

[253] Donners R, Vosshenrich J, Segeroth M, Seng M, Fenchel M, Nickel M D, Bach M, Schmaranzer F, Todorski I, Obmann M M, *et al.*, “Deep learning reconstructed new-generation 0.55 T MRI of the knee-a prospective comparison with conventional 3 T MRI,” *Invest. Radiol.*, vol. 59, no. 12, pp. 823–830, 2024, doi: 10.1097/RLI.0000000000001093.

[254] Nezhad V A, Elmas G, Kabas B, Arslan F, and Çukur T, “Generative autoregressive transformers for model-agnostic federated MRI reconstruction,” Feb. 06, 2025, *arXiv*: arXiv:2502.04521. doi: 10.48550/arXiv.2502.04521.

[255] Lan H, Li Z, He Q, and Luo J, “Fast sampling generative model for ultrasound image reconstruction,” Dec. 15, 2023, *arXiv*: arXiv:2312.09510. doi: 10.48550/arXiv.2312.09510.

[256] Zhang Y, Huneau C, Idier J, and Mateus D, “Diffusion reconstruction of ultrasound images with informative uncertainty,” Oct. 31, 2023, *arXiv*: arXiv:2310.20618. doi: 10.48550/arXiv.2310.20618.

[257] Zhang Y, Huneau C, Idier J, and Mateus D, “Ultrasound imaging based on the variance of a diffusion restoration model,” in *2024 32nd European Signal Processing Conference (EUSIPCO)*, IEEE, 2024, pp. 760–764. Accessed: Mar. 21, 2025. [Online]. Available: https://ieeexplore.ieee.org/abstract/document/10714956/

[258] Merino S, Salazar I, and Lavarello R, “Generative models for ultrasound image reconstruction from single plane-wave simulated data,” in *2024 IEEE UFFC Latin America Ultrasonics Symposium (LAUS)*, IEEE, 2024, pp. 1–4. doi: 10.1109/LAUS60931.2024.10553012.

[259] Song X, Wang G, Zhong W, Guo K, Li Z, Liu X, Dong J, and Liu Q, “Sparse-view reconstruction for photoacoustic tomography combining diffusion model with model-based iteration,” *Photoacoustics*, vol. 33, p. 100558, 2023.

[260] Tong S, Lan H, Nie L, Luo J, and Gao F, “Score-based generative models for photoacoustic image reconstruction with rotation consistency constraints,” Jun. 24, 2023, *arXiv*: arXiv:2306.13843. doi: 10.48550/arXiv.2306.13843.

[261] Zeng H, Xia N, Qian D, Hattori M, Wang C, and Kong W, “DM-RE2I: a framework based on diffusion model for the reconstruction from EEG to image,” *Biomed. Signal Process. Control*, vol. 86, p. 105125, 2023, doi: 10.1016/j.bspc.2023.105125.

[262] Xiang T, Yue W, Lin Y, Yang J, Wang Z, and Li X, “DiffCMR: fast cardiac MRI reconstruction with diffusion probabilistic models,” in *Statistical Atlases and Computational Models of the Heart. Regular and Cmrxrecon Challenge Papers*, vol. 14507, Cham: Springer Nature Switzerland, 2024, pp. 380–389. doi: 10.1007/978-3-031-52448-6_36.

[263] Yang H, Wang Z, Liu X, Li C, Xin J, and Wang Z, “Deep learning in medical image super resolution: a review,” *Appl. Intell.*, vol. 53, no. 18, pp. 20891–20916, Sep. 2023, doi: 10.1007/s10489-023-04566-9.

[264] Xiao H, Yang Z, Liu T, Liu S, Huang X, and Dai J, “Deep learning for medical imaging super-resolution: a comprehensive review,” *Neurocomputing*, p. 129667, 2025.

[265] Lucas A, Lopez-Tapia S, Molina R, and Katsaggelos A K, “Generative adversarial networks and perceptual losses for video super-resolution,” *IEEE Trans. Image Process.*, vol. 28, no. 7, pp. 3312–3327, 2019, doi: 10.1109/TIP.2019.2895768.

[266] Ren S, Li J, Guo K, and Li F, “Medical video super-resolution based on asymmetric back-projection network with multilevel error feedback,” *IEEE Access*, vol. 9, pp. 17909–17920, 2021.

[267] Song X, Tang H, Yang C, Zhou G, Wang Y, Huang X, Hua J, Coatrieux G, He X, and Chen Y, “Deformable transformer for endoscopic video super-resolution,” *Biomed. Signal Process. Control*, vol. 77, p. 103827, 2022, doi: 10.1016/j.bspc.2022.103827.

[268] Guo Y, Bi L, Ahn E, Feng D, Wang Q, and Kim J, “A spatiotemporal volumetric interpolation network for 4d dynamic medical image,” in *Proceedings of the IEEE/CVF Conference on Computer Vision and Pattern Recognition*, 2020, pp. 4726–4735. Accessed: Mar. 21, 2025. [Online]. Available: http://openaccess.thecvf.com/content_CVPR_2020/html/Guo_A_Spatiotemporal_Volumetric_Interpolation_Network_for_4D_Dynamic_Medical_Image_CVPR_2020_paper.html

[269] Wei T-T, Kuo C, Tseng Y-C, and Chen J-J, “MPVF: 4D medical image inpainting by multi-pyramid voxel flows,” *IEEE J. Biomed. Health Inform.*, vol. 27, no. 12, pp. 5872–5882, Dec. 2023, doi: 10.1109/JBHI.2023.3318127.

[270] Kim J, Yoon H, Park G, Kim K, and Yang E, “Data-efficient unsupervised interpolation without any intermediate frame for 4D medical images,” in *2024 IEEE/CVF Conference on Computer Vision and Pattern Recognition (CVPR)*, Seattle, WA, USA: IEEE, Jun. 2024, pp. 11353–11364. doi: 10.1109/CVPR52733.2024.01079.

[271] Chatterjee S, Sarasaen C, Rose G, Nürnberger A, and Speck O, “Ddos-unet: incorporating temporal information using dynamic dual-channel unet for enhancing super-resolution of dynamic mri,” *IEEE Access*, 2024, doi: 10.1109/ACCESS.2024.3427674.

[272] Karani N, Zhang L, Tanner C, and Konukoglu E, “An image interpolation approach for acquisition time reduction in navigator-based 4D MRI,” *Med. Image Anal.*, vol. 54, pp. 20–29, 2019, doi: 10.1016/j.media.2019.02.008.

[273] Zhang K, Hu H, Philbrick K, Conte G M, Sobek J D, Rouzrokh P, and Erickson B J, “SOUP-GAN: super-resolution MRI using generative adversarial networks,” *Tomography*, vol. 8, no. 2, pp. 905–919, 2022, doi: 10.3390/tomography8020073.

[274] Balasubramanian A, Dhanasekaran H, Raghu B, and Kumarasamy K, “MRI super-resolution using generative adversarial network and discrete wavelet transform,” in *Proc. - Int. Conf. Augment. Intell. Sustain. Syst., ICAISS*, Institute of Electrical and Electronics Engineers Inc., 2022, pp. 1314–1318. doi: 10.1109/ICAISS55157.2022.10010995.

[275] Ma Q, Koh J C, and Lee W S, “A frequency domain constraint for synthetic and real X-ray image super resolution,” in *Lect. Notes Comput. Sci.*, Haq N., Johnson P., Maier A., Würfl T., and Yoo J., Eds., Springer Science and Business Media Deutschland GmbH, 2021, pp. 120–129. doi: 10.1007/978-3-030-88552-6_12.

[276] Sun L, Chen J, Xu Y, Gong M, Yu K, and Batmanghelich K, “Hierarchical amortized GAN for 3D high resolution medical image synthesis,” *IEEE J. Biomed. Health Inform.*, vol. 26, no. 8, pp. 3966–3975, Aug. 2022, doi: 10.1109/JBHI.2022.3172976.

[277] Huang W, Liao X, Chen H, Hu Y, Jia W, and Wang Q, “Deep local-to-global feature learning for medical image super-resolution,” *Comput. med. imaging graph. : off. j. Comput. Med. Imaging Soc.*, vol. 115, p. 102374, Jul. 2024, doi: 10.1016/j.compmedimag.2024.102374.

[278] Zhao K, Pang K, Hung A L Y, Zheng H, Yan R, and Sung K, “Mri super-resolution with partial diffusion models,” *IEEE Trans. Med. Imag.*, 2024, Accessed: Apr. 06, 2025. [Online]. Available: https://ieeexplore.ieee.org/abstract/document/10720924/

[279] Ji Z, Zou B, Kui X, Vera P, and Ruan S, “Deform-mamba network for MRI super-resolution,” in *Medical Image Computing and Computer Assisted Intervention – MICCAI 2024*, vol. 15007, Cham: Springer Nature Switzerland, 2024, pp. 242–252. doi: 10.1007/978-3-031-72104-5_24.

[280] Chong C K and Ho E T W, “Synthesis of 3D MRI brain images with shape and texture generative adversarial deep neural networks,” *IEEE Access*, vol. 9, pp. 64747–64760, 2021, doi: 10.1109/ACCESS.2021.3075608.

[281] Hong S, Marinescu R, Dalca A V, Bonkhoff A K, Bretzner M, Rost N S, and Golland P, “3D-StyleGAN: a style-based generative adversarial network for generative modeling of three-dimensional medical images,” in *Deep Generative Models, and Data Augmentation, Labelling, and Imperfections: First Workshop, DGM4MICCAI 2021, and First Workshop, DALI 2021, Held in Conjunction with MICCAI 2021, Strasbourg, France, October 1, 2021, Proceedings 1*, Springer, 2021, pp. 24–34. Accessed: Apr. 01, 2025. [Online]. Available: https://link.springer.com/chapter/10.1007/978-3-030-88210-5_3

[282] Txurio M S, Román K L-L, Marcos-Carrión A, Castellote-Huguet P, Santabárbara-Gómez J M, Oliver I M, and Ballester M A G, “Diffusion models for realistic CT image generation,” in *Smart Innov. Syst. Technol.*, Chen Y.-W., Tanaka S., Howlett R.J., and Jain L.C., Eds., Springer Science and Business Media Deutschland GmbH, 2023, pp. 335–344. doi: 10.1007/978-981-99-3311-2_30.

[283] Wu H, Zhao Z, Zhang Y, Xie W, and Wang Y, “MRGen: diffusion-based controllable data engine for MRI segmentation towards unannotated modalities,” Dec. 04, 2024, *arXiv*: arXiv:2412.04106. doi: 10.48550/arXiv.2412.04106.

[284] Zhang S, Liu J, Hu B, and Mao Z, “GH-DDM: the generalized hybrid denoising diffusion model for medical image generation,” *Multimedia Syst.*, vol. 29, no. 3, pp. 1335–1345, 2023, doi: 10.1007/s00530-023-01059-0.

[285] Zheng J-Q, Mo Y, Sun Y, Li J, Wu F, Wang Z, Vincent T, and Papież B W, “Deformation-recovery diffusion model (DRDM): instance deformation for image manipulation and synthesis,” Jul. 21, 2024, *arXiv*: arXiv:2407.07295. doi: 10.48550/arXiv.2407.07295.

[286] Danu M, Nita C-I, Vizitiu A, Suciu C, and Itu L M, “Deep learning based generation of synthetic blood vessel surfaces,” in *Int. Conf. Syst. Theory, Control Comput., ICSTCC - Proc.*, Precup R.-E., Ed., Institute of Electrical and Electronics Engineers Inc., 2019, pp. 662–667. doi: 10.1109/ICSTCC.2019.8885576.

[287] Xu T, Zhang P, Huang Q, Zhang H, Gan Z, Huang X, and He X, “Attngan: fine-grained text to image generation with attentional generative adversarial networks,” in *Proceedings of the IEEE Conference on Computer Vision and Pattern Recognition*, arXiv, 2018, pp. 1316–1324. Accessed: Sep. 09, 2025. [Online]. Available: http://openaccess.thecvf.com/content_cvpr_2018/html/Xu_AttnGAN_Fine-Grained_Text_CVPR_2018_paper.html

[288] Qiao T, Zhang J, Xu D, and Tao D, “Mirrorgan: learning text-to-image generation by redescription,” in *Proceedings of the IEEE/CVF Conference on Computer Vision and Pattern Recognition*, arXiv, 2019, pp. 1505–1514. Accessed: Mar. 17, 2025. [Online]. Available: http://openaccess.thecvf.com/content_CVPR_2019/html/Qiao_MirrorGAN_Learning_Text-To-Image_Generation_by_Redescription_CVPR_2019_paper.html

[289] Sahithi Y L, Sunny N, Deepak M M L, and Amrutha S, “Text-to-image synthesis using stackGAN,” in *Glob. Conf. Inf. Technol. Commun., GCITC*, Institute of Electrical and Electronics Engineers Inc., 2023. doi: 10.1109/GCITC60406.2023.10426184.

[290] Ding M, Yang Z, Hong W, Zheng W, Zhou C, Yin D, Lin J, Zou X, Shao Z, Yang H, *et al.*, “CogView: Mastering text-to-image generation via transformers,” *Adv. Neural Inf. Process. Syst.*, vol. 34, pp. 19822–19835, 2021.

[291] Reddy M D M, Basha M S M, Hari M M C, and Penchalaiah M N, “Dall-e: Creating images from text,” *Ugc Care Group J.*, vol. 8, no. 14, pp. 71–75, 2021.

[292] Saharia C, Chan W, Saxena S, Li L, Whang J, Denton E L, Ghasemipour K, Gontijo Lopes R, Karagol Ayan B, and Salimans T, “Photorealistic text-to-image diffusion models with deep language understanding,” *Adv. Neural Inf. Process. Syst.*, vol. 35, pp. 36479–36494, 2022.

[293] Rombach R, Blattmann A, Lorenz D, Esser P, and Ommer B, “High-resolution image synthesis with latent diffusion models,” in *Proceedings of the Ieee/cvf Conference on Computer Vision and Pattern Recognition*, 2022, pp. 10684–10695. Accessed: Mar. 13, 2025. [Online]. Available: https://openaccess.thecvf.com/content/CVPR2022/html/Rombach_High-Resolution_Image_Synthesis_With_Latent_Diffusion_Models_CVPR_2022_paper

[294] Li X, Shuai Y, Liu C, Chen Q, Wu Q, Guo P, Yang D, Zhao C, Bassi P R A S, Xu D, *et al.*, “Text-driven tumor synthesis,” Dec. 24, 2024, *arXiv*: arXiv:2412.18589. doi: 10.48550/arXiv.2412.18589.

[295] Guo P, Zhao C, Yang D, Xu Z, Nath V, Tang Y, Simon B, Belue M, Harmon S, and Turkbey B, “Maisi: medical ai for synthetic imaging,” in *2025 IEEE/CVF Winter Conference on Applications of Computer Vision (WACV)*, IEEE, 2025, pp. 4430–4441. doi: 10.1109/WACV61041.2025.00435.

[296] Xu Y, Sun L, Peng W, Jia S, Morrison K, Perer A, Zandifar A, Visweswaran S, Eslami M, and Batmanghelich K, “MedSyn: text-guided anatomy-aware synthesis of high-fidelity 3-D CT images,” *IEEE Trans. Med. Imag.*, vol. 43, no. 10, pp. 3648–3660, 2024, doi: 10.1109/TMI.2024.3415032.

[297] Qiao M, Wang S, Qiu H, De Marvao A, O’Regan D P, Rueckert D, and Bai W, “Cheart: a conditional spatio-temporal generative model for cardiac anatomy,” *IEEE Trans. Med. Imag.*, vol. 43, no. 3, pp. 1259–1269, 2023, doi: 10.1109/TMI.2023.3331982.

[298] Ben-Cohen A, Klang E, Raskin S P, Soffer S, Ben-Haim S, Konen E, Amitai M M, and Greenspan H, “Cross-modality synthesis from CT to PET using FCN and GAN networks for improved automated lesion detection,” *Eng. Appl. Artif. Intell.*, vol. 78, pp. 186–194, 2019.

[299] Haubold J, Hosch R, Umutlu L, Wetter A, Haubold P, Radbruch A, Forsting M, Nensa F, and Koitka S, “Contrast agent dose reduction in computed tomography with deep learning using a conditional generative adversarial network,” *Eur. Radio.*, vol. 31, no. 8, pp. 6087–6095, 2021, doi: 10.1007/s00330-021-07714-2.

[300] Jiao J, Namburete A I, Papageorghiou A T, and Noble J A, “Self-supervised ultrasound to MRI fetal brain image synthesis,” *IEEE Trans. Med. Imag.*, vol. 39, no. 12, pp. 4413–4424, 2020, doi: 10.1109/TMI.2020.3018560.

[301] Wang Z, Zhang L, Wang L, and Zhang Z, “Soft masked mamba diffusion model for CT to MRI conversion,” Jun. 22, 2024, *arXiv*: arXiv:2406.15910. doi: 10.48550/arXiv.2406.15910.

[302] Liu C, Zhang Y, Tao Y, Lyu T, Xi Y, and Chen Y, “Frequency space mamba enhanced bidirectional generative network for dual-source CBCT,” in *Proc SPIE Int Soc Opt Eng*, Luo Q., Li X., Gu Y., and Zhu D., Eds., SPIE, 2024, pp. 61–68. doi: 10.1117/12.3033807.

[303] Atli O F, Kabas B, Arslan F, Demirtas A C, Yurt M, Dalmaz O, and Çukur T, “I2I-mamba: multi-modal medical image synthesis via selective state space modeling,” Nov. 15, 2024, *arXiv*: arXiv:2405.14022. doi: 10.48550/arXiv.2405.14022.

[304] Zhou X, Wu J, Zhao H, Chen L, Zhang S, Wang G, and Wang G, “GLFC: unified global-local feature and contrast learning with mamba-enhanced UNet for synthetic CT generation from CBCT,” Jan. 06, 2025, *arXiv*: arXiv:2501.02992. doi: 10.48550/arXiv.2501.02992.

[305] Liu J, Pasumarthi S, Duffy B, Gong E, Datta K, and Zaharchuk G, “One model to synthesize them all: multi-contrast multi-scale transformer for missing data imputation,” in *IEEE Trans. Med. Imaging*, Institute of Electrical and Electronics Engineers Inc., 2023, pp. 2577–2591. doi: 10.1109/TMI.2023.3261707.

[306] Kaplan S, Perrone A, Alexopoulos D, Kenley J K, Barch D M, Buss C, Elison J T, Graham A M, Neil J J, and O’Connor T G, “Synthesizing pseudo-T2w images to recapture missing data in neonatal neuroimaging with applications in rs-fMRI,” *Neuroimage*, vol. 253, p. 119091, 2022, doi: 10.1016/j.neuroimage.2022.119091.

[307] Yan K, Liu Z, Zheng S, Guo Z, Zhu Z, and Zhao Y, “Coarse-to-fine learning framework for semi-supervised multimodal MRI synthesis,” in *Lect. Notes Comput. Sci.*, Wallraven C., Liu Q., and Nagahara H., Eds., Springer Science and Business Media Deutschland GmbH, 2022, pp. 370–384. doi: 10.1007/978-3-031-02444-3_28.

[308] Xiao X, Hu Q V, and Wang G, “FgC2F-UDiff: frequency-guided and coarse-to-fine unified diffusion model for multi-modality missing MRI synthesis,” *IEEE Trans. Comput. Imaging*, 2024, doi: 10.1109/TCI.2024.3516574.

[309] Zhang Y, Peng C, Wang Q, Song D, Li K, and Zhou S K, “Unified multi-modal image synthesis for missing modality imputation,” *IEEE Trans. Med. Imag.*, 2024, Accessed: Jan. 04, 2025. [Online]. Available: https://ieeexplore.ieee.org/abstract/document/10589432/

[310] Zhou B, Zhou Q, Miao C, Liu Y, and Guo Y, “Cross-dimensional knowledge-guided synthesizer trained with unpaired multimodality MRIs,” *Soft Comput.*, vol. 28, no. 13–14, pp. 8393–8408, Jul. 2024, doi: 10.1007/s00500-024-09700-4.

[311] Guo P, Wang P, Yasarla R, Zhou J, Patel V M, and Jiang S, “Anatomic and molecular MR image synthesis using confidence guided CNNs,” *IEEE Trans. Med. Imag.*, vol. 40, no. 10, pp. 2832–2844, 2020, doi: 10.1109/TMI.2020.3046460.

[312] Shen Z, Ouyang X, Xiao B, Cheng J-Z, Shen D, and Wang Q, “Image synthesis with disentangled attributes for chest x-ray nodule augmentation and detection,” *Med. Image Anal.*, vol. 84, p. 102708, 2023, doi: 10.1016/j.media.2022.102708.

[313] Hou B, “High-fidelity diabetic retina fundus image synthesis from freestyle lesion maps,” *Biomed. Opt. Express*, vol. 14, no. 2, p. 533, Feb. 2023, doi: 10.1364/BOE.477906.

[314] Guo W, Zhang H, Wan S, Zou B, Wang W, and Jin P, “LN-gen: rectal lymph nodes generation via anatomical features,” Aug. 27, 2024, *arXiv*: arXiv:2408.14977. doi: 10.48550/arXiv.2408.14977.

[315] Castillo M H G del, Garcia R M, Mazón M J C, Garcia E A, and Fernández-Miranda P M, “Diffusion models for conditional MRI generation,” Feb. 25, 2025, *arXiv*: arXiv:2502.18620. doi: 10.48550/arXiv.2502.18620.

[316] Raad R, Ray D, Varghese B, Hwang D, Gill I, Duddalwar V, and Oberai A A, “Conditional generative learning for medical image imputation,” *Sci. Rep.*, vol. 14, no. 1, p. 171, Jan. 2024, doi: 10.1038/s41598-023-50566-7.

[317] Lai X, Geier O M, Fleischer T, Garred Ø, Borgen E, Funke S W, Kumar S, Rognes M E, Seierstad T, and Børresen-Dale A-L, “Toward personalized computer simulation of breast cancer treatment: a multiscale pharmacokinetic and pharmacodynamic model informed by multitype patient data,” *Cancer Res.*, vol. 79, no. 16, pp. 4293–4304, 2019, doi: 10.1158/0008-5472.CAN-18-1804.

[318] Savić M, Kurbalija V, Balaz I, and Ivanović M, “Heterogeneous tumour modeling using PhysiCell and its implications in precision medicine,” in *Cancer, Complexity, Computation*, vol. 46, Cham: Springer International Publishing, 2022, pp. 157–189. doi: 10.1007/978-3-031-04379-6_7.

[319] Liu S, Zhang J, Li T, Yan H, and Liu J, “Technical note: a cascade 3D U‐net for dose prediction in radiotherapy,” *Med. Phys.*, vol. 48, no. 9, pp. 5574–5582, Sep. 2021, doi: 10.1002/mp.15034.

[320] Radonic D, Xiao F, Wahl N, Voss L, Neishabouri A, Delopoulos N, Marschner S, Corradini S, Belka C, Dedes G, *et al.*, “Proton dose calculation with LSTM networks in presence of a magnetic field,” *Phys. Med. Biol.*, vol. 69, no. 21, p. 215019, Nov. 2024, doi: 10.1088/1361-6560/ad7f1e.

[321] Fu L, Li X, Cai X, Wang Y, Wang X, Yao Y, and Shen Y, “SP-DiffDose: a conditional diffusion model for radiation dose prediction based on multi-scale fusion of anatomical structures, guided by SwinTransformer and projector,” Dec. 11, 2023, *arXiv*: arXiv:2312.06187. doi: 10.48550/arXiv.2312.06187.

[322] Pan S, Chang C-W, Axente M, Wang T, Shelton J, Liu T, Roper J, and Yang X, “Data-driven volumetric image generation from surface structures using a patient-specific deep leaning model,” *Arxiv*, p. arXiv-2304, 2023.

[323] Zhou M and Khalvati F, “Conditional generation of 3d brain tumor regions via VQGAN and temporal-agnostic masked transformer,” in *Medical Imaging with Deep Learning*, 2024. Accessed: Mar. 21, 2025. [Online]. Available: https://raw.githubusercontent.com/mlresearch/v250/main/assets/zhou24a/zhou24a.pdf

[324] Pellicer A O, Yadav A K S, Bhagtani K, Xiang Z, Pizlo Z, Gradus-Pizlo I, and Delp E J, “Generation of synthetic echocardiograms using video diffusion models,” in *Proc IEEE Southwest Symp Image Anal Interpret*, Institute of Electrical and Electronics Engineers Inc., 2024, pp. 33–36. doi: 10.1109/SSIAI59505.2024.10508643.

[325] Li C, Liu H, Liu Y, Feng B Y, Li W, Liu X, Chen Z, Shao J, and Yuan Y, “Endora: video generation models as endoscopy simulators,” in *Medical Image Computing and Computer Assisted Intervention – MICCAI 2024*, vol. 15006, Cham: Springer Nature Switzerland, 2024, pp. 230–240. doi: 10.1007/978-3-031-72089-5_22.

[326] Hu D, Zhang C, Fei X, Yao Y, Xi Y, Liu J, Zhang Y, Coatrieux G, Coatrieux J L, and Chen Y, “DPI-MoCo: deep prior image constrained motion compensation reconstruction for 4D CBCT,” *IEEE Trans. Med. Imag.*, 2024, doi: 10.1109/TMI.2024.3483451.

[327] Han L, Tan T, Zhang T, Huang Y, Wang X, Gao Y, Teuwen J, and Mann R, “Synthesis-based imaging-differentiation representation learning for multi-sequence 3D/4D MRI,” *Med. Image Anal.*, vol. 92, 2024, doi: 10.1016/j.media.2023.103044.

[328] Chen Q, Chen X, Song H, Xiong Z, Yuille A, Wei C, and Zhou Z, “Towards generalizable tumor synthesis,” in *Proceedings of the IEEE/CVF Conference on Computer Vision and Pattern Recognition*, 2024, pp. 11147–11158. Accessed: Mar. 21, 2025. [Online]. Available: http://openaccess.thecvf.com/content/CVPR2024/html/Chen_Towards_Generalizable_Tumor_Synthesis_CVPR_2024_paper.html

[329] Takahashi W, Oshikawa S, and Mori S, “Real-time markerless tumour tracking with patient-specific deep learning using a personalised data generation strategy: proof of concept by phantom study,” *Br. J. Radiol.*, vol. 93, no. 1109, 2020, doi: 10.1259/bjr.20190420.

[330] Ravi D, Alexander D C, and Oxtoby N P, “Degenerative adversarial NeuroImage nets: generating images that mimic disease progression,” in *Lect. Notes Comput. Sci.*, Shen D., Yap P.-T., Liu T., Peters T.M., Khan A., Staib L.H., Essert C., and Zhou S., Eds., Springer Science and Business Media Deutschland GmbH, 2019, pp. 164–172. doi: 10.1007/978-3-030-32248-9_19.

[331] Song L, Wang Q, Li H, Fan J, and Hu B, “Longitudinal structural MRI data prediction in nondemented and demented older adults via generative adversarial convolutional network,” *Neural Process. Lett.*, vol. 55, no. 2, pp. 989–999, Apr. 2023, doi: 10.1007/s11063-022-10922-6.

[332] Shi K, Li Y, Ho B, Wang J, and Guo K, “Universal lesion segmentation challenge 2023: a comparative research of different algorithms,” Feb. 14, 2025, *arXiv*: arXiv:2502.10608. doi: 10.48550/arXiv.2502.10608.

[333] D’Antonoli T A, Berger L K, Indrakanti A K, Vishwanathan N, Weiß J, Jung M, Berkarda Z, Rau A, Reisert M, Küstner T, *et al.*, “TotalSegmentator MRI: robust sequence-independent segmentation of multiple anatomic structures in MRI,” *Radiology*, vol. 314, no. 2, p. e241613, Feb. 2025, doi: 10.1148/radiol.241613.

[334] Li X, Luo G, Wang K, Wang H, Liu J, Liang X, Jiang J, Song Z, Zheng C, Chi H, *et al.*, “The state-of-the-art 3D anisotropic intracranial hemorrhage segmentation on non-contrast head CT: the INSTANCE challenge,” Jan. 12, 2023, *arXiv*: arXiv:2301.03281. doi: 10.48550/arXiv.2301.03281.

[335] Luo X, Fu J, Zhong Y, Liu S, Han B, Astaraki M, Bendazzoli S, Toma-Dasu I, Ye Y, and Chen Z, “Segrap2023: a benchmark of organs-at-risk and gross tumor volume segmentation for radiotherapy planning of nasopharyngeal carcinoma,” *Med. Image Anal.*, vol. 101, p. 103447, 2025, doi: 10.1016/j.media.2024.103447.

[336] LaBella D, Adewole M, Alonso-Basanta M, Altes T, Anwar S M, Baid U, Bergquist T, Bhalerao R, Chen S, Chung V, *et al.*, “The ASNR-MICCAI brain tumor segmentation (BraTS) challenge 2023: intracranial meningioma,” May 12, 2023, *arXiv*: arXiv:2305.07642. doi: 10.48550/arXiv.2305.07642.

[337] Zbontar J, Knoll F, Sriram A, Murrell T, Huang Z, Muckley M J, Defazio A, Stern R, Johnson P, Bruno M, *et al.*, “fastMRI: An Open Dataset and Benchmarks for Accelerated MRI,” Dec. 11, 2019, *arXiv*: arXiv:1811.08839. doi: 10.48550/arXiv.1811.08839.

[338] Jiang D, Dou W, Vosters L, Xu X, Sun Y, and Tan T, “Denoising of 3D magnetic resonance images with multi-channel residual learning of convolutional neural network,” *Jpn. J. Radiol.*, vol. 36, no. 9, pp. 566–574, Sep. 2018, doi: 10.1007/s11604-018-0758-8.

[339] Snoek L, van der Miesen M M, Beemsterboer T, Van Der Leij A, Eigenhuis A, and Steven Scholte H, “The Amsterdam open MRI collection, a set of multimodal MRI datasets for individual difference analyses,” *Sci. Data*, vol. 8, no. 1, p. 85, 2021, doi: 10.1038/s41597-021-00870-6.

[340] Campello V M, Gkontra P, Izquierdo C, Martin-Isla C, Sojoudi A, Full P M, Maier-Hein K, Zhang Y, He Z, and Ma J, “Multi-centre, multi-vendor and multi-disease cardiac segmentation: the M&ms challenge,” *IEEE Trans. Med. Imag.*, vol. 40, no. 12, pp. 3543–3554, 2021, doi: 10.1109/TMI.2021.3090082.

[341] Galazis C, Wu H, Li Z, Petri C, Bharath A A, and Varela M, “Tempera: spatial transformer feature pyramid network for cardiac MRI segmentation,” in *Statistical Atlases and Computational Models of the Heart. Multi-disease, Multi-view, and Multi-center Right Ventricular Segmentation in Cardiac MRI Challenge*, vol. 13131, Cham: Springer International Publishing, 2022, pp. 268–276. doi: 10.1007/978-3-030-93722-5_29.

[342] Chen C, Liu Y, Schniter P, Tong M, Zareba K, Simonetti O, Potter L, and Ahmad R, “OCMR (v1.0)--open-access multi-coil k-space dataset for cardiovascular magnetic resonance imaging,” Aug. 12, 2020, *arXiv*: arXiv:2008.03410. doi: 10.48550/arXiv.2008.03410.

[343] Ma J, Zhang Y, Gu S, Ge C, Mae S, Young A, Zhu C, Yang X, Meng K, and Huang Z, “Unleashing the strengths of unlabelled data in deep learning-assisted pan-cancer abdominal organ quantification: the FLARE22 challenge,” *Lancet Digit. Health*, vol. 6, no. 11, pp. e815–e826, 2024, doi: 10.1016/S2589-7500(24)00154-7.

[344] Ma J, Zhang Y, Gu S, Ge C, Wang E, Zhou Q, Huang Z, Lyu P, He J, and Wang B, “Automatic organ and pan-cancer segmentation in abdomen CT: the FLARE 2023 challenge,” Aug. 22, 2024, *arXiv*: arXiv:2408.12534. doi: 10.48550/arXiv.2408.12534.

[345] Ma J, Zhang Y, Gu S, Zhu C, Ge C, Zhang Y, An X, Wang C, Wang Q, and Liu X, “Abdomenct-1k: is abdominal organ segmentation a solved problem?,” *IEEE Trans. Pattern Anal. Mach. Intell.*, vol. 44, no. 10, pp. 6695–6714, 2021, doi: 10.1109/TPAMI.2021.3100536.

[346] Li X, Vesal S, Saunders S, John S, Soerensen C, Jahanandish H, Moroianu S, Bhattacharya I, Fan R E, and Sonn G A, “The prostate imaging: cancer AI (PI-CAI) 2022 grand challenge (PIMed team),” *Dep. Radiol. Stanf. Univ. Stanf. CA 94305 USA; Dep. Urol. Stanf. Univ. Stanf. CA 94305 USA; Inst. Comput. Math. Eng. Stanf. CA 94305 USA*, Accessed: Mar. 25, 2025. [Online]. Available: http://rumc-gcorg-p-public.s3.amazonaws.com/evaluation-supplementary/642/6dedd552-7e95-49d2-905f-23979d111e63/PI_CAI_2022_Workshop_Paper.pdf

[347] Ying N, Lei Y, Zhang T, Lyu S, Li C, Chen S, Liu Z, Zhao Y, and Zhang G, “CPIA dataset: a comprehensive pathological image analysis dataset for self-supervised learning pre-training,” Oct. 27, 2023, *arXiv*: arXiv:2310.17902. doi: 10.48550/arXiv.2310.17902.

[348] Spanhol F A, Oliveira L S, Petitjean C, and Heutte L, “A dataset for breast cancer histopathological image classification,” *IEEE Trans. Bio-Med. Eng.*, vol. 63, no. 7, pp. 1455–1462, 2015, doi: 10.1109/TBME.2015.2496264.

[349] Zingman I, Stierstorfer B, Lempp C, and Heinemann F, “Learning image representations for anomaly detection: application to discovery of histological alterations in drug development,” *Med. Image Anal.*, vol. 92, p. 103067, 2024, doi: 10.1016/j.media.2023.103067.

[350] Li F, Hu Z, Chen W, and Kak A, “Adaptive supervised PatchNCE loss for learning H&E-to-IHC stain translation with inconsistent groundtruth image pairs,” in *Medical Image Computing and Computer Assisted Intervention – MICCAI 2023*, vol. 14225, Cham: Springer Nature Switzerland, 2023, pp. 632–641. doi: 10.1007/978-3-031-43987-2_61.

[351] Song A H, Jaume G, Williamson D F, Lu M Y, Vaidya A, Miller T R, and Mahmood F, “Artificial intelligence for digital and computational pathology,” *Nat. Rev. Bioeng.*, vol. 1, no. 12, pp. 930–949, 2023, doi: 10.1038/s44222-023-00096-8.

[352] Gong H, Chen G, Wang R, Xie X, Mao M, Yu Y, Chen F, and Li G, “Multi-task learning for thyroid nodule segmentation with thyroid region prior,” in *2021 IEEE 18th International Symposium on Biomedical Imaging (ISBI)*, IEEE, 2021, pp. 257–261. doi: 10.1109/ISBI48211.2021.9434087.

[353] Duffy G, Cheng P P, Yuan N, He B, Kwan A C, Shun-Shin M J, Alexander K M, Ebinger J, Lungren M P, and Rader F, “High-throughput precision phenotyping of left ventricular hypertrophy with cardiovascular deep learning,” *JAMA Cardiol.*, vol. 7, no. 4, pp. 386–395, 2022, doi: 10.1001/jamacardio.2021.6059.

[354] Ozyoruk K B, Gokceler G I, Bobrow T L, Coskun G, Incetan K, Almalioglu Y, Mahmood F, Curto E, Perdigoto L, and Oliveira M, “EndoSLAM dataset and an unsupervised monocular visual odometry and depth estimation approach for endoscopic videos,” *Med. Image Anal.*, vol. 71, p. 102058, 2021, doi: 10.1016/j.media.2021.102058.

[355] Subramanian M, Shanmugavadivel K, Naren O S, Premkumar K, and Rankish K, “Classification of retinal oct images using deep learning,” in *2022 International Conference on Computer Communication and Informatics (ICCCI)*, IEEE, 2022, pp. 1–7. doi: 10.1109/ICCCI54379.2022.9740985.

[356] Herrera-Chavez A I, Rodríguez-Martínez E A, Flores-Fuentes W, Rodgíruez-Quiñonez J C, García-Gallegos J C, Montiel-Ross O H, Gonzàalez-Navarro F F, and Sergiyenko O, “Multi-label image classification for ocular disease diagnosis using K-fold cross-validation on the ODIR-5K dataset,” in *2024 IEEE 33rd International Symposium on Industrial Electronics (ISIE)*, IEEE, 2024, pp. 1–6. doi: 10.1109/ISIE54533.2024.10595740.

[357] Li L, Xu M, Wang X, Jiang L, and Liu H, “Attention based glaucoma detection: a large-scale database and CNN model,” in *Proceedings of the IEEE/CVF Conference on Computer Vision and Pattern Recognition*, 2019, pp. 10571–10580. Accessed: Mar. 25, 2025. [Online]. Available: http://openaccess.thecvf.com/content_CVPR_2019/html/Li_Attention_Based_Glaucoma_Detection_A_Large-Scale_Database_and_CNN_Model_CVPR_2019_paper.html

[358] De Vente C, Vermeer K A, Jaccard N, Wang H, Sun H, Khader F, Truhn D, Aimyshev T, Zhanibekuly Y, and Le T-D, “Airogs: artificial intelligence for robust glaucoma screening challenge,” *IEEE Trans. Med. Imag.*, vol. 43, no. 1, pp. 542–557, 2023.

[359] Hu X, Gu L, Kobayashi K, Liu L, Zhang M, Harada T, Summers R M, and Zhu Y, “Interpretable medical image visual question answering via multi-modal relationship graph learning,” *Med. Image Anal.*, vol. 97, p. 103279, 2024.

[360] Bustos A, Pertusa A, Salinas J-M, and De La Iglesia-Vaya M, “Padchest: a large chest x-ray image dataset with multi-label annotated reports,” *Med. Image Anal.*, vol. 66, p. 101797, 2020.

[361] Huang Z, Bianchi F, Yuksekgonul M, Montine T J, and Zou J, “A visual–language foundation model for pathology image analysis using medical twitter,” *Nat. Med.*, vol. 29, no. 9, pp. 2307–2316, 2023, doi: 10.1038/s41591-023-02504-3.

[362] Lin W, Zhao Z, Zhang X, Wu C, Zhang Y, Wang Y, and Xie W, “PMC-CLIP: contrastive language-image pre-training using biomedical documents,” in *Medical Image Computing and Computer Assisted Intervention – MICCAI 2023*, vol. 14227, Cham: Springer Nature Switzerland, 2023, pp. 525–536. doi: 10.1007/978-3-031-43993-3_51.

[363] Saha A, Harowicz M R, Grimm L J, Kim C E, Ghate S V, Walsh R, and Mazurowski M A, “A machine learning approach to radiogenomics of breast cancer: a study of 922 subjects and 529 DCE-MRI features,” *Br. J. Cancer*, vol. 119, no. 4, pp. 508–516, 2018, doi: 10.1038/s41416-018-0185-8.

[364] Li W, Newitt D C, and Gibbs J, “I-SPY 2 breast dynamic contrast enhanced MRI trial (ISPY2),” *Cancer Imaging Arch.*, 2023.

[365] Yang J, Shi R, Wei D, Liu Z, Zhao L, Ke B, Pfister H, and Ni B, “MedMNIST v2 - a large-scale lightweight benchmark for 2D and 3D biomedical image classification,” *Sci. Data*, vol. 10, no. 1, p. 41, Jan. 2023, doi: 10.1038/s41597-022-01721-8.

[366] Zhu J, “Method for MICCAI FLARE24 challenge,” in *MICCAI 2024 FLARE Challenge*, Accessed: Mar. 24, 2025. [Online]. Available: https://openreview.net/forum?id=lvH839PT2D

[367] Rister B, Yi D, Shivakumar K, Nobashi T, and Rubin D L, “CT-ORG, a new dataset for multiple organ segmentation in computed tomography,” *Sci. Data*, vol. 7, no. 1, p. 381, 2020, doi: 10.1038/s41597-020-00715-8.

[368] Shapey J, Kujawa A, Dorent R, Wang G, Dimitriadis A, Grishchuk D, Paddick I, Kitchen N, Bradford R, and Saeed S R, “Segmentation of vestibular schwannoma from MRI, an open annotated dataset and baseline algorithm,” *Sci. Data*, vol. 8, no. 1, p. 286, 2021, doi: 10.1038/s41597-021-01064-w.

[369] Gireesha H M and Nanda S, “Thyroid nodule segmentation and classification in ultrasound images,” *Int. J. Eng. Res. Technol.*, 2014, Accessed: Mar. 25, 2025. [Online]. Available: https://www.researchgate.net/profile/Gireesha-H-M-2/publication/332013003_Thyroid_Nodule_Segmentation_and_Classification_in_Ultrasound_Images/links/5c9b1f3c45851506d72db73f/Thyroid-Nodule-Segmentation-and-Classification-in-Ultrasound-Images.pdf

[370] Anna Montoya, Hasnin, kaggle446, shirzad, Will Cukierski, and yffud, “Ultrasound nerve segmentation.” Accessed: Mar. 25, 2025. [Online]. Available: https://kaggle.com/ultrasound-nerve-segmentation

[371] Pires R, Avila S, Wainer J, Valle E, Abramoff M D, and Rocha A, “A data-driven approach to referable diabetic retinopathy detection,” *Artif. Intell. Med.*, vol. 96, pp. 93–106, 2019, doi: 10.1016/j.artmed.2019.03.009.

[372] Rahman T Y, Mahanta L B, Chakraborty C, Das A K, and Sarma J D, “Textural pattern classification for oral squamous cell carcinoma,” *J. Microsc.*, vol. 269, no. 1, pp. 85–93, Jan. 2018, doi: 10.1111/jmi.12611.

[373] Elias P and Bhave S, “CheXchoNet: a chest radiograph dataset with gold standard echocardiography labels”, Accessed: Mar. 25, 2025. [Online]. Available: https://physionet.org/content/chexchonet/

[374] Reis E P, De Paiva J P, Da Silva M C, Ribeiro G A, Paiva V F, Bulgarelli L, Lee H M, Santos P V, Brito V M, and Amaral L T, “BRAX, brazilian labeled chest x-ray dataset,” *Sci. Data*, vol. 9, no. 1, p. 487, 2022.

[375] Lakhani P, Mongan J, Singhal C, Zhou Q, Andriole K P, Auffermann W F, Prasanna P M, Pham T X, Peterson M, Bergquist P J, *et al.*, “The 2021 SIIM-FISABIO-RSNA machine learning COVID-19 challenge: annotation and standard exam classification of COVID-19 chest radiographs,” *J. Digital Imaging*, vol. 36, no. 1, pp. 365–372, Sep. 2022, doi: 10.1007/s10278-022-00706-8.

[376] Pehrson L M, Nielsen M B, and Ammitzbøl Lauridsen C, “Automatic pulmonary nodule detection applying deep learning or machine learning algorithms to the LIDC-IDRI database: a systematic review,” *Diagnostics*, vol. 9, no. 1, p. 29, 2019.

[377] Soares E, Angelov P, Biaso S, Froes M H, and Abe D K, “SARS-CoV-2 CT-scan dataset: a large dataset of real patients CT scans for SARS-CoV-2 identification,” *Medrxiv*, pp. 2020–4, 2020.

[378] Zhang M, Wu Y, Zhang H, Qin Y, Zheng H, Tang W, Arnold C, Pei C, Yu P, and Nan Y, “Multi-site, multi-domain airway tree modeling,” *Med. Image Anal.*, vol. 90, p. 102957, 2023.

[379] Setio A A A, Traverso A, De Bel T, Berens M S, Van Den Bogaard C, Cerello P, Chen H, Dou Q, Fantacci M E, and Geurts B, “Validation, comparison, and combination of algorithms for automatic detection of pulmonary nodules in computed tomography images: the LUNA16 challenge,” *Med. Image Anal.*, vol. 42, pp. 1–13, 2017.

[380] Dorent R, Khajavi R, Idris T, Ziegler E, Somarouthu B, Jacene H, LaCasce A, Deissler J, Ehrhardt J, Engelson S, *et al.*, “LNQ 2023 challenge: benchmark of weakly-supervised techniques for mediastinal lymph node quantification,” *Mach. Learn. Biomed. Imaging*, vol. 3, no. MICCAI 2023 LNQ challenge, pp. 1–15, Jan. 2025, doi: 10.59275/j.melba.2025-d482.

[381] Chitalia R, Pati S, Bhalerao M, Thakur S, Jahani N, Belenky J V, McDonald E S, Gibbs J, Newitt D, and Hylton N, “Expert tumor annotations and radiomic features for the ispy1/acrin 6657 trial data collection,” *Cancer Imaging Arch.*, 2022.

[382] You C, Shen Y, Sun S, Zhou J, Li J, Su G, Michalopoulou E, Peng W, Gu Y, Guo W, *et al.*, “Artificial intelligence in breast imaging: current situation and clinical challenges,” *Exploration*, vol. 3, no. 5, p. 20230007, Oct. 2023, doi: 10.1002/EXP.20230007.

[383] Al-Dhabyani W, Gomaa M, Khaled H, and Fahmy A, “Dataset of breast ultrasound images,” *Data Brief*, vol. 28, p. 104863, 2020.

[384] Luo G, Xu M, Chen H, Liang X, Tao X, Ni D, Jeong H, Kim C, Stock R, Baumgartner M, *et al.*, “Tumor detection, segmentation and classification challenge on automated 3D breast ultrasound: the TDSC-ABUS challenge,” Jan. 26, 2025, *arXiv*: arXiv:2501.15588. doi: 10.48550/arXiv.2501.15588.

[385] Han C, Pan X, Yan L, Lin H, Li B, Yao S, Lv S, Shi Z, Mai J, Lin J, *et al.*, “WSSS4LUAD: grand challenge on weakly-supervised tissue semantic segmentation for lung adenocarcinoma,” Apr. 14, 2022, *arXiv*: arXiv:2204.06455. doi: 10.48550/arXiv.2204.06455.

[386] Li L, Zimmer V A, Schnabel J A, and Zhuang X, “AtrialJSQnet: a new framework for joint segmentation and quantification of left atrium and scars incorporating spatial and shape information,” *Med. Image Anal.*, vol. 76, p. 102303, 2022, doi: 10.1016/j.media.2021.102303.

[387] El‐Rewaidy H, Fahmy A S, Pashakhanloo F, Cai X, Kucukseymen S, Csecs I, Neisius U, Haji‐Valizadeh H, Menze B, and Nezafat R, “Multi‐domain convolutional neural network (MD‐CNN) for radial reconstruction of dynamic cardiac MRI,” *Magn. Reson. Med.*, vol. 85, no. 3, pp. 1195–1208, Mar. 2021, doi: 10.1002/mrm.28485.

[388] Wang C, Lyu J, Wang S, Qin C, Guo K, Zhang X, Yu X, Li Y, Wang F, and Jin J, “CMRxRecon: a publicly available k-space dataset and benchmark to advance deep learning for cardiac MRI,” *Sci. Data*, vol. 11, no. 1, p. 687, 2024.

[389] Andreopoulos A and Tsotsos J K, “Efficient and generalizable statistical models of shape and appearance for analysis of cardiac MRI,” *Med. Image Anal.*, vol. 12, no. 3, pp. 335–357, 2008, doi: 10.1016/j.media.2007.12.003.

[390] Chen Z, Ren H, Li Q, and Li X, “Motion correction and super-resolution for multi-slice cardiac magnetic resonance imaging via an end-to-end deep learning approach,” *Comput. Med. Imaging Graphics*, vol. 115, p. 102389, 2024.

[391] Vukadinovic M, Kwan A C, Li D, and Ouyang D, “GANcMRI: cardiac magnetic resonance video generation and physiologic guidance using latent space prompting,” in *Machine Learning for Health (ML4H)*, PMLR, 2023, pp. 594–606. Accessed: Mar. 25, 2025. [Online]. Available: https://proceedings.mlr.press/v225/vukadinovic23a.html

[392] Ouyang D, He B, Ghorbani A, Yuan N, Ebinger J, Langlotz C P, Heidenreich P A, Harrington R A, Liang D H, and Ashley E A, “Video-based AI for beat-to-beat assessment of cardiac function,” *Nature*, vol. 580, no. 7802, pp. 252–256, 2020.

[393] Qu C, Zhang T, Qiao H, Tang Y, Yuille A L, and Zhou Z, “Abdomenatlas-8k: annotating 8,000 ct volumes for multi-organ segmentation in three weeks,” *Adv. Neural Inf. Process. Syst.*, vol. 36, pp. 36620–36636, 2023.

[394] Zhou H, Lou Y, Xiong J, Wang Y, and Liu Y, “Improvement of deep learning model for gastrointestinal tract segmentation surgery,” *Front. Comput. Intell. Syst.*, vol. 6, no. 1, pp. 103–106, 2023, doi: 10.54097/fcis.v6i1.19.

[395] Pardo J, Liu J, Ramón-Ferrer V, Amador-Domínguez E, and Calleja P, “K-flares: a K-adapter based approach for the FLARES challenge,” in *In Proceedings of the Iberian Languages Evaluation Forum (iberlef 2024), Co-located with the 40th Conference of the Spanish Society for Natural Language Processing (SEPLN 2024), CEURWS. Org*, 2024. Accessed: Mar. 25, 2025. [Online]. Available: https://ceur-ws.org/Vol-3756/FLARES2024_paper1.pdf

[396] Bai J, Zhou Z, Ou Z, Koehler G, Stock R, Maier-Hein K, Elbatel M, Martí R, Li X, and Qiu Y, “PSFHS challenge report: pubic symphysis and fetal head segmentation from intrapartum ultrasound images,” *Med. Image Anal.*, vol. 99, p. 103353, 2025, doi: 10.1016/j.media.2024.103353.

[397] Polat G, Kani H T, Ergenc I, Ozen Alahdab Y, Temizel A, and Atug O, “Improving the computer-aided estimation of ulcerative colitis severity according to mayo endoscopic score by using regression-based deep learning,” *Inflamm. Bowel Dis.*, vol. 29, no. 9, pp. 1431–1439, 2023.

[398] Misawa M, Kudo S, Mori Y, Hotta K, Ohtsuka K, Matsuda T, Saito S, Kudo T, Baba T, and Ishida F, “Development of a computer-aided detection system for colonoscopy and a publicly accessible large colonoscopy video database (with video),” *Gastrointest. Endosc.*, vol. 93, no. 4, pp. 960–967, 2021.

[399] Brummer O, Pölönen P, Mustjoki S, and Brück O, “Integrative analysis of histological textures and lymphocyte infiltration in renal cell carcinoma using deep learning,” *Biorxiv*, pp. 2022–8, 2022.

[400] Hu W, Li C, Li X, Rahaman M M, Ma J, Zhang Y, Chen H, Liu W, Sun C, and Yao Y, “GasHisSDB: a new gastric histopathology image dataset for computer aided diagnosis of gastric cancer,” *Comput. Biol. Med.*, vol. 142, p. 105207, 2022.

[401] Kawai M, Ota N, and Yamaoka S, “Large-scale pretraining on pathological images for fine-tuning of small pathological benchmarks,” in *Medical Image Learning with Limited and Noisy Data*, vol. 14307, Cham: Springer Nature Switzerland, 2023, pp. 257–267. doi: 10.1007/978-3-031-44917-8_25.

[402] Tsai M-J and Tao Y-H, “Deep learning techniques for the classification of colorectal cancer tissue,” *Electronics*, vol. 10, no. 14, p. 1662, 2021, doi: 10.3390/electronics10141662.

[403] van der Graaf J W, van Hooff M L, Buckens C F, Rutten M, van Susante J L, Kroeze R J, de Kleuver M, van Ginneken B, and Lessmann N, “Lumbar spine segmentation in MR images: a dataset and a public benchmark,” *Sci. Data*, vol. 11, no. 1, p. 264, 2024, doi: 10.1038/s41597-024-03090-w.

[404] Sharafi A, Arpinar V E, Nencka A S, and Koch K M, “Development and stability analysis of carpal kinematic metrics from 4D magnetic resonance imaging,” *Skeletal Radiol.*, vol. 54, no. 1, pp. 57–65, Jan. 2025, doi: 10.1007/s00256-024-04687-3.

[405] Desai A D, Schmidt A M, Rubin E B, Sandino C M, Black M S, Mazzoli V, Stevens K J, Boutin R, Ré C, Gold G E, *et al.*, “SKM-TEA: a dataset for accelerated MRI reconstruction with dense image labels for quantitative clinical evaluation,” Mar. 14, 2022, *arXiv*: arXiv:2203.06823. doi: 10.48550/arXiv.2203.06823.

[406] Eskicioglu A M and Fisher P S, “Image quality measures and their performance,” *IEEE Trans. Commun.*, vol. 43, no. 12, pp. 2959–2965, 1995.

[407] Sheikh H R and Bovik A C, “A visual information fidelity approach to video quality assessment,” in *The First International Workshop on Video Processing and Quality Metrics for Consumer Electronics*, sn, 2005, pp. 2117–2128. Accessed: Mar. 26, 2025. [Online]. Available: https://utw10503.utweb.utexas.edu/publications/2005/hrs_vidqual_vpqm2005.pdf

[408] Wang Z and Bovik A C, “A universal image quality index,” *IEEE Signal Process. Lett.*, vol. 9, no. 3, pp. 81–84, 2002.

[409] Korotin A, Egiazarian V, Asadulaev A, Safin A, and Burnaev E, “Wasserstein-2 generative networks,” Dec. 10, 2020, *arXiv*: arXiv:1909.13082. doi: 10.48550/arXiv.1909.13082.

[410] Barratt S and Sharma R, “A note on the inception score,” Jun. 21, 2018, *arXiv*: arXiv:1801.01973. doi: 10.48550/arXiv.1801.01973.

[411] Devlin J, Chang M-W, Lee K, and Toutanova K, “Bert: pre-training of deep bidirectional transformers for language understanding,” in *Proceedings of the 2019 Conference of the North American Chapter of the Association for Computational Linguistics: Human Language Technologies, Volume 1 (long and Short Papers)*, 2019, pp. 4171–4186. Accessed: Mar. 28, 2025. [Online]. Available: https://aclanthology.org/N19-1423/?utm_campaign=The%20Batch&utm_source=hs_email&utm_medium=email&_hsenc=p2ANqtz-_m9bbH_7ECE1h3lZ3D61TYg52rKpifVNjL4fvJ85uqggrXsWDBTB7YooFLJeNXHWqhvOyC

[412] Zhang H, Li X, and Bing L, “Video-LLaMA: an instruction-tuned audio-visual language model for video understanding,” Oct. 25, 2023, *arXiv*: arXiv:2306.02858. doi: 10.48550/arXiv.2306.02858.

[413] Carreira J and Zisserman A, “Quo vadis, action recognition? a new model and the kinetics dataset,” in *Proceedings of the IEEE Conference on Computer Vision and Pattern Recognition*, 2017, pp. 6299–6308. Accessed: Mar. 27, 2025. [Online]. Available: http://openaccess.thecvf.com/content_cvpr_2017/html/Carreira_Quo_Vadis_Action_CVPR_2017_paper.html

[414] Unterthiner T, Steenkiste S van, Kurach K, Marinier R, Michalski M, and Gelly S, “Towards accurate generative models of video: a new metric & challenges,” Mar. 27, 2019, *arXiv*: arXiv:1812.01717. doi: 10.48550/arXiv.1812.01717.

[415] Huang Z, He Y, Yu J, Zhang F, Si C, Jiang Y, Zhang Y, Wu T, Jin Q, Chanpaisit N, *et al.*, “VBench: comprehensive benchmark suite for video generative models,” in *Proceedings of the IEEE/CVF Conference on Computer Vision and Pattern Recognition*, 2024, pp. 21807–21818. Accessed: Mar. 27, 2025. [Online]. Available: https://openaccess.thecvf.com/content/CVPR2024/html/Huang_VBench_Comprehensive_Benchmark_Suite_for_Video_Generative_Models_CVPR_2024_paper.html

[416] Teed Z and Deng J, “RAFT-3D: scene flow using rigid-motion embeddings,” in *Proceedings of the IEEE/CVF Conference on Computer Vision and Pattern Recognition*, 2021, pp. 8375–8384.

[417] Griffin A, McKeown A, Viney R, Rich A, Welland T, Gafson I, and Woolf K, “Revalidation and quality assurance: the application of the MUSIQ framework in independent verification visits to healthcare organisations,” *BMJ Open*, vol. 7, no. 2, p. e014121, Feb. 2017, doi: 10.1136/bmjopen-2016-014121.

[418] Xu H, Ghosh G, Huang P-Y, Okhonko D, Aghajanyan A, Metze F, Zettlemoyer L, and Feichtenhofer C, “VideoCLIP: contrastive pre-training for zero-shot video-text understanding,” Oct. 01, 2021, *arXiv*: arXiv:2109.14084. doi: 10.48550/arXiv.2109.14084.

[419] Budd S, Robinson E C, and Kainz B, “A survey on active learning and human-in-the-loop deep learning for medical image analysis,” *Med. Image Anal.*, vol. 71, p. 102062, 2021, doi: 10.1016/j.media.2021.102062.
